# Ascorbic Acid/Retinol and/or Inflammatory Stimuli’s Effect on Proliferation/Differentiation Properties and Transcriptomics of Gingival Stem/Progenitor Cells

**DOI:** 10.3390/cells10123310

**Published:** 2021-11-25

**Authors:** Karim M. Fawzy El-Sayed, Amira Bittner, Kristina Schlicht, Mohamed Mekhemar, Kim Enthammer, Marc Höppner, Martha Es-Souni, Juliane Schulz, Matthias Laudes, Christian Graetz, Christof E. Dörfer, Dominik M. Schulte

**Affiliations:** 1 Clinic for Conservative Dentistry and Periodontology, School of Dental Medicine, Christian-Albrechts-University of Kiel, 24105 Kiel, Germany; amira.bittner@googlemail.com (A.B.); mekhemar@konspar.uni-kiel.de (M.M.); graetz@konspar.uni-kiel.de (C.G.); doerfer@konspar.uni-kiel.de (C.E.D.); 2 Oral Medicine and Periodontology Department, Faculty of Dentistry, Cairo University, Cairo 11553, Egypt; 3 Stem cells and Tissue Engineering Unit, Faculty of Dentistry, Cairo University, Cairo 11553, Egypt; 4 Institute of Diabetes and Clinical Metabolic Research, School of Medicine, Christian-Albrechts-University of Kiel, 24104 Kiel, Germany; Kristina.Schlicht@uksh.de (K.S.); KimCarina.Enthammer@uksh.de (K.E.); Juliane.Schulz@uksh.de (J.S.); matthias.laudes@uksh.de (M.L.); dominik.schulte@uksh.de (D.M.S.); 5 Division of Endocrinology, Diabetes and Clinical Nutrition, Department of Medicine I, School of Medicine, University Hospital of Schleswig-Holstein, 24105 Kiel, Germany; 6 Institute of Clinical Molecular Biology, School of Medicine, Christian-Albrechts-University of Kiel, 24105 Kiel, Germany; m.hoeppner@ikmb.uni-kiel.de; 7 Department of Orthodontics, School of Dental Medicine, University Clinic Schleswig-Holstein (UKSH), Christian-Albrechts University of Kiel, 24105 Kiel, Germany; Es-souni@kfo-zmk.uni-kiel.de; 8 Cluster of Excellence, Precision Medicine in Chronic Inflammation, School of Medicine, Christian-Albrechts-University of Kiel, 24105 Kiel, Germany

**Keywords:** inflammation, ascorbic acid, retinol, stem cell, gingiva

## Abstract

The present study explored the effects of ascorbic-acid (AA)/retinol and timed inflammation on the stemness, the regenerative potential, and the transcriptomics profile of gingival mesenchymal stem/progenitor cells’ (G-MSCs). STRO-1 (mesenchymal stem cell marker) immuno-magnetically sorted G-MSCs were cultured in basic medium (control group), in basic medium with IL-1β (1 ng/mL), TNF-α (10 ng/mL) and IFN-γ (100 ng/mL, inflammatory-medium), in basic medium with AA (250 µmol/L) and retinol (20 µmol/L) (AA/retinol group) or in inflammatory medium with AA/retinol (inflammatory/AA/retinol group; *n* = 5/group). The intracellular levels of phosphorylated and total β-Catenin at 1 h, the expression of stemness genes over 7 days, the number of colony-forming units (CFUs) as well as the cellular proliferation aptitude over 14 days, and the G-MSCs’ multilineage differentiation potential were assessed. Next-generation sequencing was undertaken to elaborate on up-/downregulated genes and altered intracellular pathways. G-MSCs demonstrated all mesenchymal stem/progenitor cells characteristics. Controlled inflammation with AA/retinol significantly elevated *NANOG* (*p* < 0.05). The AA/retinol-mediated reduction in intracellular phosphorylated β-Catenin was restored through the effect of controlled inflammation (*p* < 0.05). Cellular proliferation was highest in the AA/retinol group (*p* < 0.05). AA/retinol counteracted the inflammation-mediated reduction in G-MSCs’ clonogenic ability and CFUs. Amplified chondrogenic differentiation was observed in the inflammatory/AA/retinol group. At 1 and 3 days, the differentially expressed genes were associated with development, proliferation, and migration (*FOS*, *EGR1*, *SGK1*, *CXCL5*, *SIPA1L2*, *TFPI2*, *KRATP1-5*), survival (*EGR1*, *SGK1*, *TMEM132A*), differentiation and mineral absorption (*FOS*, *EGR1*, *MT1E*, *KRTAP1-5*, *ASNS*, *PSAT1*), inflammation and MHC-II antigen processing (*PER1*, *CTSS*, *CD74*) and intracellular pathway activation (*FKBP5*, *ZNF404*). Less as well as more genes were activated the longer the G-MSCs remained in the inflammatory medium or AA/retinol, respectively. Combined, current results point at possibly interesting interactions between controlled inflammation or AA/retinol affecting stemness, proliferation, and differentiation attributes of G-MSCs.

## 1. Introduction

Initiation of periodontitis generally necessitates the stimulation of the periodontal immune system through a bacterial dysbiosis, consequently setting complex inflammatory cascades in motion, characterized by the liberation of a variety of pro-inflammatory cytokines, mainly tumor necrosis factor-alpha (TNF-α), interleukin (IL)-1 beta (IL-1β), IL-4, IL-6, IL-17 as well as interferon-gamma (IFN-γ) [1,2]. Although such pro-inflammatory response is pivotal in combating the invading pathogens and in boosting subsequent periodontal stem/progenitor cells-mediated reparative/regenerative endeavors, a long-lasting not adequately self-limiting pro-inflammatory insult could detrimentally affect the tooth-supporting and investing cellular components of the periodontium [3]. Clinically, gingival mesenchymal stem/progenitor cells (G-MSCs) are in constant immuno-regenerative crosstalk with their surrounding micro-environment [4,5,6], with controlled and precisely timed pro-inflammatory stimuli exerting positive effects on their stemness and reparative/regenerative attributes [7,8].

Ascorbic acid (AA) and retinol are antioxidants, with a multitude of significant host inflammation-modulatory effects [9,10,11,12] on periodontal disease and the outcome of reparative/regenerative periodontal therapies [13,14]. While AA promotes wound healing and collagen synthesis [12], AA and retinol boost cellular metabolism, proliferation, and differentiation, while impeding apoptosis [15,16,17,18,19]. Chronic periodontitis was found to be associated with a lower retinol intake in young Korean women [9] and low serum levels of a variety of carotenoids, in particular beta-cryptoxanthin and beta-carotene, were demonstrated to be connected with an elevated periodontitis prevalence in a sample of 1258, 60–70-year-old Western European men [10]. Every other day oral administration of all-trans retinoic acid in a *Porphyromonas gingivalis*-induced mice periodontitis model reduced the inflammatory cellular infiltrate, enhanced the T-regulatory cell activation, and arrested further periodontal inflammation-mediated tissue destruction [11].

Most strikingly, recent reports demonstrated the ability of AA and retinol, at specific concentrations, to impact cellular epigenetics, through nuclear bases demethylation, with a resultant de-differentiation of adult cells into pluripotent ones [20,21], a perspective with great potential for periodontal reparative/regenerative endeavors. The current study’s aim was to explore for the first time the effects of AA/retinol in isolation and combined with controlled and timed pro-inflammatory stimulation on stemness, proliferation, Wnt/β-catenin pathway activation, differentiation, and mRNA transcriptomics of G-MSCs in vitro and to elaborate on the associated intracellular pathways.

## 2. Materials and Methods

### 2.1. G-MSCs’ Isolation, Characterization and Multilineage Differentiation

The study’s protocol was reviewed by the Ethical Committee of the Christian-Albrechts University of Kiel, Kiel, Germany (IRB:513/17). Gingival connective tissue cells were isolated from free gingival collars from five healthy patients (Table 1 shows the donors’ age and gender), and STRO-1 immuno-magnetically sorted to obtain G-MSCs as previously described [22]. Colony-forming units (CFUs), multilineage differentiation potential and CD14, CD34, CD45, CD73, CD90, and CD105 stemness marker expression were examined on second passage G-MSCs (FACS-Calibur-E6370 and FACS-Comp5.1.1 software, Becton Dickinson, Franklin Lakes, NJ, USA), as previously described [22].

### 2.2. Experimental Groups

Second passage G-MSCs were cultured in basic medium, consisting of Eagle’s minimum essential medium alpha modification (Sigma-Aldrich GmbH, Hamburg, Germany) supplemented with antibiotics (100 U mL^−1^ penicillin, 100 µg mL^−1^ streptomycin) and 1% amphotericin (all from Biochrom, Berlin, Germany) (control group), in basic medium, with 1 ng/mL IL-1β, 10 ng/mL TNF-α and 100 ng/mL IFN-γ (Pepro Tech Inc., Rocky Hill, NJ, USA) [5,6,8,23,24,25] (inflammatory group), in basic medium with 250 µg/mL AA [21] and 20 µmol/L retinol [26] (AA/retinol group), or in inflammatory medium with 250 µg/mL AA and 20 µmol/L retinol (inflammatory/AA/retinol group). Media were exchanged three times per week.

### 2.3. G-MSCs’ mRNA Expression

*NANOG*, octamer-binding-transcription-factor-4A (*OCT4A*) and sex-determining-region-Y-box-2 (*SOX2*) stemness markers were assessed on mRNA level in the four groups (*n* = 5). mRNA isolation was carried out at 1, 3, 5, and 7 days (RNeasy kit, Qiagen, Hilden, Germany). cDNA was produced from RNA (1 μg/μL) by reverse transcription (QuantiTect Reverse Transcription Kit, Qiagen, Hilden, Germany) in 20 μL reaction mixture (4 pmol of each primer, 10 μL of LightCycler Probes Master mixture (Roche, Indianapolis, IN, USA) and 5 μL specimen cDNA). Real-time polymerase chain reaction (rt-PCR; LightCycler 96 Real-Time PCR System, Roche Molecular Biochemicals, Indianapolis, IN, USA) was performed. Nineteen potential reference genes (*18S*, *ACTB*, *ALAS*, *β-2M*, *β-Globin*, *G6PDH*, *GAPDH*, *GUSB*, *HPRT1*, *IPO8*, *PBGD*, *PGK1*, *PPIA*, *RPL13A*, *RPLP0*, *SDHA*, *TBP*, *TFRC*, and *YWHAZ*) were pre-examined for the most suitable reference gene, which would not be altered by the experiment (NormFinder). Apart from *PGK1*, all tested genes were altered. Thus, *PGK1* (a housekeeping gene) was deemed suitable to be utilized (Table 2). Relative quantification of all genes under examination was performed using the 2^-ΔΔCt method in triplicate and averaged.

### 2.4. ELISA

*SOX2*, *OCT4*, and *NANOG* were measured using simple step ELISA Kits (ABCAM, Cambridge, UK). G-MSCs (*n* = 5) were cultivated on six-well plates and stimulated according to the defined groups, followed by PBS washing, 600 μL lysis buffer addition, and aliquoting. ELISA measurements were carried out following the manufacturer’s instructions. Quantitation of bound analyte was achieved photometrically through detection of the colored oxidized TMB product at 450 nm (µQuant-spectrophotometer, BioTek; Mikrowin-software, Mikrotek Laborsysteme, Overath, Germany).

For evaluation of phosphorylated (pβ-catenin, pS45 ELISA Kit, Abcam, Cambridge, UK) and total (tβ-catenin, Abcam, Cambridge, UK) intracellular β-catenin levels, 8 × 10^4^ G-MSCs were cultivated per well in six-well plates until reaching 85% confluence. Subsequently, G-MSCs were stimulated for one hour in the different groups and washed with 3 × 350 µL 1× wash buffer PT followed by the addition of 350 μL chilled 1× cell extraction Buffer. Fifty microliters standard or sample was mixed with 100 μL pβ-catenin or tβ-catenin detection antibody and incubated in the dark on a plate shaker (400 rpm, 37 °C, 5% CO_2_, 15 min), followed by Stop Solution (100 μL) and 450 nm optical density (OD) measurements (MultiskanGO Microplate Spectrophotometer, Thermo Fisher, Langenselbold, Germany). Intracellular %pβ-catenin and %tβ-catenin were determined employing standard curves. All experiments were performed in duplicates and averaged.

### 2.5. mRNA Next-Generation Sequencing

mRNA from three different probands grown in control or inflammatory medium and subjected to either treatment with AA/retinol or not were extracted. Differential expression analysis (DEA) was conducted on days 1 and 3 of exposure (*n* = 24). Sequencing of samples was performed at the next-generation sequencing (NGS) lab at the Institute of Clinical Molecular Biology (IKMB) in Kiel on an Illumina MiSeq. Raw FastQ files were aligned, quality controlled, and transformed into read counts, using Nextflow nfcore/RNAseq pipeline https://nf-co.re/rnaseq (accessed on 22 February 2021) [27]. Read counts were analyzed in Rv3.6.2 using edgeR [28] and DeSEQ2 Packages [29]. Gene counts were rlog transformed and visualized in heatmaps in DeSEQ2. Differential expression analysis (DEA) was carried out in edgeR, using the Quasi-likelihood F-test (QLF) function, which gives stricter error rate control by accounting for the uncertainty in dispersion estimation and allows for multi-factor contrast, while controlling the individual subjects, thus correcting for inter-individual variation in the samples [30]. Contrasts were modeled separately for effects of medium (control or inflammatory medium) and treatment (AA/retinol or not) on days 1 and 3, as well as grouped (medium with treatment) together leading to three different contrasts in each experiment day. To control the false discovery rate (FDR), the Benjamini–Hochberg method was employed to correct for multiple testing. Kyoto Encyclopedia of Genes and Genomes (KEGG) pathway analysis [31] was carried out for each day of the experiment and visualized for differently expressed genes, using R-package “clusterprofiler” [32].

### 2.6. CFUs and Cellular Proliferation

G-MSCs passage (1 × 10^4^) were cultivated per well per group in 24-well culture plates (*n* = 5). Cellular counts were established daily by two independent examiners for 14 consecutive days and cellular proliferation curves were plotted for the different groups.

Second passage 1.63/cm^2^ G-MSCs of the different groups were seeded in 10 cm diameter dishes (*n* = 5). On the 14th day, cell cultures were fixed using chilled 100% methanol and stained with 0.1% crystal violet for 10 min. Two independent examiners counted the CFUs under a phase-contrast inverted microscope, where aggregations of ≥50 cells were considered as a colony.

### 2.7. Multilineage Potential of Stimulated G-MSCs

For five days, G-MSCs were pre-stimulated in the experimental groups (*n* = 5). Subsequently, they underwent osteogenic (14 days), adipogenic (21 days), or chondrogenic (35 days) differentiation in an inflammation-free environment with their respective inductive media as described above. Runt-related transcription factor 2 (RUNX2) and alkaline phosphatase (ALP) mRNA expression as well as qualitative and quantitative Alizarin-Red staining was conducted [33]. Lipoprotein lipase (LPL) and proliferator-activated receptor gamma (PPAR-ɣ) mRNA expression as well as quantitative and qualitative Oil-Red-O evaluation were examined to confirm adipogenic differentiation [34]. Aggrecan (ACAN) mRNA expression and Alcian-Blue/nuclear-fast-red staining quantification were evaluated as evidence for chondrogenic differentiation [35]. All PCR primers were supplied by Roche and the real-time PCR was conducted as described above in triplicate and averaged (Table 2).

### 2.8. Statistical Analysis

Normality of the data was examined, employing the Shapiro–Wilk Test. Data proved to be not normally distributed. Hence, differences in %tβ-catenin, %pβ-catenin, CFUs, mRNA expressions, and quantitative adipogenic, osteogenic, and chondrogenic differentiation between the experimental groups were examined, using the Friedman test (SPSS 23, IBM, Chicago, IL, USA). The significance level was set at *p* ≤ 0.05.

## 3. Results

### 3.1. Characterization of G-MSCs

**Figure 1:**
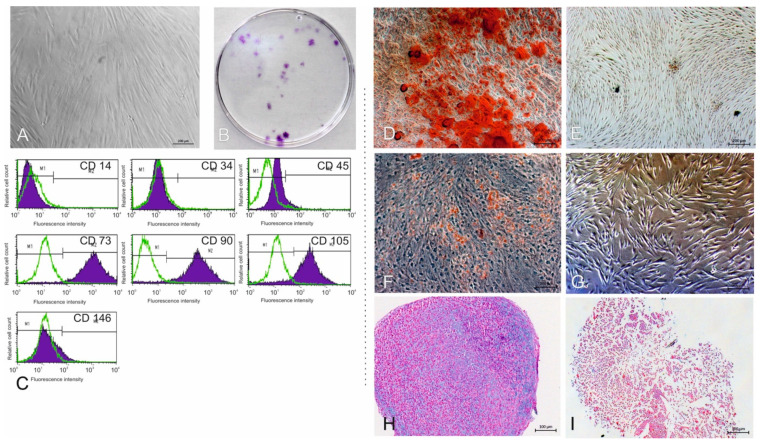
Phase contrast inverted microscopic picture of gingival cells growing out from a gingival connective tissue specimen (**A**). G-MSCs’ colony-forming units (CFUs) (**B**). G-MSCs surface markers’ expression flowcytometrically (**C**). Osteogenic induction of G-MSCs (Alizarin-Red stained; (**D**)) and respective controls (**E**) Adipogenic induction of G-MSCs (Oil-Red-O stained; (**F**)) and respective controls (**G**). Chondrogenic induction of G-MSCs (Alcian Blue/acid-fast-red staining; (**H**)) and respective controls (**I**).

Fibroblast-like cell clusters grew out of adherent gingival connective tissue masses (Figure 1A). G-MSCs exhibited classical CFUs (Figure 1B), and were CD14^−^, CD34^−^, CD45^−^, CD73^+^, CD90^+^, and CD105^+^ (Figure 1C). Through osteogenic induction, G-MSCs deposited Alizarin-Red-positive calcified deposits, in distinction to their controls (Figure 1D,E). Adipogenic induction of G-MSCs formed Oil-Red-O-positive intracellular inclusion bodies, in distinction to their controls (Figure 1F,G). Chondrogenic induction of G-MSCs deposited Alcian-Blue/acid-fast-red-positive glycosaminoglycans, in distinction to their controls (Figure 1H,I).

### 3.2. Stemness Markers’ Expression

**Figure 2:**
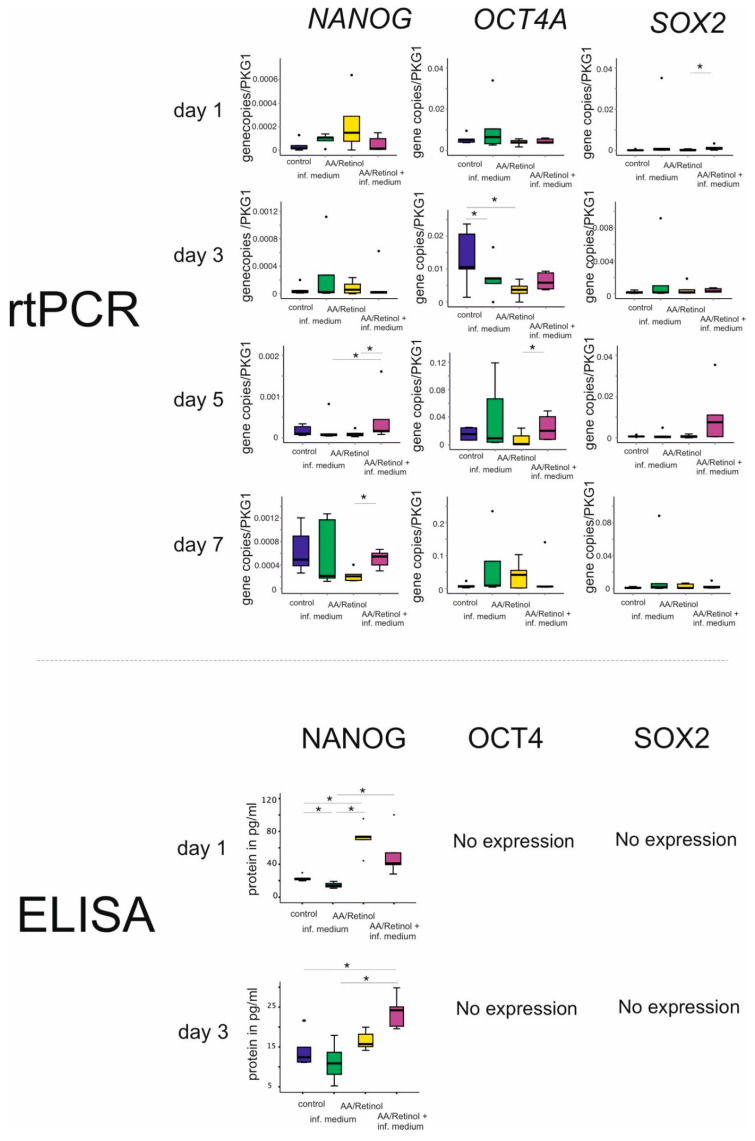
mRNA and protein expressions of stemness markers (*NANOG*, *OCT4A*, *SOX2*) in G-MSCs challenged by AA/retinol and inflammation at 1, 3, 5, and 7 days (box and whisker plots with medians/quartiles). Significant differences denoted with asterisks (*n* = 5; * *p* < 0.05, Friedman test). Abbreviations: *SOX2*: sex-determining region Y-box 2; *OCT4A*: octamer-binding transcription factor 4A.

Regarding the expression of stemness genes, significant differences between the groups were notable at day 1 for *SOX2* expression, at day 5 for *OCT4A* expression and at days 5 and 7 for *NANOG* expression (*p* < 0.05). On the protein level, at day 1, significant differences were further evident for *NANOG* expression between groups (*p* < 0.05), with a synergistic effect of AA/retinol and inflammation evident only at day 3 (*p* < 0.05, Friedman). No expressions were detected for *SOX2* or *OCT4* on the protein level (Figure 2).

### 3.3. mRNA Next Generation Sequencing

**Figure 3:**
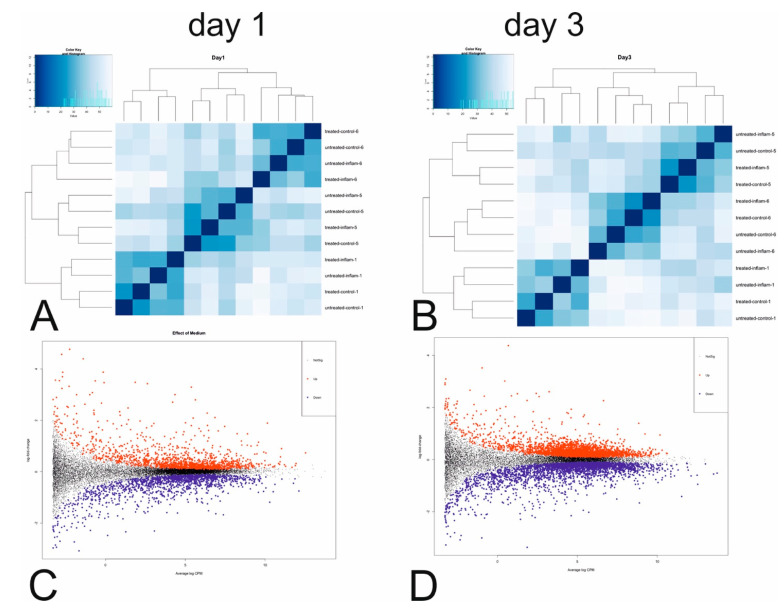

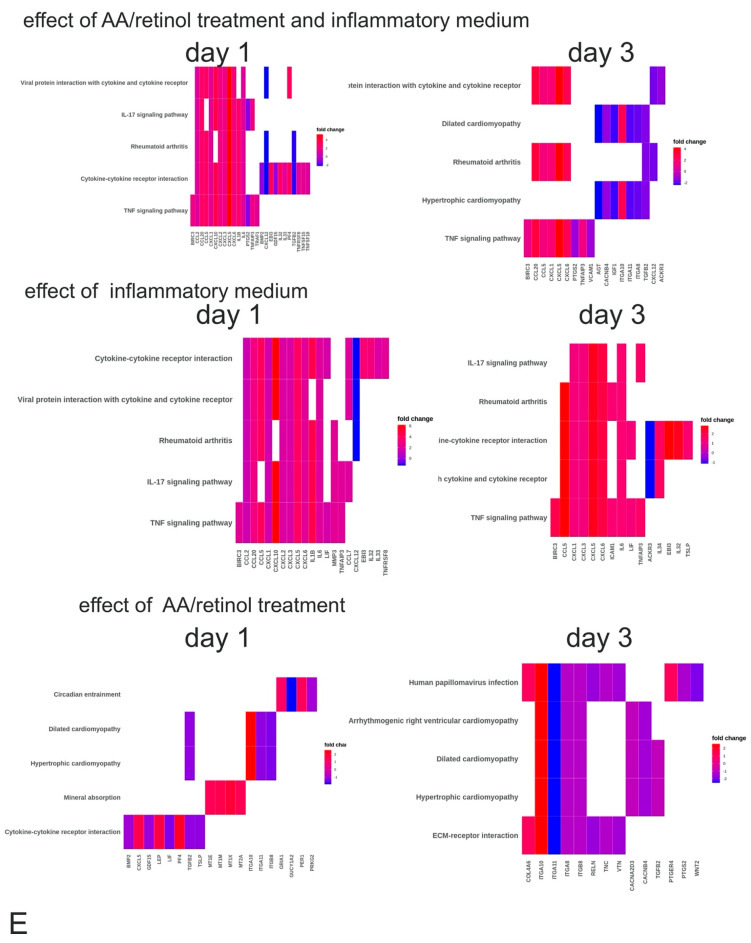
Visualization of gene counts and differentially expressed genes: (**A**) Heatmap of rlog-transformed gene counts for all samples on day 1 of the experiment. Labels refer to type of medium, type of treatment and proband number. (**B**) Heatmap of rlog transformed gene counts for all samples on day 3 of the experiment. Labels refer to type of medium, type of treatment and proband number. (**C**) MD plot of differentially expressed genes for the overall effect of growth medium on day 1 of the experiment, type of treatment and proband id. (**D**) MD plot of differentially expressed genes for the effect of AA/retinol treatment on day 1 of the experiment, type of growth medium and proband id. (**E**) Top five significantly represented pathways for the effects of AA/retinol treatment (lower section), medium (middle section) and the combined effect of both (upper section). Three patients were tested (*n* = 3) per day (days 1 and 3). Four replicates were carried out for each measurement.

Rlog-transformed gene counts showed a clear cluster pattern depending on probands (Figure 3A,B and Figure S1), appearing to be the main source of variation in the gene expression profiles. Further analysis was performed in edgeR, allowing for complex multi-factor designs and adjustment for the individual effect of different probands. Table 3 provides an overview of the top three differentially expressed (DE) genes on days 1 and 3 (A full list of DE genes for each effect is provided in Supplementary Table S1).

For the combined effect of inflammatory medium and AA/retinol treatment, adjusted for the effect of proband, DEA resulted in 803 DE genes on day 1 and 729 DE genes on day 3. On day 1, the top three genes for this effect were the tissue factor pathway inhibitor (*TFPI1*), Folistatin (*FST*), and FKBP proryl isomerase (*FKBP5*). On day 3, the top DE genes were *FKBP5* and two genes involved in the transfer and synthesis of amino acids serine (phosphoserine aminotransferase—*PSAT1*) and asparagine (asparagine synthetase—*ASNS*). When looking at the effect of inflammatory medium solely on day 1, adjusted for proband and treatment, a total of 161 genes were significantly downregulated and 182 genes were significantly upregulated. On day 3, this changed to 99 genes being significantly downregulated and 90 genes significantly upregulated (Figure 3C,D). The top three DE genes on day 1 were, again *TFPI1*, followed by the C-X-C motif chemokine ligand 5 (*CXCL5*) and cathepsin S (*CTTS*). On day 3, the top upregulated genes were *CD74,* followed by *CTTS* and the keratin-associated protein (*KRATP1-5*), a gene that is associated with developmental biology. Finally, when considering the effect of treatment solely on day 1, adjusted for proband and medium, a total of 182 genes were significantly downregulated and 91 genes were significantly upregulated. On day 3, this changed to 245 genes, being significantly downregulated and 104 genes significantly upregulated. The top 3 DE genes on day 1 were *FKPB5*, *FST*, and metallothionein 1E (*MT1E*). On day 3, the top three genes were again *FKBP5*, *FST*, and *SIPA1L2*).

To validate this observation and to further explore the involvement of our entire DE gene list in cellular pathways, an overrepresentation analysis based on KEGG pathways was performed. Results of the pathway analysis for effects of medium, treatment, and their combined effect are shown in Figure 3E (figure shows top five overrepresented pathways only). Supplementary Table S2 provides the full list of overrepresented KEGG pathways for each effect. To validate the results, we additionally performed functional pathway analyses, using Reactome and Wikipathway databases (Supplementary Table S3, for the combined effect of treatment and medium). For obvious reasons, curation and annotation of pathways differ between the platforms. Yet, interesting commonalities with regard to the activation of interleukin signaling and chemokine binding pathways (KEGG, Reactome, and Wikipathways) and mineral absorption (KEGG and Wikipathways) were observed.

### 3.4. Intracellular β-Catenin

**Figure 4:**
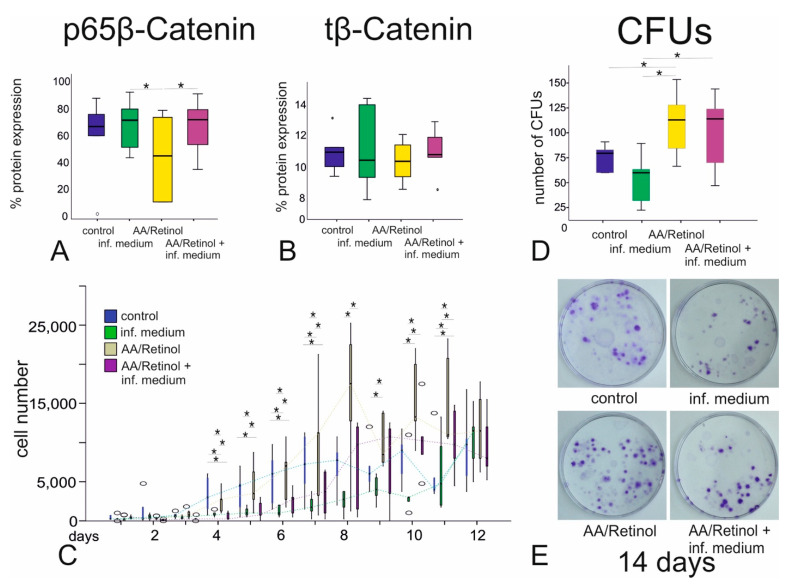
β-catenin expression, cellular proliferation and CFUs following AA/retinol and inflammatory stimulation of G-MSCs: ELISA examination of the phosphorylated (pβ-catenin) and total (tβ-catenin) intracellular β-catenin, following G-MSCs’ challenging by AA/retinol and inflammation ((**A**,**B**); box and whisker plots with medians/quartiles). GMSCs’ cell proliferatory graph of the AA/retinol and inflammation stimulated groups over 14 days ((**C**); box and whisker plots with medians/quartiles). CFUs assay/CFUs’ numbers following G-MSCs’ stimulation via ascorbic acid and inflammation ((**D**); box and whisker plots with medians/quartiles). Significant differences denoted with asterisks (*n* = 5, * *p* < 0.05; Friedman test). Representative CFUs of the four experimental groups (**E**). CFUs; colony-forming units, pβ-catenin; phosphorylated β-catenin, tβ-catenin; total (tβ-catenin) intracellular β-catenin.

Significantly lower intracellular pβ-catenin was evident in the AA/retinol—compared to the inflammatory/AA/retinol– and the inflammatory group (*p* < 0.05, Figure 4A). Intracellular tβ-catenin was similar between all groups (*p* > 0.05, Friedman, Figure 4B).

### 3.5. CFUs and Cellular Proliferation

Significant inter-group differences in cellular counts were evident from days 4 to 11, with the AA/retinol group demonstrating the highest cellular counts, followed by the control-, the inflammatory/AA/retinol—and finally the inflammatory group (*p* < 0.05). At 14 days, the numbers of CFUs were significantly higher in the AA/retinol—followed by the inflammatory/AA/retinol group (*p* < 0.05, Friedman, Figure 4C–E).

### 3.6. Stimulated G-MCSs’ Multilineage Differentiation

**Figure 5:**
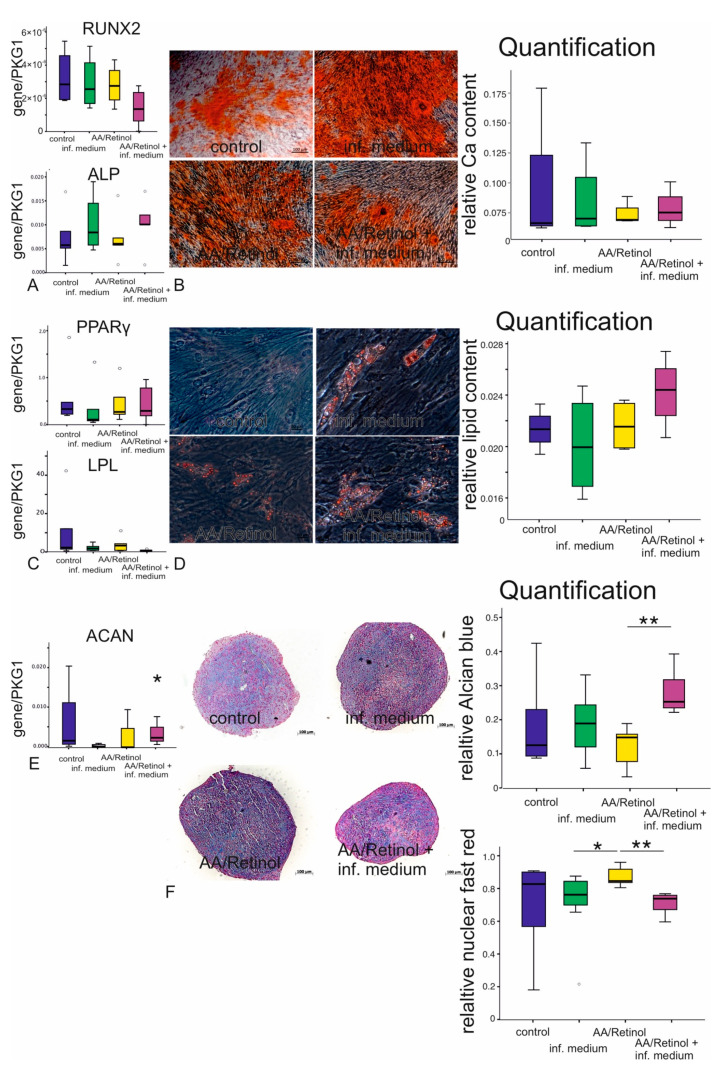
G-MSCs multilineage differentiation following stimulation by AA/retinol and inflammation: Gene expressions of *ALP* and *RUNX2* following a 14-day osteogenic stimulation ((**A**); box and whisker plots with medians/quartiles). Ca^2+^ quantification and Alizarin-Red staining following a 14-day osteogenic induction of ascorbic acid and inflammation stimulated G-MSCs ((**B**); box and whisker plots with medians/quartiles). *LPL* and *PPARɣ* gene expression after 21 days of adipogenic stimulation of ascorbic acid and inflammation challenged G-MSCs ((**C**); box and whisker plots with medians/quartiles). Oil-Red-O staining and lipid amount quantification of ascorbic acid and inflammation stimulated G-MSCs after 21 days of adipogenic stimulation ((**D**); box and whisker plots with medians/quartiles). *ACAN* gene expression following a 35-day chondrogenic induction of ascorbic acid and inflammation stimulated G-MSCs ((**E**); box and whisker plots with medians/quartiles). Alcian-blue/nuclear-fast-red staining of ascorbic acid and inflammation stimulated G-MSCs following a 35-day chondrogenic induction (**F**) (*n* = 5, a circle represents an outlier, * *p* < 0.05, ** *p* < 0.01; Friedman test).

G-MSCs in all experimental groups exhibited a remarkable multilineage differentiation aptitude, with a heightened differentiation potential irrespective of the treatment group. However, the chondrogenic differentiation appeared to be significantly enhanced by the synergistic effect of inflammation and AA/retinol application in the inflammatory/AA/retinol group, compared to AA/retinol alone, with significantly higher *ACAN* expression and glycosaminoglycans deposition observed (*p* < 0.05, Friedman, Figure 5).

## 4. Discussion

Periodontal reparative/regenerative approaches rely chiefly on the reiteration of developmental procedures, involving stem/progenitor cells’ proliferation, differentiation, and maturation [36]. Clinically, these primary events occur under inflamed periodontal micro-environmental conditions, with inflammatory cytokines stage-managing the path of events [2,37,38]. Apart from their important roles in periodontal repair/regeneration [13,14,39,40], AA and retinol exert anti-oxidative effects against periodontitis-induced tissue damages [41,42], demonstrate immunomodulatory capabilities on stem/progenitor cells, dendritic cells, macrophages, T- and B-cells, and markedly downregulate IL-1α, IL-1β, IL-6, TNF-β, and nitric oxide release [43,44,45,46]. Most importantly, AA and retinol at specific concentrations, which were employed in the current investigation, could drive cellular reprogramming/de-differentiation and pluripotency [19,20,47].

In accordance with earlier investigations [22,48,49,50,51], G-MSCs exhibited all characteristic mesenchymal stem/progenitor cells’ traits [52]. In line with a multitude of studies, revealing the positive stimulatory effects of local, controlled, and well-timed micro-environmental pro-inflammatory conditions on G-MSCs’ reparative/regenerative attributes [3,8,53], G-MSCs were challenged by periodontal pro-inflammatory cytokines; explicitly IL-1β, TNF-α, and IFN-γ, by AA/retinol or their combination and their stemness, proliferation, differentiation potentials, mRNA transcriptomics, and associated gene pathways examined.

Although differences in *NANOG*, *OCT4A*, and *SOX2* mRNA expressions were detectable between the groups at different time points, only *NANOG* was detectable on protein level, in line with earlier reports on dissimilar *NANOG*, *OCT4A*, and *SOX2* protein and mRNA expression dynamics within mesenchymal stem/progenitor cells [54,55,56]. This AA/retinol-induced increase in the *NANOG*, especially in the presence of controlled inflammatory stimuli at 3 days, could be ascribed primarily to the capability of AA and retinol to activate the ten-eleven translocation (TET) DNA demethylases, initiating intracellular epigenetic reprogramming with pluripotency amplification [20,57]. The observed synergistic effect suggests that controlled inflammation could have augmented this AA/retinol-mediated effect. Pro-inflammatory stimuli further appeared to increase the AA/retinol-mediated decrease in phosphorylated β-catenin levels intracellularly, restoring the G-MSCs’ stemness [58] and differentiation capacity [59].

According to the mRNA NGS results, it was noticeable that the top three differentially expressed genes for all effects could be grouped under five categories, namely genes associated with developmental biology, cell proliferation, mitosis, and migration (*FOS*, *EGR1*, *SGK1*, *CXCL5*, *SIPA1L2*, *TFPI2*, *KRATP1-5*), with cell survival (*EGR1*, *SGK1*, *TMEM132A*), with cell differentiation and mineral absorption (*FOS*, *EGR1*, *MT1E*, *KRTAP1-5*, *ASNS*, *PSAT1*), with inflammation and MHC-class-II antigen processing (*PER1*, *CTSS*, *CD74*) and intracellular pathway activation (*FKBP5*, *ZNF404*). On day 1, the KEGG pathways of the combined effect of treatment (AA/retinol or not) and medium (inflammation) were mainly characterized by an overexpression of genes in the C-motif chemokine ligand family (*CCL* and *CXCL*). The top five activated KEGG pathways affected the IL-17 and TNF signaling pathway, and cytokine/cytokine receptor interaction. On day 3, the overexpression of C-Motif pathways remained. However, a downregulation of genes in the cardiomyopathy pathways, primarily characterized by genes from the alpha integrin family (*ITGA10*, *ITGA11*, *ITGAB*), which bind collagen and are involved in the degradation of the extracellular matrix [60,61], was observed. Examining exclusively the effect of inflammatory medium, on day 1 the top five KEGG pathways were identical to those of the combined effect on day 1, although fold changes differed slightly. For the AA/retinol effect, on day 1 an under-expression of genes in the integrin-alpha family was notable, with activation of genes in the mineral absorption pathway and overexpression of genes in the Metallothionein family (*MT1X*, *MT1E*, etc.) [62]. On day 3, activation of the focal adhesion and ECM receptor interaction pathways was observed, both of which regulate important biological processes on the cellular level including cellular differentiation, proliferation, motility, and adhesion [61,63]. Broadly speaking, the effect of inflammation seemed to lead to fewer activated genes the longer the cells remained in the inflammatory medium, while the effect of treatment induced activation of more genes the longer the cells were stimulated via AA/retinol, thus endorsing a positive impact of short-termed inflammatory stimuli with a longer AA/retinol stimulation.

Similar to earlier investigations [53,64,65], AA/retinol augmented G-MSCs’ cellular proliferation, especially between the 4th and 11th day, an effect that was clearly attenuated by a combination with pro-inflammatory stimuli. The observed proliferation-inducing effect could be ascribed to AA/retinol-induced upregulated gene expression of *SIPA1L2* and *TFPI2* as well as AA’s ability to suppress cellular growth arrest encoding genes, namely growth arrest/DNA damage-inducible 45α (Gadd45a) and apoptosis inducing genes, namely caspase-1 [44] with an upregulation of the proliferation-related Fos-transcriptional factor [66]. Although, inflammatory stimuli, especially longer-term TNF-α challenges, could induce self-senescence of stem/progenitor cells, especially in the presence of IFN-γ, through changing the IFN-γ-activated, non-apoptotic form of TNF receptor superfamily member 6 (Fas) signaling into a caspase 3- and caspase 8-associated pro-apoptotic cascade [67], significantly higher CFUs were observed over 14 days in the AA/retinol as well as the inflammation/AA/retinol group, demonstrating that AA/retinol could counteract the long-term detrimental effects of inflammation, maintaining the G-MSCs’ colonogenic self-renewal and CFUs production at low cellular densities.

AA and retinol are generally characterized by their ability to modulate cell growth, metabolism, and morphogenesis during osteogenesis [26,68,69] and extracellular matrix production [16]. Similar to earlier studies, inflammatory cytokines or AA/retinol short-term pre-stimuli did not attenuate the subsequent G-MSCs’ characteristic multilineage differentiation potentials [53,65]. Yet, the results regarding the osteogenic differentiation should still be interpreted with caution, taking into consideration that osteogenic media normally contain a specific concentration of AA, which could have possibly masked any effect between the groups. Particularly their conjoint presence appeared beneficial regarding the G-MSCs’ chondrogenic differentiation capacity. In this context, the activation of genes of the mineral absorption pathway (*MT1X*, *MT1E*, *KRTAP1-5*, *PSAT1*) and the downregulation of genes of the alpha integrin family (*ITGA10*, *ITGA11*, *ITGAB*) described above could have significantly contributed to this synergistic effect.

## 5. Conclusions

Combined, current results point at altered G-MSCs’ characteristics in the presence of controlled inflammation or AA/retinol. Apart from the isolated modulatory effects of inflammation or AA/retinol on G-MSCs, a synergistic effect of their conjoint presence on the expression of the *NANOG* stemness marker was observed. The presence of AA/retinol could counteract the inflammation-induced cellular senescence and maintain the G-MSCs’ clonogenic abilities. On the other hand, controlled inflammation could restore the AA/retinol-mediated reduction in intracellular phosphorylated β-catenin as well as enhance the AA/retinol-mediated G-MSC’s chondrogenic differentiation potential. The observed effects were associated with the activation of a multitude of differentially expressed genes associated with development, proliferation and migration, survival, differentiation and mineral absorption, inflammation, and MHC-II antigen processing as well as intracellular pathway activation, with less as well as more genes activated the longer the cells remained in the inflammatory medium or AA/retinol, respectively.

## Figures and Tables

**Table 1:** Donors’ gender and age.

Donor’s Number	Gender	Age
1	Male	18
2	Female	20
3	Female	19
4	Male	22
5	Male	20

**Table 2:** Real-time PCR primers (Roche, Indianapolis, IN, USA).

Gene	Gene Symbol	Accession ID	Assay ID
*RUNX2*	RUNX2 *H. sapiens*	ENST00000359524	113380
*ACAN*	ACAN *H. sapiens*	ENST00000439576	138057
*ALP*	ALP *H. sapiens*	ENST00000374840	103448
*LPL*	LPL *H. sapiens*	ENST00000311322	113230
*NANOG*	NANOG *H. sapiens*	ENST00000229307	148147
*OCT4A*	OCT4 *H. sapiens*	ENST00000259915	113034
*PGK1*	PGK1 *H. sapiens*	ENST00000373316	102083
*PPARɣ*	PPARɣ *H. sapiens*	ENST00000287820	110607
*SOX2*	SOX2 *H. sapiens*	ENST00000325404	111867

Abbreviations: *ACAN*: Aggrecan; *ALP*: alkaline phosphatase; *LPL*: lipoprotein lipase; *OCT4A*: octamer-binding transcription factor 4A; *PGK1*: Phosphoglycerate kinase-1; *PPARɣ*: proliferator-activated receptor gamma; *RUNX2:* Runt-related transcription factor 2; *SOX2*: sex-determining region Y-box 2.

**Table 3:** Top three differentially expressed genes for the effect of growth medium, AA/retinol treatment, as well as the combined effect of inflammatory medium and AA/retinol treatment. Effects have been adjusted for the influence of different probands. LogFC = log Fold Change, LogCPM = log counts per million. Correction for multiple testing was performed with the Benjamini–Hochberg method, significance level was set to FDR < 0.05.

			Ensemble	Entrez ID	Gene Name	LogFC		*p*-Value	FDR
						Medium	Treatment		
Day 1	Treatment + medium	Gen 1	ENSG00000105825	7980	TFPI1	1.46		1.82 × 10^−13^	1.41 × 10^−9^
		Gen 2	ENSG00000134363	10468	FST	−0.43		5.48 × 10^−13^	2.0 4× 10^−9^
		Gen 3	ENSG00000096060	2289	FKBP5	1.54		6.66 × 10^−13^	2.04 × 10^−9^
	Medium	Gen 1	ENSG00000105825	7980	TFPI2	1.98		5.85 × 10^−14^	9.06 × 10^−10^
		Gen 2	ENSG00000163735	6374	CXCL5	3.86		5.63 × 10^−12^	4.36 × 10^−8^
		Gen 3	ENSG00000163131	1520	CTSS	2.27		8.82 × 10^−12^	4.56 × 10^−8^
	Treatment	Gen 1	ENSG00000096060	2289	FKBP5		2.00	1.29 × 10^−13^	2.00 × 10^−9^
		Gen 2	ENSG00000134363	10468	FST		−1.52	1.74 × 10^−12^	1.25 × 10^−8^
		Gen 3	ENSG00000169715	4493	MT1E		1.33	2.42 × 10^−12^	1.25 × 10^−8^
Day 3	Treatment + medium	Gen 1	ENSG00000096060	2289	FKBP5	2.00		1.67 × 10^−12^	1.21 × 10^−8^
		Gen 2	ENSG00000135069	29968	PSAT1	1.17		1.18 × 10^−12^	1.22 × 10^−8^
		Gen 3	ENSG00000134363	10468	ASNS	0.38		2.39 × 10^−11^	1.22 × 10^−8^
	Medium	Gen 1	ENSG00000019582	972	CD74	1.89		3.20 × 10^−12^	4.88 × 10^−8^
		Gen 2	ENSG00000163131	1520	CTSS	1.53		9.24 × 10^−11^	7.05 × 10^−7^
		Gen 3	ENSG00000221852	83895	KRTAP1-5	1.12		4.27 × 10^−9^	1.83 × 10^−5^
	Treatment	Gen 1	ENSG00000096060	2289	FKBP5		2.52	3.32 × 10^−14^	5.05 × 10^−10^
		Gen 2	ENSG00000134363	10468	FST		−1.83	2.04 × 10^−11^	1.55 × 10^−7^
		Gen 3	ENSG00000116991	57568	SIPA1L2		−1.40	4.48 × 10^−11^	2.28 × 10^−7^

## Data Availability

The data presented in this study are available on request from the corresponding author.

## Supplementary Materials

The following are available online at https://www.mdpi.com/article/10.3390/cells10123310/s1, Table S1: A full list of differentially expressed genes for each effect. Table S2: A full list of overrepresented KEGG pathways for each effect. Table S3: A side by side comparison of overrepresented KEGG, WIKIPATHWAYS and REACTOME pathways for each effect. Figure S1: PCA plot of gene expression data. Colors denote treatment with AA/retinol or no treatment. Shapes denote control or inflammation medium.
