# Supplementary material for: Ascorbic Acid/Retinol and/or Inflammatory Stimuli’s Effect on Proliferation/Differentiation Properties and Transcriptomics of Gingival Stem/Progenitor Cells
Source: Cells. 2021 Nov 25;10(12):3310. doi: 10.3390/cells10123310 (PMC8699152; doi:10.3390/cells10123310)
Supplement: Supplementary file 1 [file cells-10-03310-s001.zip › cells-1436458 supplementary/cells-1436458 Table S1.pdf]

Effect	Geneid	gene_name	gene_id	LFC	PValue	FDR
Treatment & Medium: Day 1	ENSG00000105825	TFPI2	7980	1.47	1.82E-13	1.41E-09
Treatment & Medium: Day 1	ENSG00000134363	FST	10468	-0.43	5.49E-13	2.04E-09
Treatment & Medium: Day 1	ENSG00000096060	FKBP5	2289	1.54	6.66E-13	2.04E-09
Treatment & Medium: Day 1	ENSG00000163131	CTSS	1520	1.74	7.79E-13	2.04E-09
Treatment & Medium: Day 1	ENSG00000163735	CXCL5	6374	5.25	7.91E-13	2.04E-09
Treatment & Medium: Day 1	ENSG00000070669	ASNS	440	0.41	1.33E-12	2.95E-09
Treatment & Medium: Day 1	ENSG00000169715	MT1E	4493	1.74	1.07E-11	2.07E-08
Treatment & Medium: Day 1	ENSG00000108602	ALDH3A1	218	-2.30	2.41E-11	4.11E-08
Treatment & Medium: Day 1	ENSG00000186417	GLDN	342035	-1.99	2.65E-11	4.11E-08
Treatment & Medium: Day 1	ENSG00000179094	PER1	5187	1.42	4.07E-11	5.72E-08
Treatment & Medium: Day 1	ENSG00000198074	AKR1B10	57016	-3.26	4.46E-11	5.72E-08
Treatment & Medium: Day 1	ENSG00000168079	SCARA5	286133	0.40	5.12E-11	5.72E-08
Treatment & Medium: Day 1	ENSG00000128165	ADM2	79924	0.19	5.17E-11	5.72E-08
Treatment & Medium: Day 1	ENSG00000046653	GPM6B	2824	2.01	1.05E-10	1.02E-07
Treatment & Medium: Day 1	ENSG00000188487	INSC	387755	2.66	1.05E-10	1.02E-07
Treatment & Medium: Day 1	ENSG00000006118	TMEM132A	54972	1.16	1.72E-10	1.57E-07
Treatment & Medium: Day 1	ENSG00000117594	HSD11B1	3290	3.34	2.07E-10	1.78E-07
Treatment & Medium: Day 1	ENSG00000204941	PSG5	5673	-1.32	2.56E-10	2.06E-07
Treatment & Medium: Day 1	ENSG00000120708	TGFBI	7045	-1.55	2.66E-10	2.06E-07
Treatment & Medium: Day 1	ENSG00000137869	CYP19A1	1588	-0.41	5.03E-10	3.71E-07
Treatment & Medium: Day 1	ENSG00000041982	TNC	3371	0.55	5.36E-10	3.78E-07
Treatment & Medium: Day 1	ENSG00000185565	LSAMP	4045	-1.74	5.74E-10	3.86E-07

Treatment & Medium: Day 1	ENSG00000101057	MYBL2	4605	0.68	6.12E-10	3.95E-07
Treatment & Medium: Day 1	ENSG00000092621	PHGDH	26227	-0.16	6.56E-10	4.06E-07
Treatment & Medium: Day 1	ENSG00000147852	VLDLR	7436	-0.98	7.33E-10	4.37E-07
Treatment & Medium: Day 1	ENSG00000091490	SEL1L3	23231	-1.50	8.74E-10	5.01E-07
Treatment & Medium: Day 1	ENSG00000132718	SYT11	23208	-0.21	1.06E-09	5.89E-07
Treatment & Medium: Day 1	ENSG00000100739	BDKRB1	623	-0.04	1.14E-09	6.10E-07
Treatment & Medium: Day 1	ENSG00000120337	TNFSF18	8995	1.06	1.36E-09	7.00E-07
Treatment & Medium: Day 1	ENSG00000135069	PSAT1	29968	0.81	1.46E-09	7.29E-07
Treatment & Medium: Day 1	ENSG00000143819	EPHX1	2052	-1.24	1.56E-09	7.57E-07
Treatment & Medium: Day 1	ENSG00000050344	NFE2L3	9603	1.19	1.68E-09	7.91E-07
Treatment & Medium: Day 1	ENSG00000168398	BDKRB2	624	-0.94	1.95E-09	8.87E-07
Treatment & Medium: Day 1	ENSG00000145777	TSLP	85480	-0.67	2.13E-09	9.45E-07
Treatment & Medium: Day 1	ENSG00000124766	SOX4	6659	-1.53	2.61E-09	1.08E-06
Treatment & Medium: Day 1	ENSG00000185432	METTL7A	25840	0.53	2.64E-09	1.08E-06
Treatment & Medium: Day 1	ENSG00000154864	PIEZO2	63895	-1.21	2.77E-09	1.08E-06
Treatment & Medium: Day 1	ENSG00000013293	SLC7A14	57709	-2.71	2.78E-09	1.08E-06
Treatment & Medium: Day 1	ENSG00000120129	DUSP1	1843	1.15	2.80E-09	1.08E-06
Treatment & Medium: Day 1	ENSG00000189221	MAOA	4128	0.93	2.96E-09	1.12E-06
Treatment & Medium: Day 1	ENSG00000141052	MYOCD	93649	1.86	3.06E-09	1.13E-06
Treatment & Medium: Day 1	ENSG00000136010	ALDH1L2	160428	0.00	3.14E-09	1.13E-06
Treatment & Medium: Day 1	ENSG00000135821	GLUL	2752	0.41	3.23E-09	1.14E-06
Treatment & Medium: Day 1	ENSG00000172197	MBOAT1	154141	-0.57	3.80E-09	1.31E-06
Treatment & Medium: Day 1	ENSG00000085117	CD82	3732	1.04	4.04E-09	1.36E-06

Treatment & Medium: Day 1	ENSG00000133110	POSTN	10631	-2.67	4.22E-09	1.38E-06
Treatment & Medium: Day 1	ENSG00000159674	SPON2	10417	-2.04	4.28E-09	1.38E-06
Treatment & Medium: Day 1	ENSG00000140285	FGF7	2252	-0.59	4.42E-09	1.40E-06
Treatment & Medium: Day 1	ENSG00000163734	CXCL3	2921	1.73	4.80E-09	1.46E-06
Treatment & Medium: Day 1	ENSG00000143341	HMCN1	83872	-2.28	4.82E-09	1.46E-06
Treatment & Medium: Day 1	ENSG00000087842	PIR	8544	-0.88	5.93E-09	1.75E-06
Treatment & Medium: Day 1	ENSG00000231924	PSG1	5669	-1.66	6.10E-09	1.75E-06
Treatment & Medium: Day 1	ENSG00000081041	CXCL2	2920	2.00	6.11E-09	1.75E-06
Treatment & Medium: Day 1	ENSG00000165272	AQP3	360	-2.12	6.83E-09	1.92E-06
Treatment & Medium: Day 1	ENSG00000173432	SAA1	6288	2.61	7.35E-09	2.00E-06
Treatment & Medium: Day 1	ENSG00000163347	CLDN1	9076	1.95	7.38E-09	2.00E-06
Treatment & Medium: Day 1	ENSG00000161570	CCL5	6352	2.90	7.48E-09	2.00E-06
Treatment & Medium: Day 1	ENSG00000110031	LPXN	9404	0.75	8.13E-09	2.13E-06
Treatment & Medium: Day 1	ENSG00000134321	RSAD2	91543	0.61	8.43E-09	2.14E-06
Treatment & Medium: Day 1	ENSG00000173376	NDNF	79625	-2.01	8.56E-09	2.14E-06
Treatment & Medium: Day 1	ENSG00000087086	FTL	2512	-1.34	8.56E-09	2.14E-06
Treatment & Medium: Day 1	ENSG00000140961	OSGIN1	29948	-0.91	9.37E-09	2.25E-06
Treatment & Medium: Day 1	ENSG00000127951	FGL2	10875	-1.70	9.41E-09	2.25E-06
Treatment & Medium: Day 1	ENSG00000019582	CD74	972	1.01	9.44E-09	2.25E-06
Treatment & Medium: Day 1	ENSG00000164307	ERAP1	51752	0.23	1.01E-08	2.36E-06
Treatment & Medium: Day 1	ENSG00000143127	ITGA10	8515	2.96	1.12E-08	2.58E-06
Treatment & Medium: Day 1	ENSG00000149571	KIRREL3	84623	0.45	1.13E-08	2.58E-06
Treatment & Medium: Day 1	ENSG00000196139	AKR1C3	8644	-1.31	1.15E-08	2.59E-06

Treatment & Medium: Day 1	ENSG00000138449	SLC40A1	30061	-1.61	1.20E-08	2.64E-06
Treatment & Medium: Day 1	ENSG00000196517	SLC6A9	6536	-0.59	1.21E-08	2.64E-06
Treatment & Medium: Day 1	ENSG00000120498	TEX11	56159	3.11	1.29E-08	2.77E-06
Treatment & Medium: Day 1	ENSG00000125148	MT2A	4502	1.56	1.31E-08	2.77E-06
Treatment & Medium: Day 1	ENSG00000065328	MCM10	55388	1.00	1.32E-08	2.77E-06
Treatment & Medium: Day 1	ENSG00000105894	PTN	5764	-1.01	1.34E-08	2.77E-06
Treatment & Medium: Day 1	ENSG00000170891	CYTL1	54360	-1.66	1.37E-08	2.79E-06
Treatment & Medium: Day 1	ENSG00000169239	CA5B	11238	-0.84	1.39E-08	2.79E-06
Treatment & Medium: Day 1	ENSG00000128342	LIF	3976	0.18	1.43E-08	2.84E-06
Treatment & Medium: Day 1	ENSG00000184374	COLEC10	10584	-0.76	1.47E-08	2.88E-06
Treatment & Medium: Day 1	ENSG00000136997	MYC	4609	1.12	1.49E-08	2.88E-06
Treatment & Medium: Day 1	ENSG00000164379	FOXQ1	94234	-1.53	1.56E-08	2.98E-06
Treatment & Medium: Day 1	ENSG00000196611	MMP1	4312	0.04	1.62E-08	3.07E-06
Treatment & Medium: Day 1	ENSG00000152952	PLOD2	5352	0.22	1.64E-08	3.07E-06
Treatment & Medium: Day 1	ENSG00000197646	PDCD1LG2	80380	0.50	1.69E-08	3.08E-06
Treatment & Medium: Day 1	ENSG00000181634	TNFSF15	9966	1.42	1.69E-08	3.08E-06
Treatment & Medium: Day 1	ENSG00000136048	DRAM1	55332	0.25	1.71E-08	3.08E-06
Treatment & Medium: Day 1	ENSG00000108688	CCL7	6354	0.85	1.74E-08	3.08E-06
Treatment & Medium: Day 1	ENSG00000154654	NCAM2	4685	-1.54	1.75E-08	3.08E-06
Treatment & Medium: Day 1	ENSG00000146411	SLC2A12	154091	-2.02	1.82E-08	3.17E-06
Treatment & Medium: Day 1	ENSG00000150630	VEGFC	7424	0.29	1.91E-08	3.24E-06
Treatment & Medium: Day 1	ENSG00000113361	CDH6	1004	-1.30	1.92E-08	3.24E-06
Treatment & Medium: Day 1	ENSG00000205364	MT1M	4499	1.62	1.92E-08	3.24E-06

Treatment & Medium: Day 1	ENSG00000115461	IGFBP5	3488	-1.84	1.95E-08	3.25E-06
Treatment & Medium: Day 1	ENSG00000101265	RASSF2	9770	-1.43	1.99E-08	3.26E-06
Treatment & Medium: Day 1	ENSG00000164309	CMYA5	202333	-1.57	2.00E-08	3.26E-06
Treatment & Medium: Day 1	ENSG00000104321	TRPA1	8989	2.02	2.03E-08	3.28E-06
Treatment & Medium: Day 1	ENSG00000166920	C15orf48	84419	1.07	2.18E-08	3.46E-06
Treatment & Medium: Day 1	ENSG00000136859	ANGPTL2	23452	-1.79	2.19E-08	3.46E-06
Treatment & Medium: Day 1	ENSG00000072682	P4HA2	8974	-0.17	2.23E-08	3.49E-06
Treatment & Medium: Day 1	ENSG00000253368	TRNP1	388610	1.12	2.26E-08	3.50E-06
Treatment & Medium: Day 1	ENSG00000164619	BMPER	168667	-0.05	2.36E-08	3.61E-06
Treatment & Medium: Day 1	ENSG00000124875	CXCL6	6372	2.13	2.44E-08	3.71E-06
Treatment & Medium: Day 1	ENSG00000099860	GADD45B	4616	0.62	2.49E-08	3.74E-06
Treatment & Medium: Day 1	ENSG00000137310	TCF19	6941	0.59	2.52E-08	3.75E-06
Treatment & Medium: Day 1	ENSG00000023445	BIRC3	330	1.99	2.60E-08	3.84E-06
Treatment & Medium: Day 1	ENSG00000176692	FOXC2	2303	1.22	2.73E-08	3.92E-06
Treatment & Medium: Day 1	ENSG00000138385	SSB	6741	0.71	2.73E-08	3.92E-06
Treatment & Medium: Day 1	ENSG00000260549	MT1L	4500	1.47	2.74E-08	3.92E-06
Treatment & Medium: Day 1	ENSG00000166508	MCM7	4176	0.67	2.91E-08	4.09E-06
Treatment & Medium: Day 1	ENSG00000123610	TNFAIP6	7130	-0.79	2.93E-08	4.09E-06
Treatment & Medium: Day 1	ENSG00000168679	SLC16A4	9122	-1.07	2.93E-08	4.09E-06
Treatment & Medium: Day 1	ENSG00000108797	CNTNAP1	8506	0.36	3.00E-08	4.14E-06
Treatment & Medium: Day 1	ENSG00000010932	FMO1	2326	-3.90	3.08E-08	4.21E-06
Treatment & Medium: Day 1	ENSG00000130303	BST2	684	0.45	3.13E-08	4.21E-06
Treatment & Medium: Day 1	ENSG00000073111	MCM2	4171	0.71	3.13E-08	4.21E-06

Treatment & Medium: Day 1	ENSG00000115523	GNLY	10578	2.37	3.15E-08	4.21E-06
Treatment & Medium: Day 1	ENSG00000196878	LAMB3	3914	0.51	3.20E-08	4.23E-06
Treatment & Medium: Day 1	ENSG00000100297	MCM5	4174	0.62	3.24E-08	4.26E-06
Treatment & Medium: Day 1	ENSG00000169908	TM4SF1	4071	1.05	3.29E-08	4.29E-06
Treatment & Medium: Day 1	ENSG00000168209	DDIT4	54541	-0.11	3.73E-08	4.78E-06
Treatment & Medium: Day 1	ENSG00000182985	CADM1	23705	0.24	3.73E-08	4.78E-06
Treatment & Medium: Day 1	ENSG00000176046	NUPR1	26471	-0.95	4.07E-08	5.16E-06
Treatment & Medium: Day 1	ENSG00000176597	B3GNT5	84002	1.46	4.29E-08	5.40E-06
Treatment & Medium: Day 1	ENSG00000138166	DUSP5	1847	0.52	4.37E-08	5.40E-06
Treatment & Medium: Day 1	ENSG00000139209	SLC38A4	55089	-0.78	4.39E-08	5.40E-06
Treatment & Medium: Day 1	ENSG00000158716	DUSP23	54935	0.21	4.39E-08	5.40E-06
Treatment & Medium: Day 1	ENSG00000111670	GNPTAB	79158	0.08	4.77E-08	5.78E-06
Treatment & Medium: Day 1	ENSG00000118113	MMP8	4317	3.17	4.77E-08	5.78E-06
Treatment & Medium: Day 1	ENSG00000173918	C1QTNF1	114897	0.82	4.90E-08	5.88E-06
Treatment & Medium: Day 1	ENSG00000167670	CHAF1A	10036	0.66	4.99E-08	5.91E-06
Treatment & Medium: Day 1	ENSG00000108691	CCL2	6347	1.07	5.00E-08	5.91E-06
Treatment & Medium: Day 1	ENSG00000183762	KREMEN1	83999	-0.93	5.04E-08	5.91E-06
Treatment & Medium: Day 1	ENSG00000133216	EPHB2	2048	-1.31	5.07E-08	5.91E-06
Treatment & Medium: Day 1	ENSG00000174804	FZD4	8322	-0.72	5.15E-08	5.91E-06
Treatment & Medium: Day 1	ENSG00000130513	GDF15	9518	-1.01	5.18E-08	5.91E-06
Treatment & Medium: Day 1	ENSG00000187957	DNER	92737	2.03	5.19E-08	5.91E-06
Treatment & Medium: Day 1	ENSG00000115902	SLC1A4	6509	0.42	5.57E-08	6.30E-06
Treatment & Medium: Day 1	ENSG00000118503	TNFAIP3	7128	2.02	5.66E-08	6.36E-06

Treatment & Medium: Day 1	ENSG00000171867	PRNP	5621	-1.03	5.71E-08	6.36E-06
Treatment & Medium: Day 1	ENSG00000126215	XRCC3	7517	0.84	5.80E-08	6.42E-06
Treatment & Medium: Day 1	ENSG00000136244	IL6	3569	1.18	5.93E-08	6.51E-06
Treatment & Medium: Day 1	ENSG00000184985	SORCS2	57537	-0.16	6.00E-08	6.54E-06
Treatment & Medium: Day 1	ENSG00000113645	WWC1	23286	1.54	6.03E-08	6.54E-06
Treatment & Medium: Day 1	ENSG00000131370	SH3BP5	9467	-0.87	6.17E-08	6.63E-06
Treatment & Medium: Day 1	ENSG00000104765	BNIP3L	665	-0.87	6.25E-08	6.65E-06
Treatment & Medium: Day 1	ENSG00000197632	SERPINB2	5055	2.69	6.27E-08	6.65E-06
Treatment & Medium: Day 1	ENSG00000173114	LRRN3	54674	1.24	6.43E-08	6.73E-06
Treatment & Medium: Day 1	ENSG00000029153	ARNTL2	56938	0.59	6.46E-08	6.73E-06
Treatment & Medium: Day 1	ENSG00000172403	SYNPO2	171024	-0.25	6.47E-08	6.73E-06
Treatment & Medium: Day 1	ENSG00000187193	MT1X	4501	1.69	6.60E-08	6.79E-06
Treatment & Medium: Day 1	ENSG00000163739	CXCL1	2919	1.36	6.62E-08	6.79E-06
Treatment & Medium: Day 1	ENSG00000164171	ITGA2	3673	0.28	6.82E-08	6.91E-06
Treatment & Medium: Day 1	ENSG00000163820	FYCO1	79443	-0.80	6.83E-08	6.91E-06
Treatment & Medium: Day 1	ENSG00000118564	FBXL5	26234	-0.43	7.00E-08	7.04E-06
Treatment & Medium: Day 1	ENSG00000114098	ARMC8	25852	0.65	7.07E-08	7.06E-06
Treatment & Medium: Day 1	ENSG00000149968	MMP3	4314	0.91	7.17E-08	7.12E-06
Treatment & Medium: Day 1	ENSG00000120899	PTK2B	2185	-1.61	7.68E-08	7.53E-06
Treatment & Medium: Day 1	ENSG00000117643	MAN1C1	57134	-1.08	7.68E-08	7.53E-06
Treatment & Medium: Day 1	ENSG00000038295	TLL1	7092	-2.64	7.85E-08	7.63E-06
Treatment & Medium: Day 1	ENSG00000166448	TMEM130	222865	-1.81	7.88E-08	7.63E-06
Treatment & Medium: Day 1	ENSG00000180875	GREM2	64388	-1.22	8.19E-08	7.88E-06

Treatment & Medium: Day 1	ENSG00000232956	SNHG15	285958	0.90	8.28E-08	7.92E-06
Treatment & Medium: Day 1	ENSG00000079257	LXN	56925	-1.24	8.54E-08	8.10E-06
Treatment & Medium: Day 1	ENSG00000173852	DPY19L1	23333	-0.08	8.57E-08	8.10E-06
Treatment & Medium: Day 1	ENSG00000143320	CRABP2	1382	-1.57	8.80E-08	8.26E-06
Treatment & Medium: Day 1	ENSG00000093009	CDC45	8318	0.79	8.89E-08	8.29E-06
Treatment & Medium: Day 1	ENSG00000109084	TMEM97	27346	0.61	9.23E-08	8.55E-06
Treatment & Medium: Day 1	ENSG00000100889	PCK2	5106	0.24	9.27E-08	8.55E-06
Treatment & Medium: Day 1	ENSG00000155760	FZD7	8324	-0.72	9.53E-08	8.65E-06
Treatment & Medium: Day 1	ENSG00000145819	ARHGAP26	23092	-1.30	9.54E-08	8.65E-06
Treatment & Medium: Day 1	ENSG00000107562	CXCL12	6387	-2.19	9.57E-08	8.65E-06
Treatment & Medium: Day 1	ENSG00000095303	PTGS1	5742	0.89	9.61E-08	8.65E-06
Treatment & Medium: Day 1	ENSG00000179104	TMTC2	160335	-1.45	9.81E-08	8.70E-06
Treatment & Medium: Day 1	ENSG00000070081	NUCB2	4925	-0.06	9.82E-08	8.70E-06
Treatment & Medium: Day 1	ENSG00000125851	PCSK2	5126	-1.35	9.83E-08	8.70E-06
Treatment & Medium: Day 1	ENSG00000144218	AFF3	3899	-1.77	9.92E-08	8.73E-06
Treatment & Medium: Day 1	ENSG00000197208	SLC22A4	6583	0.74	1.01E-07	8.81E-06
Treatment & Medium: Day 1	ENSG00000156427	FGF18	8817	-1.80	1.04E-07	9.06E-06
Treatment & Medium: Day 1	ENSG00000152953	STK32B	55351	-1.05	1.05E-07	9.06E-06
Treatment & Medium: Day 1	ENSG00000141150	RASL10B	91608	0.16	1.06E-07	9.06E-06
Treatment & Medium: Day 1	ENSG00000004399	PLXND1	23129	-0.80	1.06E-07	9.06E-06
Treatment & Medium: Day 1	ENSG00000198805	PNP	4860	0.97	1.10E-07	9.33E-06
Treatment & Medium: Day 1	ENSG00000106780	MEGF9	1955	-0.86	1.11E-07	9.37E-06
Treatment & Medium: Day 1	ENSG00000171509	RXFP1	59350	-1.22	1.12E-07	9.41E-06

Treatment & Medium: Day 1	ENSG00000158258	CLSTN2	64084	-0.68	1.14E-07	9.53E-06
Treatment & Medium: Day 1	ENSG00000145244	CORIN	10699	1.97	1.15E-07	9.61E-06
Treatment & Medium: Day 1	ENSG00000141404	GNAL	2774	-1.73	1.18E-07	9.81E-06
Treatment & Medium: Day 1	ENSG00000103710	RASL12	51285	-1.83	1.20E-07	9.86E-06
Treatment & Medium: Day 1	ENSG00000085662	AKR1B1	231	0.73	1.21E-07	9.95E-06
Treatment & Medium: Day 1	ENSG00000008517	IL32	9235	2.82	1.24E-07	1.01E-05
Treatment & Medium: Day 1	ENSG00000234745	HLA-B	3106	0.51	1.29E-07	1.04E-05
Treatment & Medium: Day 1	ENSG00000131153	GINS2	51659	0.82	1.29E-07	1.04E-05
Treatment & Medium: Day 1	ENSG00000119138	KLF9	687	-0.21	1.30E-07	1.05E-05
Treatment & Medium: Day 1	ENSG00000178921	PFAS	5198	0.72	1.31E-07	1.05E-05
Treatment & Medium: Day 1	ENSG00000116991	SIPA1L2	57568	-1.08	1.33E-07	1.06E-05
Treatment & Medium: Day 1	ENSG00000092969	TGFB2	7042	-2.10	1.39E-07	1.10E-05
Treatment & Medium: Day 1	ENSG00000166592	RRAD	6236	0.14	1.39E-07	1.10E-05
Treatment & Medium: Day 1	ENSG00000135373	EHF	26298	3.20	1.41E-07	1.10E-05
Treatment & Medium: Day 1	ENSG00000188157	AGRN	375790	0.06	1.43E-07	1.11E-05
Treatment & Medium: Day 1	ENSG00000225783	MIAT	440823	-2.89	1.44E-07	1.11E-05
Treatment & Medium: Day 1	ENSG00000141140	MYO19	80179	0.69	1.44E-07	1.11E-05
Treatment & Medium: Day 1	ENSG00000072210	ALDH3A2	224	-1.24	1.46E-07	1.12E-05
Treatment & Medium: Day 1	ENSG00000168955	TM4SF20	79853	-1.63	1.48E-07	1.13E-05
Treatment & Medium: Day 1	ENSG00000006062	MAP3K14	9020	-0.83	1.48E-07	1.13E-05
Treatment & Medium: Day 1	ENSG00000116191	RALGPS2	55103	-0.60	1.51E-07	1.14E-05
Treatment & Medium: Day 1	ENSG00000134201	GSTM5	2949	-0.87	1.53E-07	1.15E-05
Treatment & Medium: Day 1	ENSG00000144369	FAM171B	165215	-1.32	1.54E-07	1.15E-05

Treatment & Medium: Day 1	ENSG00000170381	SEMA3E	9723	-1.34	1.58E-07	1.18E-05
Treatment & Medium: Day 1	ENSG00000085840	ORC1	4998	0.87	1.64E-07	1.22E-05
Treatment & Medium: Day 1	ENSG00000113657	DPYSL3	1809	0.03	1.68E-07	1.24E-05
Treatment & Medium: Day 1	ENSG00000165092	ALDH1A1	216	-2.59	1.75E-07	1.28E-05
Treatment & Medium: Day 1	ENSG00000170214	ADRA1B	147	0.52	1.78E-07	1.30E-05
Treatment & Medium: Day 1	ENSG00000116285	ERRFI1	54206	-0.73	1.80E-07	1.31E-05
Treatment & Medium: Day 1	ENSG00000115828	QPCT	25797	-0.01	1.84E-07	1.34E-05
Treatment & Medium: Day 1	ENSG00000152377	SPOCK1	6695	-0.96	1.88E-07	1.35E-05
Treatment & Medium: Day 1	ENSG00000112118	MCM3	4172	0.74	1.89E-07	1.35E-05
Treatment & Medium: Day 1	ENSG00000105499	PLA2G4C	8605	0.15	1.89E-07	1.35E-05
Treatment & Medium: Day 1	ENSG00000122035	RASL11A	387496	-0.43	1.93E-07	1.37E-05
Treatment & Medium: Day 1	ENSG00000233452	STXBP5-AS1	729178	0.24	1.96E-07	1.39E-05
Treatment & Medium: Day 1	ENSG00000013297	CLDN11	5010	-1.10	1.99E-07	1.40E-05
Treatment & Medium: Day 1	ENSG00000135424	ITGA7	3679	-0.68	2.00E-07	1.40E-05
Treatment & Medium: Day 1	ENSG00000241399	CD302	9936	-0.64	2.07E-07	1.45E-05
Treatment & Medium: Day 1	ENSG00000172348	RCAN2	10231	-1.57	2.09E-07	1.45E-05
Treatment & Medium: Day 1	ENSG00000166963	MAP1A	4130	-1.17	2.10E-07	1.46E-05
Treatment & Medium: Day 1	ENSG00000073756	PTGS2	5743	-1.21	2.16E-07	1.49E-05
Treatment & Medium: Day 1	ENSG00000226950	DANCR	57291	0.63	2.22E-07	1.52E-05
Treatment & Medium: Day 1	ENSG00000196549	MME	4311	0.28	2.27E-07	1.55E-05
Treatment & Medium: Day 1	ENSG00000132170	PPARG	5468	0.03	2.29E-07	1.56E-05
Treatment & Medium: Day 1	ENSG00000152463	OLAH	55301	2.35	2.38E-07	1.61E-05
Treatment & Medium: Day 1	ENSG00000003989	SLC7A2	6542	3.09	2.43E-07	1.64E-05

Treatment & Medium: Day 1	ENSG00000142657	PGD	5226	-0.74	2.45E-07	1.64E-05
Treatment & Medium: Day 1	ENSG00000048162	NOP16	51491	0.75	2.48E-07	1.65E-05
Treatment & Medium: Day 1	ENSG00000135114	OASL	8638	1.17	2.50E-07	1.66E-05
Treatment & Medium: Day 1	ENSG00000100504	PYGL	5836	-0.45	2.51E-07	1.66E-05
Treatment & Medium: Day 1	ENSG00000155511	GRIA1	2890	0.21	2.53E-07	1.67E-05
Treatment & Medium: Day 1	ENSG00000183486	MX2	4600	0.35	2.59E-07	1.69E-05
Treatment & Medium: Day 1	ENSG00000085999	RAD54L	8438	0.65	2.59E-07	1.69E-05
Treatment & Medium: Day 1	ENSG00000164220	F2RL2	2151	-1.08	2.60E-07	1.69E-05
Treatment & Medium: Day 1	ENSG00000242125	SNHG3	8420	0.82	2.60E-07	1.69E-05
Treatment & Medium: Day 1	ENSG00000241749	RPSAP52	204010	1.33	2.65E-07	1.71E-05
Treatment & Medium: Day 1	ENSG00000134326	CMPK2	129607	0.59	2.68E-07	1.72E-05
Treatment & Medium: Day 1	ENSG00000122641	INHBA	3624	0.34	2.69E-07	1.72E-05
Treatment & Medium: Day 1	ENSG00000176974	SHMT1	6470	0.31	2.73E-07	1.73E-05
Treatment & Medium: Day 1	ENSG00000204634	TBC1D8	11138	0.68	2.73E-07	1.73E-05
Treatment & Medium: Day 1	ENSG00000092853	CLSPN	63967	0.72	2.75E-07	1.74E-05
Treatment & Medium: Day 1	ENSG00000183578	TNFAIP8L3	388121	0.10	2.76E-07	1.74E-05
Treatment & Medium: Day 1	ENSG00000075223	SEMA3C	10512	0.31	2.80E-07	1.76E-05
Treatment & Medium: Day 1	ENSG00000172061	LRRC15	131578	-0.47	2.85E-07	1.78E-05
Treatment & Medium: Day 1	ENSG00000131389	SLC6A6	6533	-0.59	2.88E-07	1.79E-05
Treatment & Medium: Day 1	ENSG00000065534	MYLK	4638	-0.11	2.93E-07	1.81E-05
Treatment & Medium: Day 1	ENSG00000173638	SLC19A1	6573	0.94	2.98E-07	1.84E-05
Treatment & Medium: Day 1	ENSG00000149948	HMGA2	8091	1.05	2.99E-07	1.84E-05
Treatment & Medium: Day 1	ENSG00000171848	RRM2	6241	0.73	3.01E-07	1.84E-05

Treatment & Medium: Day 1	ENSG00000160957	RECQL4	9401	0.51	3.08E-07	1.88E-05
Treatment & Medium: Day 1	ENSG00000138074	SLC5A6	8884	0.60	3.09E-07	1.88E-05
Treatment & Medium: Day 1	ENSG00000162073	PAQR4	124222	0.80	3.20E-07	1.93E-05
Treatment & Medium: Day 1	ENSG00000104738	MCM4	4173	0.59	3.22E-07	1.94E-05
Treatment & Medium: Day 1	ENSG00000162616	DNAJB4	11080	0.92	3.23E-07	1.94E-05
Treatment & Medium: Day 1	ENSG00000120658	ENOX1	55068	-1.20	3.25E-07	1.94E-05
Treatment & Medium: Day 1	ENSG00000123405	NFE2	4778	-2.12	3.29E-07	1.96E-05
Treatment & Medium: Day 1	ENSG00000132000	PODNL1	79883	-1.36	3.31E-07	1.96E-05
Treatment & Medium: Day 1	ENSG00000167513	CDT1	81620	0.73	3.34E-07	1.98E-05
Treatment & Medium: Day 1	ENSG00000184588	PDE4B	5142	-0.71	3.40E-07	2.00E-05
Treatment & Medium: Day 1	ENSG00000172986	GXYLT2	727936	-1.17	3.41E-07	2.00E-05
Treatment & Medium: Day 1	ENSG00000108448	TRIM16L	147166	-0.84	3.42E-07	2.00E-05
Treatment & Medium: Day 1	ENSG00000176658	MYO1D	4642	-1.21	3.44E-07	2.00E-05
Treatment & Medium: Day 1	ENSG00000071575	TRIB2	28951	-0.65	3.45E-07	2.00E-05
Treatment & Medium: Day 1	ENSG00000135362	PRR5L	79899	0.80	3.45E-07	2.00E-05
Treatment & Medium: Day 1	ENSG00000119630	PGF	5228	-1.63	3.47E-07	2.00E-05
Treatment & Medium: Day 1	ENSG00000144959	NCEH1	57552	0.83	3.48E-07	2.00E-05
Treatment & Medium: Day 1	ENSG00000164920	OSR2	116039	-1.50	3.50E-07	2.00E-05
Treatment & Medium: Day 1	ENSG00000055163	CYFIP2	26999	0.47	3.60E-07	2.05E-05
Treatment & Medium: Day 1	ENSG00000007968	E2F2	1870	0.84	3.66E-07	2.08E-05
Treatment & Medium: Day 1	ENSG00000157150	TIMP4	7079	0.45	3.73E-07	2.11E-05
Treatment & Medium: Day 1	ENSG00000196932	TMEM26	219623	-2.12	3.79E-07	2.13E-05
Treatment & Medium: Day 1	ENSG00000125538	IL1B	3553	2.66	3.80E-07	2.13E-05

Treatment & Medium: Day 1	ENSG00000109906	ZBTB16	7704	8.67	3.85E-07	2.16E-05
Treatment & Medium: Day 1	ENSG00000148175	STOM	2040	-0.84	3.94E-07	2.20E-05
Treatment & Medium: Day 1	ENSG00000157514	TSC22D3	1831	0.59	4.02E-07	2.23E-05
Treatment & Medium: Day 1	ENSG00000138646	HERC5	51191	0.76	4.04E-07	2.23E-05
Treatment & Medium: Day 1	ENSG00000122862	SRGN	5552	0.83	4.06E-07	2.24E-05
Treatment & Medium: Day 1	ENSG00000090376	IRAK3	11213	0.84	4.22E-07	2.32E-05
Treatment & Medium: Day 1	ENSG00000123374	CDK2	1017	0.54	4.27E-07	2.34E-05
Treatment & Medium: Day 1	ENSG00000168496	FEN1	2237	0.64	4.40E-07	2.40E-05
Treatment & Medium: Day 1	ENSG00000174938	SEZ6L2	26470	-0.29	4.45E-07	2.42E-05
Treatment & Medium: Day 1	ENSG00000157368	IL34	146433	0.59	4.50E-07	2.44E-05
Treatment & Medium: Day 1	ENSG00000105245	NUMBL	9253	-0.66	4.58E-07	2.47E-05
Treatment & Medium: Day 1	ENSG00000123243	ITIH5	80760	0.35	4.61E-07	2.47E-05
Treatment & Medium: Day 1	ENSG00000197506	SLC28A3	64078	1.50	4.61E-07	2.47E-05
Treatment & Medium: Day 1	ENSG00000123136	DDX39A	10212	0.58	4.65E-07	2.49E-05
Treatment & Medium: Day 1	ENSG00000128016	ZFP36	7538	-0.29	4.81E-07	2.56E-05
Treatment & Medium: Day 1	ENSG00000152217	SETBP1	26040	-1.53	4.86E-07	2.58E-05
Treatment & Medium: Day 1	ENSG00000105855	ITGB8	3696	-1.78	4.97E-07	2.62E-05
Treatment & Medium: Day 1	ENSG00000113739	STC2	8614	0.85	4.99E-07	2.62E-05
Treatment & Medium: Day 1	ENSG00000135074	ADAM19	8728	-0.91	5.00E-07	2.62E-05
Treatment & Medium: Day 1	ENSG00000108960	MMD	23531	0.56	5.01E-07	2.62E-05
Treatment & Medium: Day 1	ENSG00000112699	GMDS	2762	-0.55	5.03E-07	2.63E-05
Treatment & Medium: Day 1	ENSG00000065882	TBC1D1	23216	0.32	5.10E-07	2.65E-05
Treatment & Medium: Day 1	ENSG00000182197	EXT1	2131	-0.54	5.12E-07	2.65E-05

Treatment & Medium: Day 1	ENSG00000166923	GREM1	26585	-0.71	5.14E-07	2.65E-05
Treatment & Medium: Day 1	ENSG00000147872	PLIN2	123	0.22	5.21E-07	2.68E-05
Treatment & Medium: Day 1	ENSG00000181019	NQO1	1728	-0.73	5.30E-07	2.72E-05
Treatment & Medium: Day 1	ENSG00000154175	ABI3BP	25890	0.96	5.36E-07	2.74E-05
Treatment & Medium: Day 1	ENSG00000164292	RHOBTB3	22836	-0.81	5.48E-07	2.79E-05
Treatment & Medium: Day 1	ENSG00000099998	GGT5	2687	2.02	5.54E-07	2.81E-05
Treatment & Medium: Day 1	ENSG00000145632	PLK2	10769	-0.88	5.63E-07	2.85E-05
Treatment & Medium: Day 1	ENSG00000079931	MOXD1	26002	0.15	5.73E-07	2.89E-05
Treatment & Medium: Day 1	ENSG00000134198	TSPAN2	10100	-1.11	5.79E-07	2.91E-05
Treatment & Medium: Day 1	ENSG00000164484	TMEM200A	114801	-1.11	5.83E-07	2.92E-05
Treatment & Medium: Day 1	ENSG00000115963	RND3	390	0.00	6.06E-07	3.03E-05
Treatment & Medium: Day 1	ENSG00000187955	COL14A1	7373	-1.53	6.13E-07	3.05E-05
Treatment & Medium: Day 1	ENSG00000101412	E2F1	1869	0.68	6.24E-07	3.10E-05
Treatment & Medium: Day 1	ENSG00000106366	SERPINE1	5054	0.94	6.28E-07	3.11E-05
Treatment & Medium: Day 1	ENSG00000197415	VEPH1	79674	0.30	6.45E-07	3.18E-05
Treatment & Medium: Day 1	ENSG00000145604	SKP2	6502	-0.04	6.49E-07	3.18E-05
Treatment & Medium: Day 1	ENSG00000138131	LOXL4	84171	-0.55	6.50E-07	3.18E-05
Treatment & Medium: Day 1	ENSG00000231925	TAPBP	6892	0.38	6.51E-07	3.18E-05
Treatment & Medium: Day 1	ENSG00000107984	DKK1	22943	0.81	6.55E-07	3.19E-05
Treatment & Medium: Day 1	ENSG00000071539	TRIP13	9319	0.66	6.58E-07	3.20E-05
Treatment & Medium: Day 1	ENSG00000104081	BMF	90427	-2.54	6.75E-07	3.27E-05
Treatment & Medium: Day 1	ENSG00000140945	CDH13	1012	0.08	6.77E-07	3.27E-05
Treatment & Medium: Day 1	ENSG00000164929	BAALC	79870	-1.63	6.92E-07	3.33E-05

Treatment & Medium: Day 1	ENSG00000087494	PTHLH	5744	-1.87	7.00E-07	3.35E-05
Treatment & Medium: Day 1	ENSG00000099901	RANBP1	5902	0.63	7.01E-07	3.35E-05
Treatment & Medium: Day 1	ENSG00000221869	CEBPD	1052	-0.37	7.13E-07	3.40E-05
Treatment & Medium: Day 1	ENSG00000133026	MYH10	4628	-0.56	7.16E-07	3.40E-05
Treatment & Medium: Day 1	ENSG00000185070	FLRT2	23768	-0.65	7.21E-07	3.42E-05
Treatment & Medium: Day 1	ENSG00000005238	FAM214B	80256	-0.09	7.29E-07	3.44E-05
Treatment & Medium: Day 1	ENSG00000127564	PKMYT1	9088	0.62	7.39E-07	3.48E-05
Treatment & Medium: Day 1	ENSG00000158467	AHCYL2	23382	-0.98	7.48E-07	3.51E-05
Treatment & Medium: Day 1	ENSG00000171451	DSEL	92126	-0.61	7.54E-07	3.53E-05
Treatment & Medium: Day 1	ENSG00000164776	PHKG1	5260	-2.59	7.66E-07	3.58E-05
Treatment & Medium: Day 1	ENSG00000139112	GABARAPL1	23710	-1.01	7.71E-07	3.59E-05
Treatment & Medium: Day 1	ENSG00000014138	POLA2	23649	0.61	7.84E-07	3.64E-05
Treatment & Medium: Day 1	ENSG00000122547	EEPD1	80820	-1.33	7.92E-07	3.66E-05
Treatment & Medium: Day 1	ENSG00000198542	ITGBL1	9358	-0.35	8.11E-07	3.74E-05
Treatment & Medium: Day 1	ENSG00000132603	NIP7	51388	0.65	8.13E-07	3.74E-05
Treatment & Medium: Day 1	ENSG00000172915	NBEA	26960	-0.95	8.29E-07	3.80E-05
Treatment & Medium: Day 1	ENSG00000152402	GUCY1A2	2977	-1.22	8.35E-07	3.82E-05
Treatment & Medium: Day 1	ENSG00000136960	ENPP2	5168	-0.75	8.49E-07	3.87E-05
Treatment & Medium: Day 1	ENSG00000114796	KLHL24	54800	-1.05	8.57E-07	3.88E-05
Treatment & Medium: Day 1	ENSG00000106123	EPHB6	2051	-0.31	8.57E-07	3.88E-05
Treatment & Medium: Day 1	ENSG00000106976	DNM1	1759	-1.23	8.73E-07	3.94E-05
Treatment & Medium: Day 1	ENSG00000185920	PTCH1	5727	-1.27	8.84E-07	3.98E-05
Treatment & Medium: Day 1	ENSG00000211445	GPX3	2878	-0.90	8.89E-07	3.98E-05

Treatment & Medium: Day 1	ENSG00000174697	LEP	3952	0.41	8.90E-07	3.98E-05
Treatment & Medium: Day 1	ENSG00000135919	SERPINE2	5270	-0.27	8.95E-07	3.99E-05
Treatment & Medium: Day 1	ENSG00000179431	FJX1	24147	0.83	8.99E-07	4.00E-05
Treatment & Medium: Day 1	ENSG00000146233	CYP39A1	51302	-0.98	9.02E-07	4.00E-05
Treatment & Medium: Day 1	ENSG00000182667	NTM	50863	0.24	9.03E-07	4.00E-05
Treatment & Medium: Day 1	ENSG00000128590	DNAJB9	4189	-0.12	9.10E-07	4.02E-05
Treatment & Medium: Day 1	ENSG00000137501	SYTL2	54843	-0.89	9.15E-07	4.03E-05
Treatment & Medium: Day 1	ENSG00000124749	COL21A1	81578	-2.08	9.20E-07	4.03E-05
Treatment & Medium: Day 1	ENSG00000089127	OAS1	4938	0.91	9.20E-07	4.03E-05
Treatment & Medium: Day 1	ENSG00000154589	LY96	23643	-0.21	9.25E-07	4.04E-05
Treatment & Medium: Day 1	ENSG00000094963	FMO2	2327	-3.05	9.48E-07	4.13E-05
Treatment & Medium: Day 1	ENSG00000100219	XBP1	7494	0.17	9.51E-07	4.13E-05
Treatment & Medium: Day 1	ENSG00000109072	VTN	7448	-1.00	9.54E-07	4.13E-05
Treatment & Medium: Day 1	ENSG00000175538	KCNE3	10008	-1.78	9.81E-07	4.23E-05
Treatment & Medium: Day 1	ENSG00000127241	MASP1	5648	0.05	9.82E-07	4.23E-05
Treatment & Medium: Day 1	ENSG00000104611	SH2D4A	63898	-0.74	9.85E-07	4.23E-05
Treatment & Medium: Day 1	ENSG00000143476	DTL	51514	0.84	9.87E-07	4.23E-05
Treatment & Medium: Day 1	ENSG00000166710	B2M	567	0.26	9.92E-07	4.23E-05
Treatment & Medium: Day 1	ENSG00000176890	TYMS	7298	0.61	9.99E-07	4.25E-05
Treatment & Medium: Day 1	ENSG00000102057	KCND1	3750	-0.20	1.01E-06	4.27E-05
Treatment & Medium: Day 1	ENSG00000060718	COL11A1	1301	-1.22	1.03E-06	4.35E-05
Treatment & Medium: Day 1	ENSG00000143799	PARP1	142	0.48	1.05E-06	4.41E-05
Treatment & Medium: Day 1	ENSG00000162595	DIRAS3	9077	-2.18	1.06E-06	4.45E-05

Treatment & Medium: Day 1	ENSG00000171621	SPSB1	80176	-0.64	1.06E-06	4.47E-05
Treatment & Medium: Day 1	ENSG00000196526	AFAP1	60312	-0.05	1.07E-06	4.47E-05
Treatment & Medium: Day 1	ENSG00000163703	CRELD1	78987	-0.13	1.07E-06	4.47E-05
Treatment & Medium: Day 1	ENSG00000114251	WNT5A	7474	-0.47	1.08E-06	4.47E-05
Treatment & Medium: Day 1	ENSG00000137273	FOXF2	2295	-0.78	1.08E-06	4.47E-05
Treatment & Medium: Day 1	ENSG00000227051	C14orf132	56967	-0.80	1.09E-06	4.51E-05
Treatment & Medium: Day 1	ENSG00000188641	DPYD	1806	-0.47	1.09E-06	4.51E-05
Treatment & Medium: Day 1	ENSG00000071967	CYBRD1	79901	-0.87	1.10E-06	4.52E-05
Treatment & Medium: Day 1	ENSG00000174371	EXO1	9156	0.68	1.10E-06	4.54E-05
Treatment & Medium: Day 1	ENSG00000198910	L1CAM	3897	-0.60	1.12E-06	4.57E-05
Treatment & Medium: Day 1	ENSG00000175899	A2M	2	-1.55	1.16E-06	4.72E-05
Treatment & Medium: Day 1	ENSG00000188483	IER5L	389792	-1.14	1.16E-06	4.74E-05
Treatment & Medium: Day 1	ENSG00000077232	DNAJC10	54431	0.37	1.18E-06	4.79E-05
Treatment & Medium: Day 1	ENSG00000183287	CCBE1	147372	0.71	1.18E-06	4.79E-05
Treatment & Medium: Day 1	ENSG00000241644	INMT	11185	-1.14	1.21E-06	4.89E-05
Treatment & Medium: Day 1	ENSG00000108639	SYNGR2	9144	0.39	1.21E-06	4.89E-05
Treatment & Medium: Day 1	ENSG00000146232	NFKBIE	4794	0.97	1.23E-06	4.94E-05
Treatment & Medium: Day 1	ENSG00000077150	NFKB2	4791	0.71	1.25E-06	5.01E-05
Treatment & Medium: Day 1	ENSG00000159259	CHAF1B	8208	0.60	1.25E-06	5.01E-05
Treatment & Medium: Day 1	ENSG00000103381	CPPED1	55313	0.48	1.26E-06	5.02E-05
Treatment & Medium: Day 1	ENSG00000123700	KCNJ2	3759	-0.88	1.26E-06	5.02E-05
Treatment & Medium: Day 1	ENSG00000171793	CTPS1	1503	0.71	1.26E-06	5.02E-05
Treatment & Medium: Day 1	ENSG00000104332	SFRP1	6422	-1.33	1.29E-06	5.09E-05

Treatment & Medium: Day 1	ENSG00000178573	MAF	4094	-2.39	1.29E-06	5.12E-05
Treatment & Medium: Day 1	ENSG00000134070	IRAK2	3656	0.70	1.30E-06	5.14E-05
Treatment & Medium: Day 1	ENSG00000170779	CDCA4	55038	0.60	1.32E-06	5.18E-05
Treatment & Medium: Day 1	ENSG00000184254	ALDH1A3	220	-0.13	1.33E-06	5.23E-05
Treatment & Medium: Day 1	ENSG00000089685	BIRC5	332	0.47	1.35E-06	5.26E-05
Treatment & Medium: Day 1	ENSG00000105185	PDCD5	9141	0.75	1.35E-06	5.28E-05
Treatment & Medium: Day 1	ENSG00000177192	PUS1	80324	0.55	1.38E-06	5.38E-05
Treatment & Medium: Day 1	ENSG00000235162	C12orf75	387882	0.47	1.39E-06	5.41E-05
Treatment & Medium: Day 1	ENSG00000146670	CDCA5	113130	0.61	1.41E-06	5.45E-05
Treatment & Medium: Day 1	ENSG00000132561	MATN2	4147	-0.75	1.42E-06	5.49E-05
Treatment & Medium: Day 1	ENSG00000100714	MTHFD1	4522	0.52	1.43E-06	5.52E-05
Treatment & Medium: Day 1	ENSG00000184922	FMNL1	752	-0.20	1.45E-06	5.59E-05
Treatment & Medium: Day 1	ENSG00000103257	SLC7A5	8140	0.47	1.50E-06	5.75E-05
Treatment & Medium: Day 1	ENSG00000169245	CXCL10	3627	3.63	1.53E-06	5.86E-05
Treatment & Medium: Day 1	ENSG00000157601	MX1	4599	0.73	1.54E-06	5.86E-05
Treatment & Medium: Day 1	ENSG00000169744	LDB2	9079	-1.30	1.55E-06	5.88E-05
Treatment & Medium: Day 1	ENSG00000092470	WDR76	79968	0.74	1.57E-06	5.94E-05
Treatment & Medium: Day 1	ENSG00000144749	LRIG1	26018	-0.16	1.57E-06	5.94E-05
Treatment & Medium: Day 1	ENSG00000174136	RGMB	285704	-0.17	1.59E-06	6.01E-05
Treatment & Medium: Day 1	ENSG00000034063	UHRF1	29128	0.56	1.60E-06	6.05E-05
Treatment & Medium: Day 1	ENSG00000105856	HBP1	26959	-0.68	1.62E-06	6.07E-05
Treatment & Medium: Day 1	ENSG00000165655	ZNF503	84858	-1.35	1.62E-06	6.07E-05
Treatment & Medium: Day 1	ENSG00000182199	SHMT2	6472	0.25	1.62E-06	6.07E-05

Treatment & Medium: Day 1	ENSG00000164761	TNFRSF11B	4982	-0.60	1.64E-06	6.12E-05
Treatment & Medium: Day 1	ENSG00000156011	PSD3	23362	-0.89	1.66E-06	6.18E-05
Treatment & Medium: Day 1	ENSG00000119714	GPR68	8111	0.84	1.69E-06	6.26E-05
Treatment & Medium: Day 1	ENSG00000179862	CITED4	163732	0.93	1.71E-06	6.34E-05
Treatment & Medium: Day 1	ENSG00000139132	FGD4	121512	0.64	1.72E-06	6.35E-05
Treatment & Medium: Day 1	ENSG00000132646	PCNA	5111	0.60	1.72E-06	6.35E-05
Treatment & Medium: Day 1	ENSG00000145911	N4BP3	23138	-1.34	1.73E-06	6.36E-05
Treatment & Medium: Day 1	ENSG00000145220	LYAR	55646	0.69	1.74E-06	6.38E-05
Treatment & Medium: Day 1	ENSG00000187134	AKR1C1	1645	-0.90	1.77E-06	6.47E-05
Treatment & Medium: Day 1	ENSG00000162692	VCAM1	7412	-0.75	1.78E-06	6.47E-05
Treatment & Medium: Day 1	ENSG00000136542	GALNT5	11227	-0.03	1.78E-06	6.47E-05
Treatment & Medium: Day 1	ENSG00000168393	DTYMK	1841	0.44	1.78E-06	6.47E-05
Treatment & Medium: Day 1	ENSG00000158714	SLAMF8	56833	3.96	1.80E-06	6.54E-05
Treatment & Medium: Day 1	ENSG00000100162	CENPM	79019	0.59	1.83E-06	6.59E-05
Treatment & Medium: Day 1	ENSG00000169122	FAM110B	90362	-0.14	1.83E-06	6.59E-05
Treatment & Medium: Day 1	ENSG00000104313	EYA1	2138	-1.26	1.83E-06	6.61E-05
Treatment & Medium: Day 1	ENSG00000131470	PSMC3IP	29893	0.79	1.86E-06	6.69E-05
Treatment & Medium: Day 1	ENSG00000165633	VSTM4	196740	-1.17	1.87E-06	6.69E-05
Treatment & Medium: Day 1	ENSG00000153071	DAB2	1601	-0.75	1.88E-06	6.69E-05
Treatment & Medium: Day 1	ENSG00000122707	RECK	8434	-0.85	1.88E-06	6.69E-05
Treatment & Medium: Day 1	ENSG00000164112	TMEM155	132332	-0.71	1.88E-06	6.69E-05
Treatment & Medium: Day 1	ENSG00000170271	FAXDC2	10826	-0.94	1.88E-06	6.69E-05
Treatment & Medium: Day 1	ENSG00000176697	BDNF	627	-0.83	1.89E-06	6.69E-05

Treatment & Medium: Day 1	ENSG00000062822	POLD1	5424	0.67	1.89E-06	6.70E-05
Treatment & Medium: Day 1	ENSG00000100911	PSME2	5721	0.59	1.90E-06	6.70E-05
Treatment & Medium: Day 1	ENSG00000166482	MFAP4	4239	-1.53	1.90E-06	6.70E-05
Treatment & Medium: Day 1	ENSG00000198612	COPS8	10920	0.55	1.92E-06	6.73E-05
Treatment & Medium: Day 1	ENSG00000172164	SNTB1	6641	-0.40	1.92E-06	6.73E-05
Treatment & Medium: Day 1	ENSG00000160712	IL6R	3570	-0.55	1.93E-06	6.73E-05
Treatment & Medium: Day 1	ENSG00000137809	ITGA11	22801	-0.95	1.94E-06	6.75E-05
Treatment & Medium: Day 1	ENSG00000105889	STEAP1B	256227	0.65	1.94E-06	6.75E-05
Treatment & Medium: Day 1	ENSG00000185745	IFIT1	3434	0.25	1.96E-06	6.78E-05
Treatment & Medium: Day 1	ENSG00000128965	CHAC1	79094	0.57	1.96E-06	6.78E-05
Treatment & Medium: Day 1	ENSG00000111799	COL12A1	1303	-0.92	1.97E-06	6.80E-05
Treatment & Medium: Day 1	ENSG00000122034	GTF3A	2971	0.60	1.99E-06	6.87E-05
Treatment & Medium: Day 1	ENSG00000135218	CD36	948	-1.39	2.00E-06	6.89E-05
Treatment & Medium: Day 1	ENSG00000136052	SLC41A2	84102	-0.27	2.02E-06	6.91E-05
Treatment & Medium: Day 1	ENSG00000166197	NOLC1	9221	0.69	2.02E-06	6.91E-05
Treatment & Medium: Day 1	ENSG00000166750	SLFN5	162394	-0.09	2.02E-06	6.91E-05
Treatment & Medium: Day 1	ENSG00000003436	TFPI	7035	0.26	2.04E-06	6.95E-05
Treatment & Medium: Day 1	ENSG00000116774	OLFML3	56944	-1.00	2.04E-06	6.95E-05
Treatment & Medium: Day 1	ENSG00000106772	PRUNE2	158471	-0.51	2.08E-06	7.08E-05
Treatment & Medium: Day 1	ENSG00000127586	CHTF18	63922	0.49	2.10E-06	7.10E-05
Treatment & Medium: Day 1	ENSG00000165480	SKA3	221150	0.70	2.10E-06	7.10E-05
Treatment & Medium: Day 1	ENSG00000145147	SLIT2	9353	0.14	2.11E-06	7.13E-05
Treatment & Medium: Day 1	ENSG00000149212	SESN3	143686	-1.73	2.14E-06	7.19E-05

Treatment & Medium: Day 1	ENSG00000101003	GINS1	9837	0.64	2.14E-06	7.19E-05
Treatment & Medium: Day 1	ENSG00000170515	PA2G4	5036	0.64	2.14E-06	7.19E-05
Treatment & Medium: Day 1	ENSG00000062282	DGAT2	84649	0.89	2.17E-06	7.28E-05
Treatment & Medium: Day 1	ENSG00000255717	SNHG1	23642	0.72	2.19E-06	7.32E-05
Treatment & Medium: Day 1	ENSG00000132530	XAF1	54739	0.07	2.20E-06	7.32E-05
Treatment & Medium: Day 1	ENSG00000130508	PXDN	7837	0.12	2.21E-06	7.36E-05
Treatment & Medium: Day 1	ENSG00000092010	PSME1	5720	0.38	2.22E-06	7.36E-05
Treatment & Medium: Day 1	ENSG00000164053	ATRIP	84126	0.63	2.23E-06	7.37E-05
Treatment & Medium: Day 1	ENSG00000161921	CXCL16	58191	-0.71	2.24E-06	7.39E-05
Treatment & Medium: Day 1	ENSG00000184220	CMSS1	84319	0.61	2.24E-06	7.39E-05
Treatment & Medium: Day 1	ENSG00000187210	GCNT1	2650	0.58	2.27E-06	7.45E-05
Treatment & Medium: Day 1	ENSG00000172594	SMPDL3A	10924	-1.44	2.27E-06	7.46E-05
Treatment & Medium: Day 1	ENSG00000074410	CA12	771	0.29	2.28E-06	7.46E-05
Treatment & Medium: Day 1	ENSG00000214688	C10orf105	414152	-2.05	2.30E-06	7.50E-05
Treatment & Medium: Day 1	ENSG00000119698	PPP4R4	57718	0.87	2.30E-06	7.50E-05
Treatment & Medium: Day 1	ENSG00000120693	SMAD9	4093	-1.36	2.31E-06	7.51E-05
Treatment & Medium: Day 1	ENSG00000171552	BCL2L1	598	0.63	2.32E-06	7.53E-05
Treatment & Medium: Day 1	ENSG00000138735	PDE5A	8654	-1.10	2.34E-06	7.58E-05
Treatment & Medium: Day 1	ENSG00000183454	GRIN2A	2903	0.71	2.35E-06	7.58E-05
Treatment & Medium: Day 1	ENSG00000164045	CDC25A	993	0.84	2.36E-06	7.58E-05
Treatment & Medium: Day 1	ENSG00000167747	C19orf48	84798	0.73	2.36E-06	7.58E-05
Treatment & Medium: Day 1	ENSG00000167202	TBC1D2B	23102	-0.68	2.36E-06	7.58E-05
Treatment & Medium: Day 1	ENSG00000054598	FOXC1	2296	-0.81	2.36E-06	7.58E-05

Treatment & Medium: Day 1	ENSG00000163931	TKT	7086	-0.33	2.37E-06	7.58E-05
Treatment & Medium: Day 1	ENSG00000167600	CYP2S1	29785	-0.32	2.38E-06	7.62E-05
Treatment & Medium: Day 1	ENSG00000204642	HLA-F	3134	0.65	2.39E-06	7.63E-05
Treatment & Medium: Day 1	ENSG00000112759	SLC29A1	2030	0.76	2.41E-06	7.66E-05
Treatment & Medium: Day 1	ENSG00000166562	SEC11C	90701	0.37	2.43E-06	7.73E-05
Treatment & Medium: Day 1	ENSG00000101361	NOP56	10528	0.73	2.49E-06	7.88E-05
Treatment & Medium: Day 1	ENSG00000247626	MARS2	92935	0.79	2.50E-06	7.91E-05
Treatment & Medium: Day 1	ENSG00000180660	MAB21L1	4081	-0.75	2.51E-06	7.91E-05
Treatment & Medium: Day 1	ENSG00000179314	WSCD1	23302	1.26	2.51E-06	7.91E-05
Treatment & Medium: Day 1	ENSG00000171345	KRT19	3880	-0.84	2.55E-06	8.01E-05
Treatment & Medium: Day 1	ENSG00000164237	CMBL	134147	-0.62	2.58E-06	8.08E-05
Treatment & Medium: Day 1	ENSG00000197496	SLC2A10	81031	-0.68	2.62E-06	8.18E-05
Treatment & Medium: Day 1	ENSG00000164694	FNDC1	84624	0.21	2.62E-06	8.18E-05
Treatment & Medium: Day 1	ENSG00000205221	VIT	5212	-1.31	2.63E-06	8.19E-05
Treatment & Medium: Day 1	ENSG00000148848	ADAM12	8038	-0.65	2.63E-06	8.19E-05
Treatment & Medium: Day 1	ENSG00000260916	CCPG1	9236	-0.16	2.66E-06	8.24E-05
Treatment & Medium: Day 1	ENSG00000151503	NCAPD3	23310	0.58	2.66E-06	8.24E-05
Treatment & Medium: Day 1	ENSG00000156113	KCNMA1	3778	-0.05	2.70E-06	8.36E-05
Treatment & Medium: Day 1	ENSG00000051180	RAD51	5888	0.69	2.71E-06	8.36E-05
Treatment & Medium: Day 1	ENSG00000048540	LMO3	55885	-1.07	2.73E-06	8.40E-05
Treatment & Medium: Day 1	ENSG00000124788	ATXN1	6310	-0.88	2.74E-06	8.40E-05
Treatment & Medium: Day 1	ENSG00000125845	BMP2	650	-1.02	2.74E-06	8.40E-05
Treatment & Medium: Day 1	ENSG00000126549	STATH	6779	-2.97	2.74E-06	8.40E-05

Treatment & Medium: Day 1	ENSG00000115687	PASK	23178	0.64	2.75E-06	8.40E-05
Treatment & Medium: Day 1	ENSG00000105518	TMEM205	374882	0.47	2.76E-06	8.42E-05
Treatment & Medium: Day 1	ENSG00000110104	CCDC86	79080	0.62	2.77E-06	8.43E-05
Treatment & Medium: Day 1	ENSG00000178764	ZHX2	22882	-0.86	2.77E-06	8.43E-05
Treatment & Medium: Day 1	ENSG00000067177	PHKA1	5255	0.87	2.79E-06	8.45E-05
Treatment & Medium: Day 1	ENSG00000075651	PLD1	5337	-1.22	2.84E-06	8.60E-05
Treatment & Medium: Day 1	ENSG00000117877	CD3EAP	10849	0.94	2.85E-06	8.61E-05
Treatment & Medium: Day 1	ENSG00000140416	TPM1	7168	-0.27	2.90E-06	8.75E-05
Treatment & Medium: Day 1	ENSG00000197635	DPP4	1803	-0.27	2.92E-06	8.77E-05
Treatment & Medium: Day 1	ENSG00000077943	ITGA8	8516	-1.43	2.94E-06	8.82E-05
Treatment & Medium: Day 1	ENSG00000113368	LMNB1	4001	0.54	2.95E-06	8.82E-05
Treatment & Medium: Day 1	ENSG00000147251	DOCK11	139818	-0.75	2.96E-06	8.82E-05
Treatment & Medium: Day 1	ENSG00000170577	SIX2	10736	-0.80	2.96E-06	8.82E-05
Treatment & Medium: Day 1	ENSG00000134013	LOXL2	4017	-0.36	2.97E-06	8.82E-05
Treatment & Medium: Day 1	ENSG00000105664	COMP	1311	-1.35	2.97E-06	8.82E-05
Treatment & Medium: Day 1	ENSG00000137094	DNAJB5	25822	-0.20	2.98E-06	8.83E-05
Treatment & Medium: Day 1	ENSG00000240184	PCDHGC3	5098	-0.84	2.99E-06	8.84E-05
Treatment & Medium: Day 1	ENSG00000187800	PEAR1	375033	-0.84	2.99E-06	8.84E-05
Treatment & Medium: Day 1	ENSG00000101236	RNF24	11237	-0.67	3.01E-06	8.87E-05
Treatment & Medium: Day 1	ENSG00000028277	POU2F2	5452	0.52	3.07E-06	9.04E-05
Treatment & Medium: Day 1	ENSG00000175274	TP53I11	9537	-0.83	3.10E-06	9.10E-05
Treatment & Medium: Day 1	ENSG00000168309	FAM107A	11170	1.73	3.10E-06	9.10E-05
Treatment & Medium: Day 1	ENSG00000144381	HSPD1	3329	0.65	3.11E-06	9.10E-05

Treatment & Medium: Day 1	ENSG00000125384	PTGER2	5732	0.13	3.11E-06	9.10E-05
Treatment & Medium: Day 1	ENSG00000121039	RDH10	157506	-1.55	3.13E-06	9.12E-05
Treatment & Medium: Day 1	ENSG00000143850	PLEKHA6	22874	-0.75	3.14E-06	9.15E-05
Treatment & Medium: Day 1	ENSG00000095752	IL11	3589	-0.55	3.21E-06	9.34E-05
Treatment & Medium: Day 1	ENSG00000181938	GINS3	64785	0.71	3.22E-06	9.35E-05
Treatment & Medium: Day 1	ENSG00000155903	RASA2	5922	0.01	3.24E-06	9.36E-05
Treatment & Medium: Day 1	ENSG00000169683	LRRC45	201255	0.41	3.24E-06	9.36E-05
Treatment & Medium: Day 1	ENSG00000213366	GSTM2	2946	-0.89	3.26E-06	9.41E-05
Treatment & Medium: Day 1	ENSG00000122042	UBL3	5412	-0.36	3.28E-06	9.46E-05
Treatment & Medium: Day 1	ENSG00000130713	EXOSC2	23404	0.58	3.32E-06	9.52E-05
Treatment & Medium: Day 1	ENSG00000165617	DACT1	51339	-1.67	3.32E-06	9.52E-05
Treatment & Medium: Day 1	ENSG00000129493	HEATR5A	25938	-0.66	3.33E-06	9.54E-05
Treatment & Medium: Day 1	ENSG00000143416	SELENBP1	8991	-0.80	3.40E-06	9.72E-05
Treatment & Medium: Day 1	ENSG00000185532	PRKG1	5592	-1.06	3.42E-06	9.75E-05
Treatment & Medium: Day 1	ENSG00000146250	PRSS35	167681	-1.81	3.43E-06	9.75E-05
Treatment & Medium: Day 1	ENSG00000144354	CDCA7	83879	0.64	3.43E-06	9.75E-05
Treatment & Medium: Day 1	ENSG00000094804	CDC6	990	0.72	3.46E-06	9.82E-05
Treatment & Medium: Day 1	ENSG00000126709	IFI6	2537	0.19	3.47E-06	9.82E-05
Treatment & Medium: Day 1	ENSG00000129467	ADCY4	196883	-0.55	3.47E-06	9.82E-05
Treatment & Medium: Day 1	ENSG00000163737	PF4	5196	3.06	3.49E-06	9.84E-05
Treatment & Medium: Day 1	ENSG00000121281	ADCY7	113	-0.51	3.52E-06	9.90E-05
Treatment & Medium: Day 1	ENSG00000173559	NABP1	64859	-0.48	3.53E-06	9.92E-05
Treatment & Medium: Day 1	ENSG00000162599	NFIA	4774	0.21	3.54E-06	9.92E-05

Treatment & Medium: Day 1	ENSG00000124191	TOX2	84969	0.12	3.54E-06	9.92E-05
Treatment & Medium: Day 1	ENSG00000104951	IL4I1	259307	0.27	3.56E-06	9.94E-05
Treatment & Medium: Day 1	ENSG00000120949	TNFRSF8	943	1.33	3.57E-06	9.94E-05
Treatment & Medium: Day 1	ENSG00000185567	AHNAK2	113146	-0.42	3.57E-06	9.94E-05
Treatment & Medium: Day 1	ENSG00000060982	BCAT1	586	-0.08	3.60E-06	0.000100004
Treatment & Medium: Day 1	ENSG00000139211	AMIGO2	347902	-1.54	3.67E-06	0.000101923
Treatment & Medium: Day 1	ENSG00000182568	SATB1	6304	-1.25	3.70E-06	0.000102434
Treatment & Medium: Day 1	ENSG00000164463	CREBRF	153222	-0.69	3.72E-06	0.000103014
Treatment & Medium: Day 1	ENSG00000171604	CXXC5	51523	-0.53	3.74E-06	0.000103146
Treatment & Medium: Day 1	ENSG00000076685	NT5C2	22978	-0.49	3.77E-06	0.00010392
Treatment & Medium: Day 1	ENSG00000180198	RCC1	1104	0.37	3.78E-06	0.00010392
Treatment & Medium: Day 1	ENSG00000111602	TIMELESS	8914	0.46	3.83E-06	0.000105072
Treatment & Medium: Day 1	ENSG00000136861	CDK5RAP2	55755	-0.23	3.87E-06	0.00010603
Treatment & Medium: Day 1	ENSG00000186193	SAPCD2	89958	0.72	3.88E-06	0.000106252
Treatment & Medium: Day 1	ENSG00000175832	ETV4	2118	0.88	3.89E-06	0.000106252
Treatment & Medium: Day 1	ENSG00000156265	MAP3K7CL	56911	-0.18	3.91E-06	0.00010675
Treatment & Medium: Day 1	ENSG00000179958	DCTPP1	79077	0.65	3.93E-06	0.000106923
Treatment & Medium: Day 1	ENSG00000154640	BTG3	10950	0.59	3.94E-06	0.000107176
Treatment & Medium: Day 1	ENSG00000126458	RRAS	6237	0.18	3.95E-06	0.000107176
Treatment & Medium: Day 1	ENSG00000167244	IGF2	3481	-0.90	3.97E-06	0.000107467
Treatment & Medium: Day 1	ENSG00000122507	BBS9	27241	-0.92	3.98E-06	0.000107558
Treatment & Medium: Day 1	ENSG00000111206	FOXM1	2305	0.42	3.99E-06	0.000107558
Treatment & Medium: Day 1	ENSG00000112312	GMNN	51053	0.64	4.00E-06	0.000107728

Treatment & Medium: Day 1	ENSG00000132780	NASP	4678	0.52	4.05E-06	0.000108846
Treatment & Medium: Day 1	ENSG00000179630	LACC1	144811	-0.14	4.13E-06	0.000110924
Treatment & Medium: Day 1	ENSG00000259207	ITGB3	3690	0.03	4.17E-06	0.000111729
Treatment & Medium: Day 1	ENSG00000164099	PRSS12	8492	-0.71	4.18E-06	0.000111884
Treatment & Medium: Day 1	ENSG00000129667	RHBDF2	79651	0.81	4.20E-06	0.000112264
Treatment & Medium: Day 1	ENSG00000006625	GGCT	79017	0.42	4.23E-06	0.000112923
Treatment & Medium: Day 1	ENSG00000170989	S1PR1	1901	-2.12	4.25E-06	0.000113084
Treatment & Medium: Day 1	ENSG00000139329	LUM	4060	-0.82	4.27E-06	0.000113324
Treatment & Medium: Day 1	ENSG00000213551	DNAJC9	23234	0.56	4.27E-06	0.000113324
Treatment & Medium: Day 1	ENSG00000010404	IDS	3423	-0.47	4.33E-06	0.000114771
Treatment & Medium: Day 1	ENSG00000181072	CHRM2	1129	-0.78	4.37E-06	0.000115444
Treatment & Medium: Day 1	ENSG00000197989	SNHG12	85028	0.77	4.37E-06	0.000115444
Treatment & Medium: Day 1	ENSG00000174600	CMKLR1	1240	-0.93	4.38E-06	0.000115489
Treatment & Medium: Day 1	ENSG00000187244	BCAM	4059	-0.82	4.46E-06	0.000117222
Treatment & Medium: Day 1	ENSG00000084090	STARD7	56910	-0.04	4.47E-06	0.000117371
Treatment & Medium: Day 1	ENSG00000197299	BLM	641	0.79	4.48E-06	0.000117371
Treatment & Medium: Day 1	ENSG00000146555	SDK1	221935	-0.34	4.61E-06	0.000120648
Treatment & Medium: Day 1	ENSG00000171155	C1GALT1C1	29071	0.61	4.65E-06	0.000121395
Treatment & Medium: Day 1	ENSG00000091651	ORC6	23594	0.55	4.66E-06	0.000121575
Treatment & Medium: Day 1	ENSG00000163346	PBXIP1	57326	-0.86	4.68E-06	0.000121911
Treatment & Medium: Day 1	ENSG00000154856	APCDD1	147495	-1.15	4.70E-06	0.000121916
Treatment & Medium: Day 1	ENSG00000011105	TSPAN9	10867	-0.68	4.70E-06	0.000121916
Treatment & Medium: Day 1	ENSG00000160325	CACFD1	11094	-1.42	4.71E-06	0.000121916

Treatment & Medium: Day 1	ENSG00000166949	SMAD3	4088	-0.47	4.80E-06	0.000124062
Treatment & Medium: Day 1	ENSG00000168140	VASN	114990	-0.72	4.83E-06	0.000124661
Treatment & Medium: Day 1	ENSG00000133059	DSTYK	25778	-0.53	4.90E-06	0.000126397
Treatment & Medium: Day 1	ENSG00000140743	CDR2	1039	-0.23	4.92E-06	0.000126717
Treatment & Medium: Day 1	ENSG00000105246	EBI3	10148	3.32	4.97E-06	0.000127475
Treatment & Medium: Day 1	ENSG00000164647	STEAP1	26872	0.43	4.98E-06	0.000127475
Treatment & Medium: Day 1	ENSG00000104856	RELB	5971	0.60	4.98E-06	0.000127475
Treatment & Medium: Day 1	ENSG00000038002	AGA	175	-0.72	4.99E-06	0.000127561
Treatment & Medium: Day 1	ENSG00000137266	SLC22A23	63027	-0.78	5.10E-06	0.000129933
Treatment & Medium: Day 1	ENSG00000167088	SNRPD1	6632	0.57	5.10E-06	0.000129933
Treatment & Medium: Day 1	ENSG00000148841	ITPRIP	85450	0.86	5.12E-06	0.000130175
Treatment & Medium: Day 1	ENSG00000161547	SRSF2	6427	0.45	5.14E-06	0.000130189
Treatment & Medium: Day 1	ENSG00000184205	TSPYL2	64061	-0.23	5.14E-06	0.000130189
Treatment & Medium: Day 1	ENSG00000092445	TYRO3	7301	0.20	5.15E-06	0.000130189
Treatment & Medium: Day 1	ENSG00000164938	TP53INP1	94241	-1.00	5.15E-06	0.000130189
Treatment & Medium: Day 1	ENSG00000086062	B4GALT1	2683	0.07	5.16E-06	0.000130189
Treatment & Medium: Day 1	ENSG00000162745	OLFML2B	25903	-0.72	5.23E-06	0.000131826
Treatment & Medium: Day 1	ENSG00000119986	AVPI1	60370	-0.08	5.24E-06	0.000131826
Treatment & Medium: Day 1	ENSG00000138669	PRKG2	5593	-0.51	5.32E-06	0.000133487
Treatment & Medium: Day 1	ENSG00000134531	EMP1	2012	-0.07	5.33E-06	0.000133487
Treatment & Medium: Day 1	ENSG00000164649	CDCA7L	55536	0.53	5.35E-06	0.000133931
Treatment & Medium: Day 1	ENSG00000100304	TTLL12	23170	0.57	5.42E-06	0.000135414
Treatment & Medium: Day 1	ENSG00000150051	MKX	283078	-0.85	5.49E-06	0.000136876

Treatment & Medium: Day 1	ENSG00000147536	GINS4	84296	0.39	5.51E-06	0.000137195
Treatment & Medium: Day 1	ENSG00000130522	JUND	3727	-0.43	5.58E-06	0.00013867
Treatment & Medium: Day 1	ENSG00000140525	FANCI	55215	0.53	5.62E-06	0.000139431
Treatment & Medium: Day 1	ENSG00000116711	PLA2G4A	5321	-0.43	5.63E-06	0.000139612
Treatment & Medium: Day 1	ENSG00000069011	PITX1	5307	-0.66	5.67E-06	0.000140322
Treatment & Medium: Day 1	ENSG00000048052	HDAC9	9734	0.03	5.72E-06	0.000141358
Treatment & Medium: Day 1	ENSG00000106617	PRKAG2	51422	0.37	5.79E-06	0.000142786
Treatment & Medium: Day 1	ENSG00000243279	PRAF2	11230	-0.06	5.84E-06	0.000143895
Treatment & Medium: Day 1	ENSG00000154319	FAM167A	83648	1.20	5.94E-06	0.000145866
Treatment & Medium: Day 1	ENSG00000176490	DIRAS1	148252	-0.98	5.94E-06	0.000145866
Treatment & Medium: Day 1	ENSG00000171872	KLF17	128209	1.45	5.96E-06	0.00014607
Treatment & Medium: Day 1	ENSG00000172156	CCL11	6356	-0.31	6.00E-06	0.000146763
Treatment & Medium: Day 1	ENSG00000189067	LITAF	9516	-0.66	6.01E-06	0.000146763
Treatment & Medium: Day 1	ENSG00000185507	IRF7	3665	0.35	6.02E-06	0.000146763
Treatment & Medium: Day 1	ENSG00000148229	POLE3	54107	0.50	6.05E-06	0.000147383
Treatment & Medium: Day 1	ENSG00000054793	ATP9A	10079	-0.77	6.06E-06	0.000147466
Treatment & Medium: Day 1	ENSG00000173085	COQ2	27235	0.24	6.14E-06	0.000149012
Treatment & Medium: Day 1	ENSG00000105202	FBL	2091	0.45	6.16E-06	0.000149431
Treatment & Medium: Day 1	ENSG00000115009	CCL20	6364	2.80	6.17E-06	0.000149471
Treatment & Medium: Day 1	ENSG00000185697	MYBL1	4603	0.37	6.19E-06	0.000149685
Treatment & Medium: Day 1	ENSG00000129347	KRI1	65095	0.39	6.20E-06	0.000149685
Treatment & Medium: Day 1	ENSG00000169764	UGP2	7360	0.52	6.22E-06	0.000149842
Treatment & Medium: Day 1	ENSG00000116016	EPAS1	2034	-0.58	6.24E-06	0.000149847

Treatment & Medium: Day 1	ENSG00000049541	RFC2	5982	0.56	6.24E-06	0.000149847
Treatment & Medium: Day 1	ENSG00000111641	NOP2	4839	0.67	6.25E-06	0.000149847
Treatment & Medium: Day 1	ENSG00000178031	ADAMTSL1	92949	-0.76	6.29E-06	0.000150727
Treatment & Medium: Day 1	ENSG00000165244	ZNF367	195828	0.65	6.32E-06	0.000150978
Treatment & Medium: Day 1	ENSG00000164040	PGRMC2	10424	-0.37	6.33E-06	0.000150978
Treatment & Medium: Day 1	ENSG00000177156	TALDO1	6888	-0.33	6.33E-06	0.000150978
Treatment & Medium: Day 1	ENSG00000116260	QSOX1	5768	-0.37	6.34E-06	0.000150978
Treatment & Medium: Day 1	ENSG00000187098	MITF	4286	-0.77	6.36E-06	0.000151056
Treatment & Medium: Day 1	ENSG00000175792	RUVBL1	8607	0.52	6.37E-06	0.000151144
Treatment & Medium: Day 1	ENSG00000158292	GPR153	387509	-0.87	6.43E-06	0.00015227
Treatment & Medium: Day 1	ENSG00000101955	SRPX	8406	-0.31	6.44E-06	0.00015227
Treatment & Medium: Day 1	ENSG00000160072	ATAD3B	83858	0.63	6.48E-06	0.000152732
Treatment & Medium: Day 1	ENSG00000172349	IL16	3603	-0.66	6.48E-06	0.000152732
Treatment & Medium: Day 1	ENSG00000138395	CDK15	65061	0.57	6.54E-06	0.000153979
Treatment & Medium: Day 1	ENSG00000160211	G6PD	2539	-0.71	6.56E-06	0.000154128
Treatment & Medium: Day 1	ENSG00000182240	BACE2	25825	0.39	6.63E-06	0.000155653
Treatment & Medium: Day 1	ENSG00000053372	MRTO4	51154	0.64	6.77E-06	0.000158512
Treatment & Medium: Day 1	ENSG00000134107	BHLHE40	8553	-1.17	6.77E-06	0.000158512
Treatment & Medium: Day 1	ENSG00000165949	IFI27	3429	0.10	6.85E-06	0.000160173
Treatment & Medium: Day 1	ENSG00000134202	GSTM3	2947	-0.59	7.02E-06	0.000163745
Treatment & Medium: Day 1	ENSG00000128973	CLN6	54982	0.57	7.04E-06	0.0001641
Treatment & Medium: Day 1	ENSG00000132382	MYBBP1A	10514	0.60	7.08E-06	0.000164636
Treatment & Medium: Day 1	ENSG00000148154	UGCG	7357	0.12	7.15E-06	0.000165856

Treatment & Medium: Day 1	ENSG00000013573	DDX11	1663	0.68	7.16E-06	0.000165856
Treatment & Medium: Day 1	ENSG00000070404	FSTL3	10272	0.73	7.17E-06	0.000165856
Treatment & Medium: Day 1	ENSG00000141338	ABCA8	10351	-0.80	7.18E-06	0.000165856
Treatment & Medium: Day 1	ENSG00000205542	TMSB4X	7114	-0.71	7.18E-06	0.000165856
Treatment & Medium: Day 1	ENSG00000006016	CRLF1	9244	-0.96	7.25E-06	0.000167084
Treatment & Medium: Day 1	ENSG00000109743	BST1	683	-0.40	7.29E-06	0.000167819
Treatment & Medium: Day 1	ENSG00000072657	TRHDE	29953	-0.70	7.34E-06	0.000168816
Treatment & Medium: Day 1	ENSG00000243244	STON1	11037	-0.75	7.36E-06	0.000168949
Treatment & Medium: Day 1	ENSG00000162520	SYNC	81493	-0.44	7.53E-06	0.000172504
Treatment & Medium: Day 1	ENSG00000124104	SNX21	90203	-0.63	7.58E-06	0.000173356
Treatment & Medium: Day 1	ENSG00000239306	RBM14	10432	0.45	7.60E-06	0.000173356
Treatment & Medium: Day 1	ENSG00000144810	COL8A1	1295	0.50	7.60E-06	0.000173356
Treatment & Medium: Day 1	ENSG00000100292	HMOX1	3162	-0.95	7.73E-06	0.00017607
Treatment & Medium: Day 1	ENSG00000162512	SDC3	9672	-0.64	7.78E-06	0.000176837
Treatment & Medium: Day 1	ENSG00000137203	TFAP2A	7020	-0.88	7.79E-06	0.000176837
Treatment & Medium: Day 1	ENSG00000197451	HNRNPAB	3182	0.52	7.81E-06	0.000176837
Treatment & Medium: Day 1	ENSG00000072422	RHOBTB1	9886	-0.81	7.81E-06	0.000176837
Treatment & Medium: Day 1	ENSG00000148180	GSN	2934	-0.87	7.82E-06	0.000176864
Treatment & Medium: Day 1	ENSG00000104687	GSR	2936	-0.33	7.83E-06	0.000176938
Treatment & Medium: Day 1	ENSG00000213694	S1PR3	1903	-0.68	7.88E-06	0.000177731
Treatment & Medium: Day 1	ENSG00000049759	NEDD4L	23327	-0.17	7.96E-06	0.000179336
Treatment & Medium: Day 1	ENSG00000140526	ABHD2	11057	0.52	8.01E-06	0.000180133
Treatment & Medium: Day 1	ENSG00000187741	FANCA	2175	0.53	8.06E-06	0.000180437

Treatment & Medium: Day 1	ENSG00000115946	PNO1	56902	0.63	8.06E-06	0.000180437
Treatment & Medium: Day 1	ENSG00000140092	FBLN5	10516	-0.82	8.06E-06	0.000180437
Treatment & Medium: Day 1	ENSG00000149380	P4HA3	283208	1.09	8.11E-06	0.0001811
Treatment & Medium: Day 1	ENSG00000137033	IL33	90865	1.22	8.11E-06	0.0001811
Treatment & Medium: Day 1	ENSG00000107175	CREB3	10488	0.12	8.14E-06	0.0001811
Treatment & Medium: Day 1	ENSG00000123096	SSPN	8082	-0.88	8.15E-06	0.0001811
Treatment & Medium: Day 1	ENSG00000118257	NRP2	8828	1.28	8.16E-06	0.0001811
Treatment & Medium: Day 1	ENSG00000147041	SYTL5	94122	-2.36	8.16E-06	0.0001811
Treatment & Medium: Day 1	ENSG00000138642	HERC6	55008	0.52	8.21E-06	0.000181987
Treatment & Medium: Day 1	ENSG00000181458	TMEM45A	55076	-0.09	8.25E-06	0.000182625
Treatment & Medium: Day 1	ENSG00000122952	ZWINT	11130	0.51	8.38E-06	0.00018521
Treatment & Medium: Day 1	ENSG00000155189	AGPAT5	55326	0.80	8.39E-06	0.00018521
Treatment & Medium: Day 1	ENSG00000100321	SYNGR1	9145	-0.71	8.44E-06	0.000185838
Treatment & Medium: Day 1	ENSG00000126016	AMOT	154796	-0.73	8.45E-06	0.000185838
Treatment & Medium: Day 1	ENSG00000149451	ADAM33	80332	-0.58	8.51E-06	0.000187021
Treatment & Medium: Day 1	ENSG00000144554	FANCD2	2177	0.62	8.63E-06	0.000189268
Treatment & Medium: Day 1	ENSG00000115457	IGFBP2	3485	-0.07	8.83E-06	0.00019349
Treatment & Medium: Day 1	ENSG00000074582	BCS1L	617	0.52	8.85E-06	0.000193611
Treatment & Medium: Day 1	ENSG00000158023	WDR66	144406	0.84	8.91E-06	0.00019475
Treatment & Medium: Day 1	ENSG00000138363	ATIC	471	0.40	9.24E-06	0.0002016
Treatment & Medium: Day 1	ENSG00000115380	EFEMP1	2202	-0.65	9.27E-06	0.000201886
Treatment & Medium: Day 1	ENSG00000059915	PSD	5662	-1.15	9.29E-06	0.000202061
Treatment & Medium: Day 1	ENSG00000198554	WDHD1	11169	0.61	9.36E-06	0.000203362

Treatment & Medium: Day 1	ENSG00000150281	CTF1	1489	-0.31	9.38E-06	0.000203436
Treatment & Medium: Day 1	ENSG00000187608	ISG15	9636	0.33	9.42E-06	0.000203872
Treatment & Medium: Day 1	ENSG00000130787	HIP1R	9026	0.22	9.43E-06	0.000203872
Treatment & Medium: Day 1	ENSG00000254122	PCDHGB7	56099	-0.98	9.44E-06	0.000203872
Treatment & Medium: Day 1	ENSG00000165732	DDX21	9188	0.69	9.49E-06	0.000204782
Treatment & Medium: Day 1	ENSG00000114491	UMPS	7372	0.48	9.61E-06	0.00020716
Treatment & Medium: Day 1	ENSG00000102287	GABRE	2564	-0.66	9.65E-06	0.000207179
Treatment & Medium: Day 1	ENSG00000186603	HPDL	84842	1.54	9.65E-06	0.000207179
Treatment & Medium: Day 1	ENSG00000100625	SIX4	51804	-0.82	9.66E-06	0.000207179
Treatment & Medium: Day 1	ENSG00000189057	FAM111B	374393	0.81	9.70E-06	0.000207896
Treatment & Medium: Day 1	ENSG00000177000	MTHFR	4524	-0.79	9.76E-06	0.000208721
Treatment & Medium: Day 1	ENSG00000038427	VCAN	1462	-0.90	9.77E-06	0.000208721
Treatment & Medium: Day 1	ENSG00000142046	TMEM91	641649	-1.37	9.84E-06	0.000209952
Treatment & Medium: Day 1	ENSG00000142227	EMP3	2014	0.24	9.87E-06	0.000210267
Treatment & Medium: Day 1	ENSG00000096063	SRPK1	6732	0.53	9.93E-06	0.000210941
Treatment & Medium: Day 1	ENSG00000108561	C1QBP	708	0.48	9.94E-06	0.000210941
Treatment & Medium: Day 1	ENSG00000173457	PPP1R14B	26472	0.43	9.94E-06	0.000210941
Treatment & Medium: Day 1	ENSG00000168389	MFSD2A	84879	1.06	1.00E-05	0.000212022
Treatment & Medium: Day 1	ENSG00000092964	DPYSL2	1808	-0.45	1.00E-05	0.000212246
Treatment & Medium: Day 1	ENSG00000091129	NRCAM	4897	1.31	1.01E-05	0.000213497
Treatment & Medium: Day 1	ENSG00000100906	NFKBIA	4792	0.96	1.02E-05	0.000214563
Treatment & Medium: Day 1	ENSG00000197321	SVIL	6840	-0.58	1.03E-05	0.000215608
Treatment & Medium: Day 1	ENSG00000170485	NPAS2	4862	0.71	1.03E-05	0.000215608

Treatment & Medium: Day 1	ENSG00000120437	ACAT2	39	0.12	1.03E-05	0.000215608
Treatment & Medium: Day 1	ENSG00000113369	ARRDC3	57561	-0.67	1.03E-05	0.000215608
Treatment & Medium: Day 1	ENSG00000104722	NEFM	4741	-1.11	1.03E-05	0.000215723
Treatment & Medium: Day 1	ENSG00000049246	PER3	8863	-0.96	1.03E-05	0.000215874
Treatment & Medium: Day 1	ENSG00000185585	OLFML2A	169611	-0.22	1.03E-05	0.000215997
Treatment & Medium: Day 1	ENSG00000197380	DACT3	147906	-1.22	1.04E-05	0.000216899
Treatment & Medium: Day 1	ENSG00000161714	PLCD3	113026	-0.61	1.05E-05	0.000219032
Treatment & Medium: Day 1	ENSG00000124249	KCNK15	60598	-1.66	1.06E-05	0.000221134
Treatment & Medium: Day 1	ENSG00000143816	WNT9A	7483	-0.80	1.08E-05	0.000223764
Treatment & Medium: Day 1	ENSG00000115267	IFIH1	64135	0.31	1.08E-05	0.000223822
Treatment & Medium: Day 1	ENSG00000143013	LMO4	8543	-0.22	1.08E-05	0.000225018
Treatment & Medium: Day 1	ENSG00000175197	DDIT3	1649	-0.64	1.09E-05	0.00022534
Treatment & Medium: Day 1	ENSG00000162496	DHRS3	9249	-1.69	1.09E-05	0.000225357
Treatment & Medium: Day 1	ENSG00000100526	CDKN3	1033	0.54	1.09E-05	0.000225806
Treatment & Medium: Day 1	ENSG00000035141	FAM136A	84908	0.44	1.09E-05	0.000225806
Treatment & Medium: Day 1	ENSG00000102967	DHODH	1723	0.58	1.10E-05	0.00022583
Treatment & Medium: Day 1	ENSG00000130204	TOMM40	10452	0.58	1.10E-05	0.00022583
Treatment & Medium: Day 1	ENSG00000167553	TUBA1C	84790	0.54	1.11E-05	0.000228385
Treatment & Medium: Day 1	ENSG00000175305	CCNE2	9134	0.91	1.12E-05	0.000230101
Treatment & Medium: Day 1	ENSG00000100439	ABHD4	63874	-0.81	1.12E-05	0.000230383
Treatment & Medium: Day 1	ENSG00000115548	KDM3A	55818	-0.38	1.14E-05	0.000232299
Treatment & Medium: Day 1	ENSG00000114767	RRP9	9136	0.65	1.14E-05	0.000232619
Treatment & Medium: Day 1	ENSG00000115844	DLX2	1746	-0.97	1.15E-05	0.000233853

Treatment & Medium: Day 1	ENSG00000115226	FNDC4	64838	-0.41	1.15E-05	0.000235066
Treatment & Medium: Day 1	ENSG00000136026	CKAP4	10970	-0.11	1.17E-05	0.000237386
Treatment & Medium: Day 1	ENSG00000152894	PTPRK	5796	-0.71	1.17E-05	0.000237722
Treatment & Medium: Day 1	ENSG00000148158	SNX30	401548	-0.66	1.17E-05	0.000237722
Treatment & Medium: Day 1	ENSG00000106605	BLVRA	644	-0.52	1.17E-05	0.000237722
Treatment & Medium: Day 1	ENSG00000154265	ABCA5	23461	-0.57	1.18E-05	0.000237739
Treatment & Medium: Day 1	ENSG00000006459	KDM7A	80853	-0.99	1.18E-05	0.000237739
Treatment & Medium: Day 1	ENSG00000179750	APOBEC3B	9582	0.60	1.18E-05	0.000238433
Treatment & Medium: Day 1	ENSG00000106823	ECM2	1842	-1.00	1.19E-05	0.000240115
Treatment & Medium: Day 1	ENSG00000064989	CALCRL	10203	0.18	1.19E-05	0.000240115
Treatment & Medium: Day 1	ENSG00000107201	DDX58	23586	0.48	1.19E-05	0.000240357
Treatment & Medium: Day 1	ENSG00000111962	UST	10090	0.05	1.20E-05	0.000240357
Treatment & Medium: Day 1	ENSG00000183668	PSG9	5678	-1.28	1.20E-05	0.000240357
Treatment & Medium: Day 1	ENSG00000156273	BACH1	571	-0.45	1.20E-05	0.000240357
Treatment & Medium: Day 1	ENSG00000159167	STC1	6781	0.84	1.20E-05	0.000240357
Treatment & Medium: Day 1	ENSG00000152409	JMY	133746	-0.47	1.20E-05	0.000240399
Treatment & Medium: Day 1	ENSG00000106799	TGFBR1	7046	-0.63	1.21E-05	0.000240494
Treatment & Medium: Day 1	ENSG00000105486	LIG1	3978	0.36	1.21E-05	0.000240494
Treatment & Medium: Day 1	ENSG00000160193	WDR4	10785	0.65	1.21E-05	0.00024075
Treatment & Medium: Day 1	ENSG00000196730	DAPK1	1612	-0.58	1.22E-05	0.000242596
Treatment & Medium: Day 1	ENSG00000170791	CHCHD7	79145	0.52	1.22E-05	0.000243266
Treatment & Medium: Day 1	ENSG00000156804	FBXO32	114907	-0.91	1.23E-05	0.000243642
Treatment & Medium: Day 1	ENSG00000186185	KIF18B	146909	0.45	1.23E-05	0.000243642

Treatment & Medium: Day 1	ENSG00000167972	ABCA3	21	0.10	1.24E-05	0.000244686
Treatment & Medium: Day 1	ENSG00000056558	TRAF1	7185	1.25	1.24E-05	0.00024542
Treatment & Medium: Day 1	ENSG00000082641	NFE2L1	4779	0.10	1.25E-05	0.000245845
Treatment & Medium: Day 1	ENSG00000100626	GALNT16	57452	-0.75	1.25E-05	0.000246422
Treatment & Medium: Day 1	ENSG00000154146	NRGN	4900	-0.09	1.25E-05	0.000246589
Treatment & Medium: Day 1	ENSG00000055044	NOP58	51602	0.61	1.26E-05	0.000247153
Treatment & Medium: Day 1	ENSG00000104889	RNASEH2A	10535	0.45	1.26E-05	0.000247153
Treatment & Medium: Day 1	ENSG00000181982	CCDC149	91050	-0.72	1.26E-05	0.000247153
Treatment & Medium: Day 1	ENSG00000241878	PISD	23761	0.53	1.26E-05	0.000247153
Treatment & Medium: Day 1	ENSG00000146072	TNFRSF21	27242	-0.61	1.26E-05	0.000247153
Treatment & Medium: Day 1	ENSG00000053254	FOXN3	1112	-0.74	1.28E-05	0.000250649
Treatment & Medium: Day 1	ENSG00000160818	GPATCH4	54865	0.69	1.29E-05	0.000251397
Treatment & Medium: Day 1	ENSG00000138764	CCNG2	901	-0.73	1.29E-05	0.000251462
Treatment & Medium: Day 1	ENSG00000026508	CD44	960	-0.59	1.30E-05	0.000253083
Treatment & Medium: Day 1	ENSG00000123416	TUBA1B	10376	0.42	1.30E-05	0.000253083
Treatment & Medium: Day 1	ENSG00000163814	CDCP1	64866	0.21	1.30E-05	0.000253083
Treatment & Medium: Day 1	ENSG00000005471	ABCB4	5244	-2.36	1.32E-05	0.000254838
Treatment & Medium: Day 1	ENSG00000132840	BHMT2	23743	-0.45	1.32E-05	0.000254838
Treatment & Medium: Day 1	ENSG00000069424	KCNAB2	8514	-0.91	1.32E-05	0.000255802
Treatment & Medium: Day 1	ENSG00000143158	MPC2	25874	0.00	1.34E-05	0.00025891
Treatment & Medium: Day 1	ENSG00000136717	BIN1	274	-0.69	1.35E-05	0.000259904
Treatment & Medium: Day 3	ENSG00000096060	FKBP5	2289	2.00	1.67E-12	1.22E-08

Treatment & Medium: Day 3	ENSG00000135069	PSAT1	29968	0.97	1.18E-12	1.22E-08
Treatment & Medium: Day 3	ENSG00000070669	ASNS	440	0.38	2.39E-12	1.22E-08
Treatment & Medium: Day 3	ENSG00000128165	ADM2	79924	0.35	3.37E-12	1.29E-08
Treatment & Medium: Day 3	ENSG00000134363	FST	10468	-0.88	1.07E-11	3.25E-08
Treatment & Medium: Day 3	ENSG00000185432	METTL7A	25840	1.07	5.87E-11	1.49E-07
Treatment & Medium: Day 3	ENSG00000189058	APOD	347	-0.08	1.23E-10	2.67E-07
Treatment & Medium: Day 3	ENSG00000019582	CD74	972	1.31	1.82E-10	3.48E-07
Treatment & Medium: Day 3	ENSG00000105825	TFPI2	7980	1.48	2.14E-10	3.63E-07
Treatment & Medium: Day 3	ENSG00000163131	CTSS	1520	0.85	2.86E-10	4.25E-07
Treatment & Medium: Day 3	ENSG00000172403	SYNPO2	171024	-0.25	3.06E-10	4.25E-07
Treatment & Medium: Day 3	ENSG00000092621	PHGDH	26227	-0.08	3.92E-10	4.38E-07
Treatment & Medium: Day 3	ENSG00000118503	TNFAIP3	7128	1.35	3.93E-10	4.38E-07
Treatment & Medium: Day 3	ENSG00000023445	BIRC3	330	1.08	4.03E-10	4.38E-07
Treatment & Medium: Day 3	ENSG00000116991	SIPA1L2	57568	-1.34	7.16E-10	7.28E-07
Treatment & Medium: Day 3	ENSG00000198759	EGFL6	25975	-2.31	8.25E-10	7.86E-07
Treatment & Medium: Day 3	ENSG00000221852	KRTAP1-5	83895	-0.34	9.04E-10	8.02E-07
Treatment & Medium: Day 3	ENSG00000204941	PSG5	5673	-1.49	9.47E-10	8.02E-07
Treatment & Medium: Day 3	ENSG00000171345	KRT19	3880	-0.75	1.07E-09	8.17E-07
Treatment & Medium: Day 3	ENSG00000196517	SLC6A9	6536	-0.61	1.17E-09	8.17E-07
Treatment & Medium: Day 3	ENSG00000188487	INSC	387755	2.25	1.17E-09	8.17E-07
Treatment & Medium: Day 3	ENSG00000225783	MIAT	440823	-2.22	1.18E-09	8.17E-07
Treatment & Medium: Day 3	ENSG00000100889	PCK2	5106	0.26	2.10E-09	1.39E-06
Treatment & Medium: Day 3	ENSG00000073756	PTGS2	5743	-1.25	2.39E-09	1.50E-06

Treatment & Medium: Day 3	ENSG00000113361	CDH6	1004	-1.54	2.46E-09	1.50E-06
Treatment & Medium: Day 3	ENSG00000065911	MTHFD2	10797	0.46	2.80E-09	1.64E-06
Treatment & Medium: Day 3	ENSG00000132718	SYT11	23208	-0.16	4.11E-09	2.32E-06
Treatment & Medium: Day 3	ENSG00000137809	ITGA11	22801	-2.06	4.51E-09	2.38E-06
Treatment & Medium: Day 3	ENSG00000133110	POSTN	10631	-3.31	4.54E-09	2.38E-06
Treatment & Medium: Day 3	ENSG00000171509	RXFP1	59350	-0.90	4.97E-09	2.53E-06
Treatment & Medium: Day 3	ENSG00000167244	IGF2	3481	-1.90	5.59E-09	2.74E-06
Treatment & Medium: Day 3	ENSG00000103257	SLC7A5	8140	0.39	5.75E-09	2.74E-06
Treatment & Medium: Day 3	ENSG00000006016	CRLF1	9244	-2.16	6.05E-09	2.79E-06
Treatment & Medium: Day 3	ENSG00000128342	LIF	3976	-0.53	8.04E-09	3.45E-06
Treatment & Medium: Day 3	ENSG00000166482	MFAP4	4239	-1.94	8.20E-09	3.45E-06
Treatment & Medium: Day 3	ENSG00000049246	PER3	8863	-2.39	8.33E-09	3.45E-06
Treatment & Medium: Day 3	ENSG00000100739	BDKRB1	623	0.00	8.43E-09	3.45E-06
Treatment & Medium: Day 3	ENSG00000176597	B3GNT5	84002	1.44	8.60E-09	3.45E-06
Treatment & Medium: Day 3	ENSG00000173432	SAA1	6288	3.80	1.05E-08	4.02E-06
Treatment & Medium: Day 3	ENSG00000137033	IL33	90865	0.53	1.07E-08	4.02E-06
Treatment & Medium: Day 3	ENSG00000124766	SOX4	6659	-1.53	1.08E-08	4.02E-06
Treatment & Medium: Day 3	ENSG00000108797	CNTNAP1	8506	0.17	1.11E-08	4.02E-06
Treatment & Medium: Day 3	ENSG00000196878	LAMB3	3914	0.23	1.25E-08	4.40E-06
Treatment & Medium: Day 3	ENSG00000154864	PIEZO2	63895	-1.48	1.27E-08	4.40E-06
Treatment & Medium: Day 3	ENSG00000128965	CHAC1	79094	0.62	1.42E-08	4.81E-06
Treatment & Medium: Day 3	ENSG00000139211	AMIGO2	347902	-1.32	1.62E-08	5.36E-06
Treatment & Medium: Day 3	ENSG00000184985	SORCS2	57537	-0.20	1.77E-08	5.74E-06

Treatment & Medium: Day 3	ENSG00000048540	LMO3	55885	-0.50	2.08E-08	6.60E-06
Treatment & Medium: Day 3	ENSG00000143127	ITGA10	8515	2.70	2.41E-08	7.51E-06
Treatment & Medium: Day 3	ENSG00000120337	TNFSF18	8995	0.22	2.64E-08	8.04E-06
Treatment & Medium: Day 3	ENSG00000157150	TIMP4	7079	1.23	2.99E-08	8.93E-06
Treatment & Medium: Day 3	ENSG00000185565	LSAMP	4045	-1.81	3.07E-08	8.99E-06
Treatment & Medium: Day 3	ENSG00000101265	RASSF2	9770	-2.41	3.19E-08	9.19E-06
Treatment & Medium: Day 3	ENSG00000135821	GLUL	2752	0.63	3.53E-08	9.98E-06
Treatment & Medium: Day 3	ENSG00000174348	PODN	127435	-2.29	3.73E-08	1.03E-05
Treatment & Medium: Day 3	ENSG00000162745	OLFML2B	25903	-1.58	3.79E-08	1.03E-05
Treatment & Medium: Day 3	ENSG00000109906	ZBTB16	7704	5.03	3.89E-08	1.04E-05
Treatment & Medium: Day 3	ENSG00000050344	NFE2L3	9603	1.16	4.14E-08	1.09E-05
Treatment & Medium: Day 3	ENSG00000041982	TNC	3371	0.14	4.58E-08	1.18E-05
Treatment & Medium: Day 3	ENSG00000253368	TRNP1	388610	1.30	4.72E-08	1.20E-05
Treatment & Medium: Day 3	ENSG00000163347	CLDN1	9076	1.53	4.81E-08	1.20E-05
Treatment & Medium: Day 3	ENSG00000117594	HSD11B1	3290	2.95	4.89E-08	1.20E-05
Treatment & Medium: Day 3	ENSG00000104611	SH2D4A	63898	-0.78	5.36E-08	1.28E-05
Treatment & Medium: Day 3	ENSG00000174938	SEZ6L2	26470	-0.29	5.38E-08	1.28E-05
Treatment & Medium: Day 3	ENSG00000234745	HLA-B	3106	0.42	6.45E-08	1.49E-05
Treatment & Medium: Day 3	ENSG00000136010	ALDH1L2	160428	0.15	6.45E-08	1.49E-05
Treatment & Medium: Day 3	ENSG00000189221	MAOA	4128	1.82	6.65E-08	1.49E-05
Treatment & Medium: Day 3	ENSG00000172061	LRRC15	131578	-2.03	6.67E-08	1.49E-05
Treatment & Medium: Day 3	ENSG00000163735	CXCL5	6374	4.25	7.13E-08	1.57E-05
Treatment & Medium: Day 3	ENSG00000189056	RELN	5649	-0.84	7.26E-08	1.58E-05

Treatment & Medium: Day 3	ENSG00000211445	GPX3	2878	-1.21	7.59E-08	1.62E-05
Treatment & Medium: Day 3	ENSG00000150630	VEGFC	7424	-0.05	7.63E-08	1.62E-05
Treatment & Medium: Day 3	ENSG00000187955	COL14A1	7373	-2.17	7.95E-08	1.63E-05
Treatment & Medium: Day 3	ENSG00000141448	GATA6	2627	-0.68	8.16E-08	1.63E-05
Treatment & Medium: Day 3	ENSG00000169855	ROBO1	6091	-0.33	8.24E-08	1.63E-05
Treatment & Medium: Day 3	ENSG00000197632	SERPINB2	5055	1.78	8.32E-08	1.63E-05
Treatment & Medium: Day 3	ENSG00000085117	CD82	3732	0.87	8.36E-08	1.63E-05
Treatment & Medium: Day 3	ENSG00000149131	SERPING1	710	-0.85	8.40E-08	1.63E-05
Treatment & Medium: Day 3	ENSG00000119714	GPR68	8111	-0.32	8.46E-08	1.63E-05
Treatment & Medium: Day 3	ENSG00000115902	SLC1A4	6509	-0.40	8.68E-08	1.63E-05
Treatment & Medium: Day 3	ENSG00000154654	NCAM2	4685	-1.74	8.74E-08	1.63E-05
Treatment & Medium: Day 3	ENSG00000075223	SEMA3C	10512	-0.17	8.76E-08	1.63E-05
Treatment & Medium: Day 3	ENSG00000146555	SDK1	221935	-0.72	8.89E-08	1.63E-05
Treatment & Medium: Day 3	ENSG00000162692	VCAM1	7412	-1.17	9.85E-08	1.79E-05
Treatment & Medium: Day 3	ENSG00000169715	MT1E	4493	1.58	1.01E-07	1.81E-05
Treatment & Medium: Day 3	ENSG00000127954	STEAP4	79689	1.31	1.02E-07	1.81E-05
Treatment & Medium: Day 3	ENSG00000105664	COMP	1311	-2.64	1.05E-07	1.84E-05
Treatment & Medium: Day 3	ENSG00000123243	ITIH5	80760	-1.00	1.07E-07	1.84E-05
Treatment & Medium: Day 3	ENSG00000109610	SOD3	6649	-1.54	1.07E-07	1.84E-05
Treatment & Medium: Day 3	ENSG00000147852	VLDLR	7436	-0.92	1.10E-07	1.87E-05
Treatment & Medium: Day 3	ENSG00000120708	TGFBI	7045	-1.50	1.12E-07	1.88E-05
Treatment & Medium: Day 3	ENSG00000122884	P4HA1	5033	0.01	1.18E-07	1.95E-05
Treatment & Medium: Day 3	ENSG00000136859	ANGPTL2	23452	-1.38	1.27E-07	2.04E-05

Treatment & Medium: Day 3	ENSG00000124749	COL21A1	81578	-1.29	1.27E-07	2.04E-05
Treatment & Medium: Day 3	ENSG00000046653	GPM6B	2824	2.37	1.27E-07	2.04E-05
Treatment & Medium: Day 3	ENSG00000104081	BMF	90427	-1.85	1.32E-07	2.10E-05
Treatment & Medium: Day 3	ENSG00000145777	TSLP	85480	-0.12	1.37E-07	2.13E-05
Treatment & Medium: Day 3	ENSG00000179630	LACC1	144811	-0.03	1.37E-07	2.13E-05
Treatment & Medium: Day 3	ENSG00000174136	RGMB	285704	-0.59	1.48E-07	2.27E-05
Treatment & Medium: Day 3	ENSG00000179094	PER1	5187	1.12	1.49E-07	2.27E-05
Treatment & Medium: Day 3	ENSG00000123700	KCNJ2	3759	-0.45	1.52E-07	2.30E-05
Treatment & Medium: Day 3	ENSG00000162591	MEGF6	1953	-0.72	1.57E-07	2.35E-05
Treatment & Medium: Day 3	ENSG00000124191	TOX2	84969	0.56	1.81E-07	2.65E-05
Treatment & Medium: Day 3	ENSG00000206503	HLA-A	3105	0.25	1.81E-07	2.65E-05
Treatment & Medium: Day 3	ENSG00000134259	NGF	4803	-0.90	1.84E-07	2.67E-05
Treatment & Medium: Day 3	ENSG00000006118	TMEM132A	54972	0.76	1.92E-07	2.76E-05
Treatment & Medium: Day 3	ENSG00000172348	RCAN2	10231	-1.54	2.04E-07	2.91E-05
Treatment & Medium: Day 3	ENSG00000157368	IL34	146433	-0.41	2.20E-07	3.07E-05
Treatment & Medium: Day 3	ENSG00000152463	OLAH	55301	2.89	2.20E-07	3.07E-05
Treatment & Medium: Day 3	ENSG00000092969	TGFB2	7042	-1.59	2.27E-07	3.12E-05
Treatment & Medium: Day 3	ENSG00000179104	TMTC2	160335	-1.06	2.27E-07	3.12E-05
Treatment & Medium: Day 3	ENSG00000154175	ABI3BP	25890	0.68	2.30E-07	3.13E-05
Treatment & Medium: Day 3	ENSG00000065534	MYLK	4638	-0.01	2.36E-07	3.18E-05
Treatment & Medium: Day 3	ENSG00000196526	AFAP1	60312	-0.21	2.38E-07	3.18E-05
Treatment & Medium: Day 3	ENSG00000153879	CEBPG	1054	0.33	2.56E-07	3.40E-05
Treatment & Medium: Day 3	ENSG00000188157	AGRN	375790	-0.03	2.59E-07	3.41E-05

Treatment & Medium: Day 3	ENSG00000170214	ADRA1B	147	0.78	2.85E-07	3.72E-05
Treatment & Medium: Day 3	ENSG00000141150	RASL10B	91608	0.41	3.13E-07	4.02E-05
Treatment & Medium: Day 3	ENSG00000183287	CCBE1	147372	0.98	3.13E-07	4.02E-05
Treatment & Medium: Day 3	ENSG00000141469	SLC14A1	6563	-2.88	3.31E-07	4.20E-05
Treatment & Medium: Day 3	ENSG00000119630	PGF	5228	-0.89	3.44E-07	4.34E-05
Treatment & Medium: Day 3	ENSG00000179388	EGR3	1960	-1.65	3.74E-07	4.67E-05
Treatment & Medium: Day 3	ENSG00000110900	TSPAN11	441631	-1.52	3.82E-07	4.73E-05
Treatment & Medium: Day 3	ENSG00000109072	VTN	7448	-1.65	3.93E-07	4.83E-05
Treatment & Medium: Day 3	ENSG00000174697	LEP	3952	2.26	4.23E-07	5.16E-05
Treatment & Medium: Day 3	ENSG00000029153	ARNTL2	56938	0.40	4.43E-07	5.32E-05
Treatment & Medium: Day 3	ENSG00000164307	ERAP1	51752	0.04	4.45E-07	5.32E-05
Treatment & Medium: Day 3	ENSG00000113645	WWC1	23286	1.25	4.47E-07	5.32E-05
Treatment & Medium: Day 3	ENSG00000134070	IRAK2	3656	0.42	4.54E-07	5.37E-05
Treatment & Medium: Day 3	ENSG00000171246	NPTX1	4884	-1.25	4.57E-07	5.37E-05
Treatment & Medium: Day 3	ENSG00000087494	PTHLH	5744	-1.65	4.69E-07	5.46E-05
Treatment & Medium: Day 3	ENSG00000159674	SPON2	10417	-1.42	4.86E-07	5.61E-05
Treatment & Medium: Day 3	ENSG00000164171	ITGA2	3673	0.12	4.90E-07	5.61E-05
Treatment & Medium: Day 3	ENSG00000110492	MDK	4192	-0.77	5.13E-07	5.80E-05
Treatment & Medium: Day 3	ENSG00000204525	HLA-C	3107	0.02	5.14E-07	5.80E-05
Treatment & Medium: Day 3	ENSG00000125148	MT2A	4502	1.57	5.25E-07	5.88E-05
Treatment & Medium: Day 3	ENSG00000149571	KIRREL3	84623	0.61	5.30E-07	5.88E-05
Treatment & Medium: Day 3	ENSG00000111670	GNPTAB	79158	0.03	5.32E-07	5.88E-05
Treatment & Medium: Day 3	ENSG00000136244	IL6	3569	0.34	5.66E-07	6.21E-05

Treatment & Medium: Day 3	ENSG00000144959	NCEH1	57552	0.53	5.80E-07	6.29E-05
Treatment & Medium: Day 3	ENSG00000101825	MXRA5	25878	-1.05	5.82E-07	6.29E-05
Treatment & Medium: Day 3	ENSG00000158716	DUSP23	54935	0.36	6.13E-07	6.58E-05
Treatment & Medium: Day 3	ENSG00000198624	CCDC69	26112	0.23	6.34E-07	6.76E-05
Treatment & Medium: Day 3	ENSG00000197646	PDCD1LG2	80380	-0.01	6.39E-07	6.76E-05
Treatment & Medium: Day 3	ENSG00000135373	EHF	26298	3.02	6.50E-07	6.84E-05
Treatment & Medium: Day 3	ENSG00000152952	PLOD2	5352	0.27	6.71E-07	6.99E-05
Treatment & Medium: Day 3	ENSG00000129009	ISLR	3671	-1.52	6.74E-07	6.99E-05
Treatment & Medium: Day 3	ENSG00000116711	PLA2G4A	5321	-0.45	7.43E-07	7.65E-05
Treatment & Medium: Day 3	ENSG00000164379	FOXQ1	94234	-1.24	7.60E-07	7.78E-05
Treatment & Medium: Day 3	ENSG00000160200	CBS	875	0.71	7.74E-07	7.86E-05
Treatment & Medium: Day 3	ENSG00000162616	DNAJB4	11080	0.98	7.80E-07	7.87E-05
Treatment & Medium: Day 3	ENSG00000049540	ELN	2006	-1.41	7.87E-07	7.90E-05
Treatment & Medium: Day 3	ENSG00000136048	DRAM1	55332	0.23	8.04E-07	8.01E-05
Treatment & Medium: Day 3	ENSG00000177363	LRRN4CL	221091	-1.02	8.10E-07	8.02E-05
Treatment & Medium: Day 3	ENSG00000135362	PRR5L	79899	1.10	8.42E-07	8.28E-05
Treatment & Medium: Day 3	ENSG00000196562	SULF2	55959	-1.53	8.49E-07	8.30E-05
Treatment & Medium: Day 3	ENSG00000164619	BMPER	168667	-0.11	8.73E-07	8.34E-05
Treatment & Medium: Day 3	ENSG00000132000	PODNL1	79883	-0.94	8.73E-07	8.34E-05
Treatment & Medium: Day 3	ENSG00000179314	WSCD1	23302	1.55	8.75E-07	8.34E-05
Treatment & Medium: Day 3	ENSG00000183049	CAMK1D	57118	-0.21	8.76E-07	8.34E-05
Treatment & Medium: Day 3	ENSG00000181634	TNFSF15	9966	0.60	8.84E-07	8.34E-05
Treatment & Medium: Day 3	ENSG00000163520	FBLN2	2199	-1.13	8.86E-07	8.34E-05

Treatment & Medium: Day 3	ENSG00000064601	CTSA	5476	-0.73	9.05E-07	8.42E-05
Treatment & Medium: Day 3	ENSG00000211448	DIO2	1734	-0.63	9.09E-07	8.42E-05
Treatment & Medium: Day 3	ENSG00000139329	LUM	4060	-1.27	9.14E-07	8.42E-05
Treatment & Medium: Day 3	ENSG00000137869	CYP19A1	1588	0.77	9.22E-07	8.42E-05
Treatment & Medium: Day 3	ENSG00000107562	CXCL12	6387	-1.72	9.22E-07	8.42E-05
Treatment & Medium: Day 3	ENSG00000162878	PKDCC	91461	-0.06	9.28E-07	8.42E-05
Treatment & Medium: Day 3	ENSG00000106123	EPHB6	2051	0.57	9.41E-07	8.48E-05
Treatment & Medium: Day 3	ENSG00000187193	MT1X	4501	1.48	9.49E-07	8.48E-05
Treatment & Medium: Day 3	ENSG00000163491	NEK10	152110	-0.59	9.51E-07	8.48E-05
Treatment & Medium: Day 3	ENSG00000182199	SHMT2	6472	0.40	9.74E-07	8.62E-05
Treatment & Medium: Day 3	ENSG00000152953	STK32B	55351	-1.11	9.78E-07	8.62E-05
Treatment & Medium: Day 3	ENSG00000120885	CLU	1191	-0.96	9.94E-07	8.71E-05
Treatment & Medium: Day 3	ENSG00000204642	HLA-F	3134	0.43	1.00E-06	8.74E-05
Treatment & Medium: Day 3	ENSG00000170577	SIX2	10736	-0.55	1.01E-06	8.77E-05
Treatment & Medium: Day 3	ENSG00000120899	PTK2B	2185	-0.82	1.02E-06	8.81E-05
Treatment & Medium: Day 3	ENSG00000166592	RRAD	6236	0.14	1.05E-06	8.99E-05
Treatment & Medium: Day 3	ENSG00000138031	ADCY3	109	-0.40	1.09E-06	9.25E-05
Treatment & Medium: Day 3	ENSG00000166311	SMPD1	6609	-0.56	1.13E-06	9.58E-05
Treatment & Medium: Day 3	ENSG00000184304	PRKD1	5587	0.77	1.14E-06	9.64E-05
Treatment & Medium: Day 3	ENSG00000175040	CHST2	9435	0.86	1.20E-06	0.000100289
Treatment & Medium: Day 3	ENSG00000169908	TM4SF1	4071	1.42	1.25E-06	0.000104022
Treatment & Medium: Day 3	ENSG00000062282	DGAT2	84649	0.34	1.30E-06	0.000107496
Treatment & Medium: Day 3	ENSG00000106809	OGN	4969	-2.95	1.31E-06	0.000107592

Treatment & Medium: Day 3	ENSG00000047457	CP	1356	-0.46	1.32E-06	0.000108491
Treatment & Medium: Day 3	ENSG00000174080	CTSF	8722	-1.00	1.37E-06	0.00011196
Treatment & Medium: Day 3	ENSG00000120437	ACAT2	39	0.35	1.39E-06	0.000112524
Treatment & Medium: Day 3	ENSG00000135919	SERPINE2	5270	-0.84	1.41E-06	0.000114044
Treatment & Medium: Day 3	ENSG00000154856	APCDD1	147495	-1.57	1.42E-06	0.000114342
Treatment & Medium: Day 3	ENSG00000130513	GDF15	9518	-0.64	1.49E-06	0.0001192
Treatment & Medium: Day 3	ENSG00000176974	SHMT1	6470	0.57	1.50E-06	0.000119424
Treatment & Medium: Day 3	ENSG00000187210	GCNT1	2650	0.88	1.54E-06	0.000121238
Treatment & Medium: Day 3	ENSG00000121039	RDH10	157506	-1.21	1.54E-06	0.000121238
Treatment & Medium: Day 3	ENSG00000166448	TMEM130	222865	-1.79	1.63E-06	0.000127338
Treatment & Medium: Day 3	ENSG00000155511	GRIA1	2890	0.70	1.66E-06	0.000128261
Treatment & Medium: Day 3	ENSG00000171867	PRNP	5621	-1.08	1.66E-06	0.000128261
Treatment & Medium: Day 3	ENSG00000137266	SLC22A23	63027	-0.73	1.67E-06	0.000128261
Treatment & Medium: Day 3	ENSG00000115380	EFEMP1	2202	-0.51	1.75E-06	0.000133757
Treatment & Medium: Day 3	ENSG00000138411	HECW2	57520	-0.27	1.79E-06	0.000136032
Treatment & Medium: Day 3	ENSG00000101255	TRIB3	57761	0.06	1.79E-06	0.000136032
Treatment & Medium: Day 3	ENSG00000113657	DPYSL3	1809	-0.21	1.81E-06	0.000136709
Treatment & Medium: Day 3	ENSG00000172197	MBOAT1	154141	-0.73	1.86E-06	0.000139026
Treatment & Medium: Day 3	ENSG00000084674	APOB	338	1.81	1.87E-06	0.000139026
Treatment & Medium: Day 3	ENSG00000128045	RASL11B	65997	1.51	1.87E-06	0.000139026
Treatment & Medium: Day 3	ENSG00000182197	EXT1	2131	-0.50	1.90E-06	0.000140175
Treatment & Medium: Day 3	ENSG00000104321	TRPA1	8989	1.29	1.90E-06	0.000140175
Treatment & Medium: Day 3	ENSG00000099860	GADD45B	4616	0.32	1.95E-06	0.000143033

Treatment & Medium: Day 3	ENSG00000074410	CA12	771	0.34	1.96E-06	0.000143033
Treatment & Medium: Day 3	ENSG00000164929	BAALC	79870	-2.10	1.98E-06	0.000143505
Treatment & Medium: Day 3	ENSG00000162804	SNED1	25992	-1.10	2.00E-06	0.000144747
Treatment & Medium: Day 3	ENSG00000166741	NNMT	4837	0.63	2.06E-06	0.000148029
Treatment & Medium: Day 3	ENSG00000108691	CCL2	6347	0.77	2.08E-06	0.000148029
Treatment & Medium: Day 3	ENSG00000175183	CSRP2	1466	-0.98	2.08E-06	0.000148029
Treatment & Medium: Day 3	ENSG00000144354	CDCA7	83879	0.44	2.11E-06	0.000149511
Treatment & Medium: Day 3	ENSG00000113369	ARRDC3	57561	-0.75	2.13E-06	0.000150368
Treatment & Medium: Day 3	ENSG00000231924	PSG1	5669	-1.42	2.26E-06	0.000158321
Treatment & Medium: Day 3	ENSG00000162104	ADCY9	115	-0.39	2.26E-06	0.000158321
Treatment & Medium: Day 3	ENSG00000168679	SLC16A4	9122	-0.36	2.28E-06	0.00015883
Treatment & Medium: Day 3	ENSG00000125675	GRIA3	2892	-0.31	2.31E-06	0.00015992
Treatment & Medium: Day 3	ENSG00000198848	CES1	1066	-0.75	2.33E-06	0.000160462
Treatment & Medium: Day 3	ENSG00000168685	IL7R	3575	0.41	2.37E-06	0.000162397
Treatment & Medium: Day 3	ENSG00000116584	ARHGEF2	9181	0.00	2.38E-06	0.000162397
Treatment & Medium: Day 3	ENSG00000156265	MAP3K7CL	56911	0.15	2.41E-06	0.000163966
Treatment & Medium: Day 3	ENSG00000120693	SMAD9	4093	-0.85	2.49E-06	0.00016843
Treatment & Medium: Day 3	ENSG00000060656	PTPRU	10076	-0.41	2.56E-06	0.000172721
Treatment & Medium: Day 3	ENSG00000184575	XPOT	11260	0.19	2.58E-06	0.000172964
Treatment & Medium: Day 3	ENSG00000112139	MDGA1	266727	-1.53	2.59E-06	0.000172964
Treatment & Medium: Day 3	ENSG00000038382	TRIO	7204	0.05	2.60E-06	0.000172964
Treatment & Medium: Day 3	ENSG00000122035	RASL11A	387496	0.07	2.63E-06	0.000172964
Treatment & Medium: Day 3	ENSG00000176697	BDNF	627	-0.95	2.63E-06	0.000172964

Treatment & Medium: Day 3	ENSG00000197381	ADARB1	104	-0.77	2.63E-06	0.000172964
Treatment & Medium: Day 3	ENSG00000087245	MMP2	4313	-0.61	2.66E-06	0.000174098
Treatment & Medium: Day 3	ENSG00000170891	CYTL1	54360	-1.37	2.68E-06	0.000174098
Treatment & Medium: Day 3	ENSG00000151632	AKR1C2	1646	-0.38	2.69E-06	0.000174098
Treatment & Medium: Day 3	ENSG00000138385	SSB	6741	0.80	2.70E-06	0.000174098
Treatment & Medium: Day 3	ENSG00000132170	PPARG	5468	0.42	2.71E-06	0.000174098
Treatment & Medium: Day 3	ENSG00000104856	RELB	5971	0.49	2.76E-06	0.000176613
Treatment & Medium: Day 3	ENSG00000261115	TMEM178B	100507421	0.71	2.77E-06	0.000176768
Treatment & Medium: Day 3	ENSG00000115828	QPCT	25797	0.17	2.82E-06	0.000178903
Treatment & Medium: Day 3	ENSG00000169122	FAM110B	90362	-0.55	2.84E-06	0.000179933
Treatment & Medium: Day 3	ENSG00000077943	ITGA8	8516	-1.87	2.87E-06	0.000180611
Treatment & Medium: Day 3	ENSG00000108960	MMD	23531	0.89	2.88E-06	0.000180611
Treatment & Medium: Day 3	ENSG00000112902	SEMA5A	9037	-0.50	2.97E-06	0.000185593
Treatment & Medium: Day 3	ENSG00000143369	ECM1	1893	-0.65	3.04E-06	0.000188456
Treatment & Medium: Day 3	ENSG00000197415	VEPH1	79674	-0.17	3.05E-06	0.000188456
Treatment & Medium: Day 3	ENSG00000140961	OSGIN1	29948	-0.48	3.05E-06	0.000188456
Treatment & Medium: Day 3	ENSG00000152223	EPG5	57724	-0.13	3.13E-06	0.000192614
Treatment & Medium: Day 3	ENSG00000146233	CYP39A1	51302	-0.64	3.16E-06	0.00019328
Treatment & Medium: Day 3	ENSG00000122176	FMOD	2331	-1.34	3.19E-06	0.000194284
Treatment & Medium: Day 3	ENSG00000175130	MARCKSL1	65108	-1.30	3.23E-06	0.000196141
Treatment & Medium: Day 3	ENSG00000101938	CHRDL1	91851	-1.12	3.25E-06	0.000196141
Treatment & Medium: Day 3	ENSG00000168398	BDKRB2	624	-0.48	3.25E-06	0.000196141
Treatment & Medium: Day 3	ENSG00000188042	ARL4C	10123	-0.84	3.32E-06	0.000199001

Treatment & Medium: Day 3	ENSG00000109472	CPE	1363	-1.11	3.42E-06	0.000203857
Treatment & Medium: Day 3	ENSG00000118515	SGK1	6446	-0.76	3.42E-06	0.000203857
Treatment & Medium: Day 3	ENSG00000105989	WNT2	7472	-1.77	3.51E-06	0.000208075
Treatment & Medium: Day 3	ENSG00000138379	MSTN	2660	1.79	3.53E-06	0.000208616
Treatment & Medium: Day 3	ENSG00000123610	TNFAIP6	7130	-0.24	3.65E-06	0.00021506
Treatment & Medium: Day 3	ENSG00000104738	MCM4	4173	0.43	3.68E-06	0.000215763
Treatment & Medium: Day 3	ENSG00000162599	NFIA	4774	0.02	3.69E-06	0.000215763
Treatment & Medium: Day 3	ENSG00000182985	CADM1	23705	-0.70	3.71E-06	0.000216173
Treatment & Medium: Day 3	ENSG00000128815	WDFY4	57705	1.35	3.74E-06	0.000216925
Treatment & Medium: Day 3	ENSG00000151892	GFRA1	2674	-0.63	3.81E-06	0.00021982
Treatment & Medium: Day 3	ENSG00000065060	UHRF1BP1	54887	-0.12	3.84E-06	0.000220918
Treatment & Medium: Day 3	ENSG00000072422	RHOBTB1	9886	-0.97	3.97E-06	0.000227504
Treatment & Medium: Day 3	ENSG00000184254	ALDH1A3	220	-0.49	4.02E-06	0.00022977
Treatment & Medium: Day 3	ENSG00000173852	DPY19L1	23333	-0.12	4.06E-06	0.000230785
Treatment & Medium: Day 3	ENSG00000164920	OSR2	116039	-0.75	4.07E-06	0.000230785
Treatment & Medium: Day 3	ENSG00000125378	BMP4	652	-0.74	4.09E-06	0.000230785
Treatment & Medium: Day 3	ENSG00000113368	LMNB1	4001	0.78	4.12E-06	0.000231532
Treatment & Medium: Day 3	ENSG00000105894	PTN	5764	-1.27	4.13E-06	0.000231532
Treatment & Medium: Day 3	ENSG00000011105	TSPAN9	10867	-0.38	4.15E-06	0.000231532
Treatment & Medium: Day 3	ENSG00000140416	TPM1	7168	-0.14	4.18E-06	0.00023248
Treatment & Medium: Day 3	ENSG00000140092	FBLN5	10516	-0.80	4.20E-06	0.000232809
Treatment & Medium: Day 3	ENSG00000231925	TAPBP	6892	0.22	4.23E-06	0.000233499
Treatment & Medium: Day 3	ENSG00000187957	DNER	92737	1.33	4.36E-06	0.000239796

Treatment & Medium: Day 3	ENSG00000142798	HSPG2	3339	-0.18	4.37E-06	0.000239796
Treatment & Medium: Day 3	ENSG00000183578	TNFAIP8L3	388121	0.35	4.43E-06	0.000242129
Treatment & Medium: Day 3	ENSG00000106538	RARRES2	5919	-2.07	4.46E-06	0.000242654
Treatment & Medium: Day 3	ENSG00000049759	NEDD4L	23327	-0.45	4.47E-06	0.000242654
Treatment & Medium: Day 3	ENSG00000168140	VASN	114990	-0.55	4.52E-06	0.000244475
Treatment & Medium: Day 3	ENSG00000131386	GALNT15	117248	1.16	4.63E-06	0.00024955
Treatment & Medium: Day 3	ENSG00000164104	HMGB2	3148	0.60	4.74E-06	0.000253619
Treatment & Medium: Day 3	ENSG00000113739	STC2	8614	0.41	4.74E-06	0.000253619
Treatment & Medium: Day 3	ENSG00000177706	FAM20C	56975	-0.16	4.94E-06	0.000262505
Treatment & Medium: Day 3	ENSG00000134339	SAA2	6289	3.18	4.94E-06	0.000262505
Treatment & Medium: Day 3	ENSG00000134198	TSPAN2	10100	-1.50	5.01E-06	0.000262505
Treatment & Medium: Day 3	ENSG00000156113	KCNMA1	3778	-0.13	5.01E-06	0.000262505
Treatment & Medium: Day 3	ENSG00000091490	SEL1L3	23231	-0.55	5.02E-06	0.000262505
Treatment & Medium: Day 3	ENSG00000141574	SECTM1	6398	-0.99	5.03E-06	0.000262505
Treatment & Medium: Day 3	ENSG00000205364	MT1M	4499	1.41	5.04E-06	0.000262505
Treatment & Medium: Day 3	ENSG00000081923	ATP8B1	5205	0.12	5.04E-06	0.000262505
Treatment & Medium: Day 3	ENSG00000152580	IGSF10	285313	-1.45	5.13E-06	0.000265804
Treatment & Medium: Day 3	ENSG00000197635	DPP4	1803	-0.30	5.18E-06	0.000267701
Treatment & Medium: Day 3	ENSG00000170989	S1PR1	1901	-0.67	5.20E-06	0.000267874
Treatment & Medium: Day 3	ENSG00000006747	SCIN	85477	-1.84	5.22E-06	0.000268225
Treatment & Medium: Day 3	ENSG00000127948	POR	5447	0.06	5.30E-06	0.000270292
Treatment & Medium: Day 3	ENSG00000164484	TMEM200A	114801	-0.98	5.30E-06	0.000270292
Treatment & Medium: Day 3	ENSG00000182389	CACNB4	785	-1.31	5.38E-06	0.000273293

Treatment & Medium: Day 3	ENSG00000129493	HEATR5A	25938	-0.50	5.46E-06	0.000276419
Treatment & Medium: Day 3	ENSG00000178573	MAF	4094	-1.34	5.48E-06	0.000276696
Treatment & Medium: Day 3	ENSG00000242265	PEG10	23089	0.46	5.73E-06	0.000288399
Treatment & Medium: Day 3	ENSG00000116260	QSOX1	5768	-0.13	5.80E-06	0.000290981
Treatment & Medium: Day 3	ENSG00000168209	DDIT4	54541	0.25	6.01E-06	0.000300408
Treatment & Medium: Day 3	ENSG00000124201	ZNFX1	57169	-0.24	6.05E-06	0.000301317
Treatment & Medium: Day 3	ENSG00000226950	DANCR	57291	0.65	6.10E-06	0.000302829
Treatment & Medium: Day 3	ENSG00000146250	PRSS35	167681	-1.29	6.12E-06	0.000302829
Treatment & Medium: Day 3	ENSG00000141576	RNF157	114804	0.44	6.36E-06	0.000312794
Treatment & Medium: Day 3	ENSG00000105499	PLA2G4C	8605	-0.24	6.36E-06	0.000312794
Treatment & Medium: Day 3	ENSG00000119138	KLF9	687	0.01	6.38E-06	0.000312794
Treatment & Medium: Day 3	ENSG00000115461	IGFBP5	3488	-1.60	6.81E-06	0.000332769
Treatment & Medium: Day 3	ENSG00000134107	BHLHE40	8553	-1.58	6.95E-06	0.00033807
Treatment & Medium: Day 3	ENSG00000104332	SFRP1	6422	-1.11	6.96E-06	0.00033807
Treatment & Medium: Day 3	ENSG00000170962	PDGFD	80310	-1.38	7.06E-06	0.000341564
Treatment & Medium: Day 3	ENSG00000182809	CRIP2	1397	0.17	7.11E-06	0.000342871
Treatment & Medium: Day 3	ENSG00000198910	L1CAM	3897	-1.17	7.13E-06	0.000342871
Treatment & Medium: Day 3	ENSG00000164776	PHKG1	5260	-1.34	7.17E-06	0.000343858
Treatment & Medium: Day 3	ENSG00000144218	AFF3	3899	-1.29	7.21E-06	0.000344666
Treatment & Medium: Day 3	ENSG00000184347	SLIT3	6586	-1.23	7.26E-06	0.000346046
Treatment & Medium: Day 3	ENSG00000103381	CPPED1	55313	0.63	7.36E-06	0.000349121
Treatment & Medium: Day 3	ENSG00000173918	C1QTNF1	114897	0.80	7.37E-06	0.000349121
Treatment & Medium: Day 3	ENSG00000065923	SLC9A7	84679	-0.12	7.44E-06	0.000351168

Treatment & Medium: Day 3	ENSG00000092068	SLC7A8	23428	-0.51	7.48E-06	0.000352173
Treatment & Medium: Day 3	ENSG00000008517	IL32	9235	2.17	7.55E-06	0.000353199
Treatment & Medium: Day 3	ENSG00000175899	A2M	2	-1.48	7.55E-06	0.000353199
Treatment & Medium: Day 3	ENSG00000180875	GREM2	64388	-0.98	7.77E-06	0.000362373
Treatment & Medium: Day 3	ENSG00000135074	ADAM19	8728	-0.63	7.95E-06	0.000369199
Treatment & Medium: Day 3	ENSG00000102595	UGGT2	55757	-0.21	7.99E-06	0.000369199
Treatment & Medium: Day 3	ENSG00000147100	SLC16A2	6567	-0.46	7.99E-06	0.000369199
Treatment & Medium: Day 3	ENSG00000107984	DKK1	22943	0.86	8.09E-06	0.000372509
Treatment & Medium: Day 3	ENSG00000116191	RALGPS2	55103	-0.36	8.15E-06	0.000374127
Treatment & Medium: Day 3	ENSG00000099998	GGT5	2687	1.68	8.23E-06	0.000376912
Treatment & Medium: Day 3	ENSG00000082641	NFE2L1	4779	0.01	8.27E-06	0.000377744
Treatment & Medium: Day 3	ENSG00000101333	PLCB4	5332	0.62	8.34E-06	0.000379623
Treatment & Medium: Day 3	ENSG00000055163	CYFIP2	26999	0.25	8.52E-06	0.00038605
Treatment & Medium: Day 3	ENSG00000123737	EXOSC9	5393	0.44	8.53E-06	0.00038605
Treatment & Medium: Day 3	ENSG00000163431	LMOD1	25802	-0.44	8.66E-06	0.000390758
Treatment & Medium: Day 3	ENSG00000164109	MAD2L1	4085	0.72	8.70E-06	0.00039121
Treatment & Medium: Day 3	ENSG00000182580	EPHB3	2049	-1.47	8.73E-06	0.000391407
Treatment & Medium: Day 3	ENSG00000149968	MMP3	4314	-0.63	8.79E-06	0.000392871
Treatment & Medium: Day 3	ENSG00000180155	LYNX1	66004	-0.63	8.83E-06	0.000393678
Treatment & Medium: Day 3	ENSG00000110031	LPXN	9404	0.52	8.95E-06	0.000397064
Treatment & Medium: Day 3	ENSG00000088882	CPXM1	56265	-1.08	8.98E-06	0.000397064
Treatment & Medium: Day 3	ENSG00000143815	LBR	3930	0.17	9.01E-06	0.000397064
Treatment & Medium: Day 3	ENSG00000166033	HTRA1	5654	-0.67	9.01E-06	0.000397064

Treatment & Medium: Day 3	ENSG00000260549	MT1L	4500	1.43	9.06E-06	0.000397618
Treatment & Medium: Day 3	ENSG00000071539	TRIP13	9319	0.88	9.08E-06	0.000397618
Treatment & Medium: Day 3	ENSG00000072274	TFRC	7037	0.60	9.11E-06	0.000397618
Treatment & Medium: Day 3	ENSG00000117399	CDC20	991	0.82	9.14E-06	0.000397618
Treatment & Medium: Day 3	ENSG00000161921	CXCL16	58191	-0.96	9.15E-06	0.000397618
Treatment & Medium: Day 3	ENSG00000172156	CCL11	6356	0.77	9.22E-06	0.000399157
Treatment & Medium: Day 3	ENSG00000103710	RASL12	51285	-0.67	9.24E-06	0.000399292
Treatment & Medium: Day 3	ENSG00000163584	RPL22L1	200916	0.96	9.31E-06	0.000401102
Treatment & Medium: Day 3	ENSG00000114098	ARMC8	25852	0.54	9.50E-06	0.000407074
Treatment & Medium: Day 3	ENSG00000152377	SPOCK1	6695	-1.23	9.51E-06	0.000407074
Treatment & Medium: Day 3	ENSG00000145819	ARHGAP26	23092	-0.24	9.56E-06	0.000407074
Treatment & Medium: Day 3	ENSG00000176658	MYO1D	4642	-0.97	9.58E-06	0.000407074
Treatment & Medium: Day 3	ENSG00000141338	ABCA8	10351	-0.57	9.60E-06	0.000407074
Treatment & Medium: Day 3	ENSG00000173376	NDNF	79625	-1.66	9.61E-06	0.000407074
Treatment & Medium: Day 3	ENSG00000112096	SOD2	6648	0.81	9.77E-06	0.000411991
Treatment & Medium: Day 3	ENSG00000126803	HSPA2	3306	-0.51	9.84E-06	0.000411991
Treatment & Medium: Day 3	ENSG00000267365	KCNJ2-AS1	400617	-0.41	9.86E-06	0.000411991
Treatment & Medium: Day 3	ENSG00000144381	HSPD1	3329	0.61	9.88E-06	0.000411991
Treatment & Medium: Day 3	ENSG00000115963	RND3	390	0.08	9.88E-06	0.000411991
Treatment & Medium: Day 3	ENSG00000085662	AKR1B1	231	0.98	9.89E-06	0.000411991
Treatment & Medium: Day 3	ENSG00000144802	NFKBIZ	64332	-0.55	1.00E-05	0.000416208
Treatment & Medium: Day 3	ENSG00000152402	GUCY1A2	2977	-0.59	1.00E-05	0.000416208
Treatment & Medium: Day 3	ENSG00000095303	PTGS1	5742	1.21	1.03E-05	0.00042593

Treatment & Medium: Day 3	ENSG00000154309	DISP1	84976	-0.81	1.04E-05	0.000429375
Treatment & Medium: Day 3	ENSG00000175538	KCNE3	10008	-1.28	1.05E-05	0.000429375
Treatment & Medium: Day 3	ENSG00000121898	CPXM2	119587	-0.44	1.05E-05	0.000429375
Treatment & Medium: Day 3	ENSG00000168779	SHOX2	6474	-0.63	1.06E-05	0.000432723
Treatment & Medium: Day 3	ENSG00000143819	EPHX1	2052	-0.62	1.06E-05	0.000432797
Treatment & Medium: Day 3	ENSG00000138449	SLC40A1	30061	-1.23	1.06E-05	0.000432797
Treatment & Medium: Day 3	ENSG00000169744	LDB2	9079	-1.15	1.09E-05	0.000441739
Treatment & Medium: Day 3	ENSG00000117643	MAN1C1	57134	-1.05	1.09E-05	0.000441739
Treatment & Medium: Day 3	ENSG00000169239	CA5B	11238	-0.27	1.10E-05	0.00044345
Treatment & Medium: Day 3	ENSG00000142731	PLK4	10733	0.67	1.11E-05	0.000447485
Treatment & Medium: Day 3	ENSG00000170390	DCLK2	166614	-0.83	1.14E-05	0.000455558
Treatment & Medium: Day 3	ENSG00000087116	ADAMTS2	9509	-0.05	1.16E-05	0.000465367
Treatment & Medium: Day 3	ENSG00000185633	NDUFA4L2	56901	-1.01	1.17E-05	0.000467707
Treatment & Medium: Day 3	ENSG00000135218	CD36	948	0.00	1.18E-05	0.00046848
Treatment & Medium: Day 3	ENSG00000198612	COPS8	10920	0.45	1.20E-05	0.000474626
Treatment & Medium: Day 3	ENSG00000112936	C7	730	0.42	1.20E-05	0.000474793
Treatment & Medium: Day 3	ENSG00000040275	SPDL1	54908	0.53	1.21E-05	0.000476211
Treatment & Medium: Day 3	ENSG00000116741	RGS2	5997	-1.41	1.23E-05	0.00048163
Treatment & Medium: Day 3	ENSG00000196549	MME	4311	0.03	1.23E-05	0.00048163
Treatment & Medium: Day 3	ENSG00000179954	SSC5D	284297	-0.88	1.23E-05	0.00048163
Treatment & Medium: Day 3	ENSG00000106976	DNM1	1759	-1.08	1.25E-05	0.000488444
Treatment & Medium: Day 3	ENSG00000134057	CCNB1	891	0.82	1.26E-05	0.000488444
Treatment & Medium: Day 3	ENSG00000198932	GPRASP1	9737	-1.03	1.26E-05	0.000488444

Treatment & Medium: Day 3	ENSG00000162614	NEXN	91624	0.73	1.26E-05	0.000488444
Treatment & Medium: Day 3	ENSG00000145244	CORIN	10699	1.58	1.26E-05	0.000488444
Treatment & Medium: Day 3	ENSG00000186417	GLDN	342035	-0.97	1.27E-05	0.000489845
Treatment & Medium: Day 3	ENSG00000048052	HDAC9	9734	-0.17	1.28E-05	0.000493026
Treatment & Medium: Day 3	ENSG00000159403	C1R	715	-0.67	1.29E-05	0.000495155
Treatment & Medium: Day 3	ENSG00000158966	CACHD1	57685	-0.78	1.30E-05	0.000496859
Treatment & Medium: Day 3	ENSG00000013588	GPRC5A	9052	-0.37	1.34E-05	0.000511168
Treatment & Medium: Day 3	ENSG00000106617	PRKAG2	51422	0.70	1.35E-05	0.000513977
Treatment & Medium: Day 3	ENSG00000137501	SYTL2	54843	-1.29	1.36E-05	0.000515976
Treatment & Medium: Day 3	ENSG00000144824	PHLDB2	90102	-0.36	1.36E-05	0.000516635
Treatment & Medium: Day 3	ENSG00000184557	SOCS3	9021	-0.55	1.39E-05	0.000524002
Treatment & Medium: Day 3	ENSG00000112984	KIF20A	10112	0.84	1.42E-05	0.000536018
Treatment & Medium: Day 3	ENSG00000168955	TM4SF20	79853	-1.61	1.43E-05	0.000536018
Treatment & Medium: Day 3	ENSG00000143382	ADAMTSL4	54507	-0.55	1.43E-05	0.000536018
Treatment & Medium: Day 3	ENSG00000145335	SNCA	6622	-0.45	1.43E-05	0.000536018
Treatment & Medium: Day 3	ENSG00000169992	NLGN2	57555	-0.44	1.45E-05	0.000540323
Treatment & Medium: Day 3	ENSG00000134247	PTGFRN	5738	-1.18	1.45E-05	0.00054194
Treatment & Medium: Day 3	ENSG00000145390	USP53	54532	0.47	1.46E-05	0.000542685
Treatment & Medium: Day 3	ENSG00000146232	NFKBIE	4794	0.61	1.47E-05	0.000545368
Treatment & Medium: Day 3	ENSG00000168309	FAM107A	11170	1.84	1.48E-05	0.00054678
Treatment & Medium: Day 3	ENSG00000126458	RRAS	6237	0.36	1.49E-05	0.00054678
Treatment & Medium: Day 3	ENSG00000131370	SH3BP5	9467	-0.63	1.49E-05	0.00054678
Treatment & Medium: Day 3	ENSG00000197405	C5AR1	728	2.48	1.49E-05	0.00054678

Treatment & Medium: Day 3	ENSG00000145147	SLIT2	9353	-0.10	1.49E-05	0.00054678
Treatment & Medium: Day 3	ENSG00000139209	SLC38A4	55089	-1.32	1.50E-05	0.00054678
Treatment & Medium: Day 3	ENSG00000176046	NUPR1	26471	-0.30	1.52E-05	0.000555602
Treatment & Medium: Day 3	ENSG00000087586	AURKA	6790	0.61	1.53E-05	0.000557114
Treatment & Medium: Day 3	ENSG00000123179	EBPL	84650	0.70	1.55E-05	0.000563151
Treatment & Medium: Day 3	ENSG00000076382	SPAG5	10615	0.74	1.55E-05	0.000563151
Treatment & Medium: Day 3	ENSG00000054598	FOXC1	2296	-0.88	1.57E-05	0.000566078
Treatment & Medium: Day 3	ENSG00000106333	PCOLCE	5118	-0.59	1.59E-05	0.000570432
Treatment & Medium: Day 3	ENSG00000103742	IGDCC4	57722	-0.22	1.59E-05	0.000570432
Treatment & Medium: Day 3	ENSG00000145604	SKP2	6502	0.00	1.60E-05	0.000575418
Treatment & Medium: Day 3	ENSG00000101057	MYBL2	4605	0.86	1.63E-05	0.000582296
Treatment & Medium: Day 3	ENSG00000160325	CACFD1	11094	-0.81	1.65E-05	0.000588417
Treatment & Medium: Day 3	ENSG00000111684	LPCAT3	10162	0.11	1.66E-05	0.000590927
Treatment & Medium: Day 3	ENSG00000006062	MAP3K14	9020	-0.85	1.67E-05	0.000594228
Treatment & Medium: Day 3	ENSG00000221869	CEBPD	1052	0.14	1.69E-05	0.00059856
Treatment & Medium: Day 3	ENSG00000088325	TPX2	22974	0.70	1.70E-05	0.00059856
Treatment & Medium: Day 3	ENSG00000125730	C3	718	0.86	1.70E-05	0.00059856
Treatment & Medium: Day 3	ENSG00000182606	TRAK1	22906	-0.21	1.70E-05	0.00059856
Treatment & Medium: Day 3	ENSG00000010932	FMO1	2326	-1.24	1.70E-05	0.000598777
Treatment & Medium: Day 3	ENSG00000124134	KCNS1	3787	-1.59	1.71E-05	0.000599181
Treatment & Medium: Day 3	ENSG00000165617	DACT1	51339	-1.37	1.72E-05	0.000601265
Treatment & Medium: Day 3	ENSG00000178031	ADAMTSL1	92949	-0.90	1.72E-05	0.000601265
Treatment & Medium: Day 3	ENSG00000197496	SLC2A10	81031	-0.28	1.74E-05	0.000605436

Treatment & Medium: Day 3	ENSG00000157168	NRG1	3084	-0.11	1.75E-05	0.00060598
Treatment & Medium: Day 3	ENSG00000120498	TEX11	56159	2.51	1.75E-05	0.00060598
Treatment & Medium: Day 3	ENSG00000148154	UGCG	7357	0.21	1.76E-05	0.000608705
Treatment & Medium: Day 3	ENSG00000174371	EXO1	9156	0.65	1.78E-05	0.00061247
Treatment & Medium: Day 3	ENSG00000072210	ALDH3A2	224	-0.90	1.79E-05	0.000615592
Treatment & Medium: Day 3	ENSG00000148848	ADAM12	8038	-0.70	1.79E-05	0.000615956
Treatment & Medium: Day 3	ENSG00000172889	EGFL7	51162	-0.62	1.84E-05	0.000628875
Treatment & Medium: Day 3	ENSG00000158292	GPR153	387509	-0.67	1.85E-05	0.000631074
Treatment & Medium: Day 3	ENSG00000133216	EPHB2	2048	-1.04	1.86E-05	0.000633425
Treatment & Medium: Day 3	ENSG00000164099	PRSS12	8492	-0.58	1.88E-05	0.000638477
Treatment & Medium: Day 3	ENSG00000139364	TMEM132B	114795	-1.36	1.89E-05	0.000641493
Treatment & Medium: Day 3	ENSG00000163739	CXCL1	2919	1.68	1.93E-05	0.000653656
Treatment & Medium: Day 3	ENSG00000253276	CCDC71L	168455	-0.28	1.95E-05	0.000660056
Treatment & Medium: Day 3	ENSG00000143248	RGS5	8490	-1.75	1.96E-05	0.000660056
Treatment & Medium: Day 3	ENSG00000124875	CXCL6	6372	2.47	1.97E-05	0.000662508
Treatment & Medium: Day 3	ENSG00000122861	PLAU	5328	-0.06	1.99E-05	0.000667602
Treatment & Medium: Day 3	ENSG00000128602	SMO	6608	-1.23	2.00E-05	0.000669921
Treatment & Medium: Day 3	ENSG00000119969	HELLS	3070	0.56	2.01E-05	0.000673445
Treatment & Medium: Day 3	ENSG00000155760	FZD7	8324	-0.69	2.02E-05	0.000673445
Treatment & Medium: Day 3	ENSG00000115009	CCL20	6364	3.18	2.02E-05	0.000673776
Treatment & Medium: Day 3	ENSG00000156486	KCNS2	3788	-2.08	2.03E-05	0.000674807
Treatment & Medium: Day 3	ENSG00000110987	BCL7A	605	0.12	2.06E-05	0.000684294
Treatment & Medium: Day 3	ENSG00000104951	IL4I1	259307	-0.23	2.08E-05	0.000685634

Treatment & Medium: Day 3	ENSG00000167972	ABCA3	21	-0.13	2.08E-05	0.000685634
Treatment & Medium: Day 3	ENSG00000131018	SYNE1	23345	-0.47	2.08E-05	0.000685634
Treatment & Medium: Day 3	ENSG00000172575	RASGRP1	10125	0.11	2.10E-05	0.000687859
Treatment & Medium: Day 3	ENSG00000076003	MCM6	4175	0.11	2.10E-05	0.000687859
Treatment & Medium: Day 3	ENSG00000188483	IER5L	389792	-0.89	2.15E-05	0.000702204
Treatment & Medium: Day 3	ENSG00000137193	PIM1	5292	-0.60	2.16E-05	0.000703465
Treatment & Medium: Day 3	ENSG00000164611	PTTG1	9232	0.96	2.16E-05	0.000703465
Treatment & Medium: Day 3	ENSG00000138135	CH25H	9023	0.06	2.17E-05	0.000703465
Treatment & Medium: Day 3	ENSG00000144476	ACKR3	57007	-1.30	2.17E-05	0.000703465
Treatment & Medium: Day 3	ENSG00000102287	GABRE	2564	-1.05	2.17E-05	0.000703465
Treatment & Medium: Day 3	ENSG00000074935	TUBE1	51175	0.12	2.22E-05	0.000718589
Treatment & Medium: Day 3	ENSG00000158714	SLAMF8	56833	3.75	2.23E-05	0.000718694
Treatment & Medium: Day 3	ENSG00000176692	FOXC2	2303	1.17	2.23E-05	0.000718694
Treatment & Medium: Day 3	ENSG00000168056	LTBP3	4054	-0.66	2.25E-05	0.000722707
Treatment & Medium: Day 3	ENSG00000173597	SULT1B1	27284	0.48	2.27E-05	0.000726913
Treatment & Medium: Day 3	ENSG00000102802	MEDAG	84935	0.42	2.28E-05	0.000727832
Treatment & Medium: Day 3	ENSG00000171848	RRM2	6241	0.60	2.31E-05	0.000735465
Treatment & Medium: Day 3	ENSG00000013293	SLC7A14	57709	-2.50	2.31E-05	0.000736595
Treatment & Medium: Day 3	ENSG00000158023	WDR66	144406	0.88	2.34E-05	0.000741778
Treatment & Medium: Day 3	ENSG00000100504	PYGL	5836	-0.27	2.34E-05	0.000741778
Treatment & Medium: Day 3	ENSG00000140945	CDH13	1012	-0.10	2.35E-05	0.000742727
Treatment & Medium: Day 3	ENSG00000198885	ITPRIPL1	150771	0.89	2.38E-05	0.000749883
Treatment & Medium: Day 3	ENSG00000145386	CCNA2	890	0.79	2.42E-05	0.000762307

Treatment & Medium: Day 3	ENSG00000181982	CCDC149	91050	-0.63	2.44E-05	0.000767509
Treatment & Medium: Day 3	ENSG00000109805	NCAPG	64151	0.69	2.46E-05	0.000772763
Treatment & Medium: Day 3	ENSG00000011028	MRC2	9902	-0.65	2.51E-05	0.000785641
Treatment & Medium: Day 3	ENSG00000154319	FAM167A	83648	0.96	2.53E-05	0.000790668
Treatment & Medium: Day 3	ENSG00000137675	MMP27	64066	-1.38	2.54E-05	0.000790668
Treatment & Medium: Day 3	ENSG00000113580	NR3C1	2908	-0.63	2.56E-05	0.000797912
Treatment & Medium: Day 3	ENSG00000180660	MAB21L1	4081	-0.89	2.59E-05	0.000804091
Treatment & Medium: Day 3	ENSG00000151692	RNF144A	9781	-0.45	2.60E-05	0.000804184
Treatment & Medium: Day 3	ENSG00000171522	PTGER4	5734	0.92	2.60E-05	0.000804184
Treatment & Medium: Day 3	ENSG00000080573	COL5A3	50509	-0.51	2.63E-05	0.000810693
Treatment & Medium: Day 3	ENSG00000138669	PRKG2	5593	-0.87	2.63E-05	0.000810693
Treatment & Medium: Day 3	ENSG00000072571	HMMR	3161	0.77	2.65E-05	0.00081519
Treatment & Medium: Day 3	ENSG00000243137	PSG4	5672	-0.30	2.66E-05	0.000815847
Treatment & Medium: Day 3	ENSG00000108854	SMURF2	64750	-0.03	2.66E-05	0.000815847
Treatment & Medium: Day 3	ENSG00000214688	C10orf105	414152	-1.78	2.68E-05	0.00081787
Treatment & Medium: Day 3	ENSG00000108604	SMARCD2	6603	0.34	2.68E-05	0.000818381
Treatment & Medium: Day 3	ENSG00000164649	CDCA7L	55536	0.43	2.70E-05	0.000820899
Treatment & Medium: Day 3	ENSG00000243753	HLA-L	3139	0.21	2.73E-05	0.000829272
Treatment & Medium: Day 3	ENSG00000146674	IGFBP3	3486	-1.72	2.76E-05	0.000836465
Treatment & Medium: Day 3	ENSG00000154589	LY96	23643	-0.32	2.77E-05	0.000837449
Treatment & Medium: Day 3	ENSG00000148677	ANKRD1	27063	2.60	2.78E-05	0.000838443
Treatment & Medium: Day 3	ENSG00000141404	GNAL	2774	-0.91	2.78E-05	0.000838443
Treatment & Medium: Day 3	ENSG00000139514	SLC7A1	6541	0.08	2.82E-05	0.000845998

Treatment & Medium: Day 3	ENSG00000253910	PCDHGB2	56103	-0.91	2.82E-05	0.000845998
Treatment & Medium: Day 3	ENSG00000159763	PIP	5304	1.41	2.82E-05	0.000845998
Treatment & Medium: Day 3	ENSG00000115594	IL1R1	3554	-0.40	2.84E-05	0.000847812
Treatment & Medium: Day 3	ENSG00000156011	PSD3	23362	-1.00	2.85E-05	0.000849115
Treatment & Medium: Day 3	ENSG00000011465	DCN	1634	-0.32	2.86E-05	0.000850768
Treatment & Medium: Day 3	ENSG00000167123	CERCAM	51148	-0.37	2.86E-05	0.000850768
Treatment & Medium: Day 3	ENSG00000116641	DOCK7	85440	-0.13	2.90E-05	0.000860722
Treatment & Medium: Day 3	ENSG00000131153	GINS2	51659	0.73	2.96E-05	0.000874756
Treatment & Medium: Day 3	ENSG00000166341	DCHS1	8642	-0.79	2.97E-05	0.000874756
Treatment & Medium: Day 3	ENSG00000140285	FGF7	2252	-0.51	2.97E-05	0.000874756
Treatment & Medium: Day 3	ENSG00000063438	AHRR	57491	-0.56	2.99E-05	0.000879801
Treatment & Medium: Day 3	ENSG00000126368	NR1D1	9572	0.31	3.01E-05	0.000884721
Treatment & Medium: Day 3	ENSG00000164220	F2RL2	2151	-0.89	3.02E-05	0.000884721
Treatment & Medium: Day 3	ENSG00000131238	PPT1	5538	0.30	3.02E-05	0.000884721
Treatment & Medium: Day 3	ENSG00000116285	ERRFI1	54206	-0.45	3.05E-05	0.000890951
Treatment & Medium: Day 3	ENSG00000117069	ST6GALNAC5	81849	-1.26	3.07E-05	0.000893688
Treatment & Medium: Day 3	ENSG00000128641	MYO1B	4430	0.14	3.08E-05	0.000896985
Treatment & Medium: Day 3	ENSG00000164694	FNDC1	84624	-1.03	3.09E-05	0.000897041
Treatment & Medium: Day 3	ENSG00000122862	SRGN	5552	0.88	3.11E-05	0.000900856
Treatment & Medium: Day 3	ENSG00000158467	AHCYL2	23382	-1.05	3.11E-05	0.000900856
Treatment & Medium: Day 3	ENSG00000106366	SERPINE1	5054	0.52	3.13E-05	0.000903459
Treatment & Medium: Day 3	ENSG00000101955	SRPX	8406	0.01	3.15E-05	0.000908948
Treatment & Medium: Day 3	ENSG00000077157	PPP1R12B	4660	-0.15	3.16E-05	0.000909702

Treatment & Medium: Day 3	ENSG00000162595	DIRAS3	9077	-1.32	3.20E-05	0.000917867
Treatment & Medium: Day 3	ENSG00000067177	PHKA1	5255	0.60	3.20E-05	0.000917867
Treatment & Medium: Day 3	ENSG00000196923	PDLIM7	9260	-0.15	3.21E-05	0.000918771
Treatment & Medium: Day 3	ENSG00000003436	TFPI	7035	0.21	3.22E-05	0.000918771
Treatment & Medium: Day 3	ENSG00000140044	JDP2	122953	-0.29	3.22E-05	0.000918771
Treatment & Medium: Day 3	ENSG00000138685	FGF2	2247	-0.12	3.24E-05	0.000920774
Treatment & Medium: Day 3	ENSG00000185697	MYBL1	4603	0.29	3.28E-05	0.000931023
Treatment & Medium: Day 3	ENSG00000167600	CYP2S1	29785	-0.25	3.29E-05	0.000932617
Treatment & Medium: Day 3	ENSG00000172986	GXYLT2	727936	-1.02	3.37E-05	0.000952407
Treatment & Medium: Day 3	ENSG00000033867	SLC4A7	9497	-0.29	3.38E-05	0.000954583
Treatment & Medium: Day 3	ENSG00000149633	KIAA1755	85449	-0.99	3.43E-05	0.000963314
Treatment & Medium: Day 3	ENSG00000158089	GALNT14	79623	-0.50	3.43E-05	0.000963314
Treatment & Medium: Day 3	ENSG00000130766	SESN2	83667	-0.17	3.43E-05	0.000963314
Treatment & Medium: Day 3	ENSG00000146648	EGFR	1956	-0.32	3.44E-05	0.00096407
Treatment & Medium: Day 3	ENSG00000139192	TAPBPL	55080	-0.07	3.46E-05	0.000966926
Treatment & Medium: Day 3	ENSG00000077942	FBLN1	2192	-0.41	3.49E-05	0.000974648
Treatment & Medium: Day 3	ENSG00000115844	DLX2	1746	-1.10	3.53E-05	0.000982586
Treatment & Medium: Day 3	ENSG00000183454	GRIN2A	2903	0.80	3.56E-05	0.000990643
Treatment & Medium: Day 3	ENSG00000166949	SMAD3	4088	-0.29	3.57E-05	0.000992158
Treatment & Medium: Day 3	ENSG00000111371	SLC38A1	81539	-0.65	3.58E-05	0.000992158
Treatment & Medium: Day 3	ENSG00000143320	CRABP2	1382	-1.05	3.59E-05	0.000992158
Treatment & Medium: Day 3	ENSG00000035664	DAPK2	23604	-0.01	3.61E-05	0.000997896
Treatment & Medium: Day 3	ENSG00000100297	MCM5	4174	0.46	3.62E-05	0.000999429

Treatment & Medium: Day 3	ENSG00000111424	VDR	7421	-0.31	3.65E-05	0.001002797
Treatment & Medium: Day 3	ENSG00000162496	DHRS3	9249	-1.41	3.65E-05	0.001002797
Treatment & Medium: Day 3	ENSG00000239474	KLHL41	10324	-0.75	3.66E-05	0.001003949
Treatment & Medium: Day 3	ENSG00000143228	NUF2	83540	0.82	3.75E-05	0.001025309
Treatment & Medium: Day 3	ENSG00000160326	SLC2A6	11182	0.76	3.77E-05	0.001029912
Treatment & Medium: Day 3	ENSG00000149591	TAGLN	6876	-0.23	3.78E-05	0.001029912
Treatment & Medium: Day 3	ENSG00000238266	LINC00707	100507127	0.81	3.80E-05	0.001033364
Treatment & Medium: Day 3	ENSG00000135931	ARMC9	80210	-0.87	3.81E-05	0.001034551
Treatment & Medium: Day 3	ENSG00000090339	ICAM1	3383	1.00	3.81E-05	0.001034551
Treatment & Medium: Day 3	ENSG00000138778	CENPE	1062	0.67	3.85E-05	0.001042949
Treatment & Medium: Day 3	ENSG00000136275	C7orf69	80099	-0.37	3.87E-05	0.001045067
Treatment & Medium: Day 3	ENSG00000025434	NR1H3	10062	0.28	3.89E-05	0.001047593
Treatment & Medium: Day 3	ENSG00000170373	CST1	1469	-0.63	3.89E-05	0.001047593
Treatment & Medium: Day 3	ENSG00000116690	PRG4	10216	-1.49	3.90E-05	0.001047593
Treatment & Medium: Day 3	ENSG00000100100	PIK3IP1	113791	-0.54	3.90E-05	0.001047593
Treatment & Medium: Day 3	ENSG00000035499	DEPDC1B	55789	0.75	3.92E-05	0.001051216
Treatment & Medium: Day 3	ENSG00000132510	KDM6B	23135	-0.53	3.94E-05	0.001052711
Treatment & Medium: Day 3	ENSG00000099953	MMP11	4320	-1.13	3.95E-05	0.001054767
Treatment & Medium: Day 3	ENSG00000206341	HLA-H	3136	0.14	3.99E-05	0.001064328
Treatment & Medium: Day 3	ENSG00000187840	EIF4EBP1	1978	0.58	4.03E-05	0.001070417
Treatment & Medium: Day 3	ENSG00000148175	STOM	2040	-0.31	4.03E-05	0.001070417
Treatment & Medium: Day 3	ENSG00000135744	AGT	183	-2.39	4.13E-05	0.001092573
Treatment & Medium: Day 3	ENSG00000125384	PTGER2	5732	0.38	4.13E-05	0.001092573

Treatment & Medium: Day 3	ENSG00000148842	CNNM2	54805	-0.77	4.13E-05	0.001092573
Treatment & Medium: Day 3	ENSG00000132635	PCED1A	64773	-0.23	4.16E-05	0.001097372
Treatment & Medium: Day 3	ENSG00000109790	KLHL5	51088	-0.39	4.17E-05	0.001097372
Treatment & Medium: Day 3	ENSG00000105696	TMEM59L	25789	-1.35	4.17E-05	0.001097372
Treatment & Medium: Day 3	ENSG00000174804	FZD4	8322	-1.27	4.22E-05	0.001108565
Treatment & Medium: Day 3	ENSG00000138182	KIF20B	9585	0.69	4.24E-05	0.001111311
Treatment & Medium: Day 3	ENSG00000121671	CRY2	1408	-0.73	4.27E-05	0.001115432
Treatment & Medium: Day 3	ENSG00000123213	NLN	57486	0.32	4.34E-05	0.001131433
Treatment & Medium: Day 3	ENSG00000196730	DAPK1	1612	-0.16	4.34E-05	0.001131433
Treatment & Medium: Day 3	ENSG00000118257	NRP2	8828	0.88	4.36E-05	0.001132284
Treatment & Medium: Day 3	ENSG00000170153	RNF150	57484	-0.76	4.36E-05	0.001132284
Treatment & Medium: Day 3	ENSG00000087076	HSD17B14	51171	-0.69	4.37E-05	0.001132284
Treatment & Medium: Day 3	ENSG00000119698	PPP4R4	57718	0.89	4.38E-05	0.001134882
Treatment & Medium: Day 3	ENSG00000143322	ABL2	27	0.17	4.39E-05	0.001134882
Treatment & Medium: Day 3	ENSG00000003989	SLC7A2	6542	1.98	4.40E-05	0.001134882
Treatment & Medium: Day 3	ENSG00000143067	ZNF697	90874	0.06	4.43E-05	0.00114054
Treatment & Medium: Day 3	ENSG00000122121	XPNPEP2	7512	-1.00	4.45E-05	0.001144823
Treatment & Medium: Day 3	ENSG00000105281	SLC1A5	6510	-0.24	4.50E-05	0.001153957
Treatment & Medium: Day 3	ENSG00000127951	FGL2	10875	-2.22	4.52E-05	0.0011574
Treatment & Medium: Day 3	ENSG00000134201	GSTM5	2949	-0.32	4.56E-05	0.001166603
Treatment & Medium: Day 3	ENSG00000136040	PLXNC1	10154	-1.69	4.57E-05	0.001168419
Treatment & Medium: Day 3	ENSG00000183775	KCTD16	57528	-0.67	4.64E-05	0.001181906
Treatment & Medium: Day 3	ENSG00000196584	XRCC2	7516	0.79	4.66E-05	0.001185021

Treatment & Medium: Day 3	ENSG00000166833	NAV2	89797	0.63	4.70E-05	0.001194778
Treatment & Medium: Day 3	ENSG00000183160	TMEM119	338773	-0.73	4.75E-05	0.001203809
Treatment & Medium: Day 3	ENSG00000158258	CLSTN2	64084	-1.04	4.75E-05	0.001203809
Treatment & Medium: Day 3	ENSG00000105518	TMEM205	374882	0.42	4.77E-05	0.00120566
Treatment & Medium: Day 3	ENSG00000147872	PLIN2	123	0.37	4.78E-05	0.001207482
Treatment & Medium: Day 3	ENSG00000101665	SMAD7	4092	-1.05	4.83E-05	0.001216158
Treatment & Medium: Day 3	ENSG00000073111	MCM2	4171	0.57	4.85E-05	0.00121796
Treatment & Medium: Day 3	ENSG00000165804	ZNF219	51222	0.52	4.85E-05	0.00121796
Treatment & Medium: Day 3	ENSG00000070081	NUCB2	4925	-0.05	4.96E-05	0.001244927
Treatment & Medium: Day 3	ENSG00000104177	MYEF2	50804	0.23	4.99E-05	0.001249236
Treatment & Medium: Day 3	ENSG00000150938	CRIM1	51232	0.07	5.00E-05	0.001249936
Treatment & Medium: Day 3	ENSG00000070214	SLC44A1	23446	0.36	5.03E-05	0.001254289
Treatment & Medium: Day 3	ENSG00000217236	SP9	100131390	-0.73	5.06E-05	0.0012604
Treatment & Medium: Day 3	ENSG00000072195	SPEG	10290	-0.55	5.08E-05	0.00126383
Treatment & Medium: Day 3	ENSG00000122729	ACO1	48	0.31	5.10E-05	0.001266388
Treatment & Medium: Day 3	ENSG00000166710	B2M	567	0.20	5.13E-05	0.001272023
Treatment & Medium: Day 3	ENSG00000164283	ESM1	11082	-0.79	5.14E-05	0.001272698
Treatment & Medium: Day 3	ENSG00000134531	EMP1	2012	0.29	5.15E-05	0.001272982
Treatment & Medium: Day 3	ENSG00000228716	DHFR	1719	0.58	5.16E-05	0.001274035
Treatment & Medium: Day 3	ENSG00000169604	ANTXR1	84168	-0.61	5.19E-05	0.001277945
Treatment & Medium: Day 3	ENSG00000059377	TBXAS1	6916	0.62	5.20E-05	0.001278308
Treatment & Medium: Day 3	ENSG00000177169	ULK1	8408	-0.47	5.23E-05	0.001282359
Treatment & Medium: Day 3	ENSG00000100592	DAAM1	23002	-0.52	5.23E-05	0.001282359

Treatment & Medium: Day 3	ENSG00000137310	TCF19	6941	0.42	5.25E-05	0.00128403
Treatment & Medium: Day 3	ENSG00000165124	SVEP1	79987	-0.15	5.26E-05	0.00128403
Treatment & Medium: Day 3	ENSG00000135269	TES	26136	-0.10	5.27E-05	0.00128493
Treatment & Medium: Day 3	ENSG00000151725	CENPU	79682	0.56	5.29E-05	0.001287575
Treatment & Medium: Day 3	ENSG00000157445	CACNA2D3	55799	-0.96	5.32E-05	0.001291601
Treatment & Medium: Day 3	ENSG00000242221	PSG2	5670	-1.07	5.33E-05	0.001291601
Treatment & Medium: Day 3	ENSG00000113119	TMCO6	55374	0.40	5.33E-05	0.001291601
Treatment & Medium: Day 3	ENSG00000144369	FAM171B	165215	-1.09	5.35E-05	0.001294338
Treatment & Medium: Day 3	ENSG00000135709	KIAA0513	9764	-0.60	5.37E-05	0.001296961
Treatment & Medium: Day 3	ENSG00000161267	BDH1	622	0.61	5.38E-05	0.001296961
Treatment & Medium: Day 3	ENSG00000182175	RGMA	56963	-0.35	5.39E-05	0.001297322
Treatment & Medium: Day 3	ENSG00000182568	SATB1	6304	-0.69	5.40E-05	0.001298925
Treatment & Medium: Day 3	ENSG00000177570	SAMD12	401474	-1.49	5.47E-05	0.001311986
Treatment & Medium: Day 3	ENSG00000126787	DLGAP5	9787	0.86	5.47E-05	0.001311986
Treatment & Medium: Day 3	ENSG00000146409	SLC18B1	116843	-0.76	5.55E-05	0.00132711
Treatment & Medium: Day 3	ENSG00000100342	APOL1	8542	-0.37	5.55E-05	0.00132711
Treatment & Medium: Day 3	ENSG00000188070	C11orf95	65998	-0.75	5.59E-05	0.001332137
Treatment & Medium: Day 3	ENSG00000174939	ASPHD1	253982	0.85	5.59E-05	0.001332137
Treatment & Medium: Day 3	ENSG00000161570	CCL5	6352	1.50	5.60E-05	0.001332244
Treatment & Medium: Day 3	ENSG00000131323	TRAF3	7187	0.19	5.70E-05	0.001353497
Treatment & Medium: Day 3	ENSG00000184220	CMSS1	84319	0.56	5.71E-05	0.001354813
Treatment & Medium: Day 3	ENSG00000169851	PCDH7	5099	-1.52	5.75E-05	0.001362427
Treatment & Medium: Day 3	ENSG00000241399	CD302	9936	-0.59	5.88E-05	0.001389335

Treatment & Medium: Day 3	ENSG00000048740	CELF2	10659	-0.57	5.98E-05	0.001409332
Treatment & Medium: Day 3	ENSG00000136367	ZFHX2	85446	-0.93	5.98E-05	0.001409332
Treatment & Medium: Day 3	ENSG00000165633	VSTM4	196740	-0.95	6.01E-05	0.001412866
Treatment & Medium: Day 3	ENSG00000143466	IKBKE	9641	0.29	6.01E-05	0.001412866
Treatment & Medium: Day 3	ENSG00000136997	MYC	4609	0.98	6.06E-05	0.001418281
Treatment & Medium: Day 3	ENSG00000025708	TYMP	1890	0.51	6.06E-05	0.001418281
Treatment & Medium: Day 3	ENSG00000100364	KIAA0930	23313	-0.40	6.10E-05	0.001425417
Treatment & Medium: Day 3	ENSG00000167191	GPRC5B	51704	-0.88	6.11E-05	0.001425693
Treatment & Medium: Day 3	ENSG00000176890	TYMS	7298	0.84	6.14E-05	0.001429516
Treatment & Medium: Day 3	ENSG00000171552	BCL2L1	598	0.55	6.14E-05	0.001429516
Treatment & Medium: Day 3	ENSG00000122042	UBL3	5412	-0.63	6.19E-05	0.001438483
Treatment & Medium: Day 3	ENSG00000243244	STON1	11037	-0.49	6.22E-05	0.00144249
Treatment & Medium: Day 3	ENSG00000065882	TBC1D1	23216	0.38	6.23E-05	0.00144249
Treatment & Medium: Day 3	ENSG00000121281	ADCY7	113	-0.49	6.23E-05	0.00144249
Treatment & Medium: Day 3	ENSG00000017427	IGF1	3479	-2.20	6.25E-05	0.001443879
Treatment & Medium: Day 3	ENSG00000172638	EFEMP2	30008	-0.12	6.26E-05	0.001444098
Treatment & Medium: Day 3	ENSG00000010404	IDS	3423	-0.54	6.27E-05	0.001444642
Treatment & Medium: Day 3	ENSG00000134042	MRO	83876	0.72	6.31E-05	0.001451661
Treatment & Medium: Day 3	ENSG00000122507	BBS9	27241	-0.59	6.38E-05	0.001466104
Treatment & Medium: Day 3	ENSG00000185585	OLFML2A	169611	-0.09	6.41E-05	0.001470732
Treatment & Medium: Day 3	ENSG00000253731	PCDHGA6	56109	-0.85	6.46E-05	0.001479379
Treatment & Medium: Day 3	ENSG00000070404	FSTL3	10272	0.67	6.55E-05	0.001496369
Treatment & Medium: Day 3	ENSG00000205795	CYS1	192668	-0.98	6.58E-05	0.001501078

Treatment & Medium: Day 3	ENSG00000134690	CDCA8	55143	0.64	6.60E-05	0.001501225
Treatment & Medium: Day 3	ENSG00000159259	CHAF1B	8208	0.38	6.60E-05	0.001501225
Treatment & Medium: Day 3	ENSG00000128050	PAICS	10606	0.56	6.61E-05	0.001501225
Treatment & Medium: Day 3	ENSG00000069869	NEDD4	4734	0.52	6.65E-05	0.001508793
Treatment & Medium: Day 3	ENSG00000111674	ENO2	2026	-0.33	6.74E-05	0.001527562
Treatment & Medium: Day 3	ENSG00000125977	EIF2S2	8894	0.38	6.76E-05	0.001528919
Treatment & Medium: Day 3	ENSG00000181754	AMIGO1	57463	-0.87	6.81E-05	0.001536872
Treatment & Medium: Day 3	ENSG00000139567	ACVRL1	94	0.19	6.81E-05	0.001536872
Treatment & Medium: Day 3	ENSG00000119938	PPP1R3C	5507	-1.36	6.86E-05	0.001543679
Treatment & Medium: Day 3	ENSG00000168078	PBK	55872	0.79	6.86E-05	0.001543679
Treatment & Medium: Day 3	ENSG00000255717	SNHG1	23642	0.53	6.96E-05	0.001563511
Treatment & Medium: Day 3	ENSG00000163827	LRRC2	79442	-0.38	6.99E-05	0.00156767
Treatment & Medium: Day 3	ENSG00000141447	OSBPL1A	114876	-0.64	7.02E-05	0.00157193
Treatment & Medium: Day 3	ENSG00000182240	BACE2	25825	-0.04	7.06E-05	0.001577242
Treatment & Medium: Day 3	ENSG00000059378	PARP12	64761	0.31	7.09E-05	0.001583457
Treatment & Medium: Day 3	ENSG00000057019	DCBLD2	131566	0.19	7.13E-05	0.001589382
Treatment & Medium: Day 3	ENSG00000143434	SEMA6C	10500	-0.41	7.19E-05	0.00159947
Treatment & Medium: Day 3	ENSG00000131747	TOP2A	7153	0.63	7.25E-05	0.001612021
Treatment & Medium: Day 3	ENSG00000105088	OLFM2	93145	-2.86	7.32E-05	0.001624042
Treatment & Medium: Day 3	ENSG00000243649	CFB	629	0.18	7.34E-05	0.001626699
Treatment & Medium: Day 3	ENSG00000213722	DDAH2	23564	-0.41	7.36E-05	0.00162799
Treatment & Medium: Day 3	ENSG00000197977	ELOVL2	54898	-0.48	7.38E-05	0.001629258
Treatment & Medium: Day 3	ENSG00000141837	CACNA1A	773	-0.04	7.38E-05	0.001629258

Treatment & Medium: Day 3	ENSG00000132423	COQ3	51805	0.70	7.41E-05	0.001632023
Treatment & Medium: Day 3	ENSG00000130702	LAMA5	3911	-0.31	7.46E-05	0.001641641
Treatment & Medium: Day 3	ENSG00000182492	BGN	633	-0.55	7.53E-05	0.001652131
Treatment & Medium: Day 3	ENSG00000115041	KCNIP3	30818	0.85	7.53E-05	0.001652131
Treatment & Medium: Day 3	ENSG00000125885	MCM8	84515	0.35	7.56E-05	0.001655789
Treatment & Medium: Day 3	ENSG00000140937	CDH11	1009	-0.52	7.59E-05	0.001660157
Treatment & Medium: Day 3	ENSG00000136542	GALNT5	11227	0.01	7.66E-05	0.001673026
Treatment & Medium: Day 3	ENSG00000168079	SCARA5	286133	-0.17	7.68E-05	0.001674139
Treatment & Medium: Day 3	ENSG00000136235	GPNMB	10457	-1.17	7.69E-05	0.001674139
Treatment & Medium: Day 3	ENSG00000105185	PDCD5	9141	0.74	7.78E-05	0.001691586
Treatment & Medium: Day 3	ENSG00000085276	MECOM	2122	-0.39	7.80E-05	0.001691586
Treatment & Medium: Day 3	ENSG00000104870	FCGRT	2217	-0.38	7.80E-05	0.001691586
Treatment & Medium: Day 3	ENSG00000157456	CCNB2	9133	0.84	7.85E-05	0.001698107
Treatment & Medium: Day 3	ENSG00000151474	FRMD4A	55691	0.32	7.85E-05	0.001698107
Treatment & Medium: Day 3	ENSG00000146122	DAAM2	23500	-0.40	7.86E-05	0.001698107
Treatment & Medium: Day 3	ENSG00000143013	LMO4	8543	-0.05	7.88E-05	0.001698756
Treatment & Medium: Day 3	ENSG00000072952	MRVI1	10335	-0.94	7.95E-05	0.001712241
Treatment & Medium: Day 3	ENSG00000100968	NFATC4	4776	-0.58	7.96E-05	0.001712854
Treatment & Medium: Day 3	ENSG00000148082	SHC3	53358	0.14	8.03E-05	0.001723013
Treatment & Medium: Day 3	ENSG00000060982	BCAT1	586	-0.16	8.03E-05	0.001723013
Treatment & Medium: Day 3	ENSG00000197208	SLC22A4	6583	0.39	8.05E-05	0.00172425
Treatment & Medium: Day 3	ENSG00000159399	HK2	3099	-0.55	8.12E-05	0.001735578
Treatment & Medium: Day 3	ENSG00000108561	C1QBP	708	0.55	8.13E-05	0.001735697

Treatment & Medium: Day 3	ENSG00000204634	TBC1D8	11138	0.64	8.16E-05	0.00173926
Treatment & Medium: Day 3	ENSG00000164176	EDIL3	10085	-1.06	8.17E-05	0.00173926
Treatment & Medium: Day 3	ENSG00000162512	SDC3	9672	-0.63	8.18E-05	0.001739673
Treatment & Medium: Day 3	ENSG00000162852	CNST	163882	-0.30	8.23E-05	0.001747404
Treatment & Medium: Day 3	ENSG00000130508	PXDN	7837	0.26	8.26E-05	0.001751555
Treatment & Medium: Day 3	ENSG00000112312	GMNN	51053	0.55	8.30E-05	0.001756686
Treatment & Medium: Day 3	ENSG00000213366	GSTM2	2946	-0.55	8.34E-05	0.00176271
Treatment & Medium: Day 3	ENSG00000137103	TMEM8B	51754	-0.70	8.39E-05	0.001769749
Treatment & Medium: Day 3	ENSG00000181163	NPM1	4869	0.51	8.39E-05	0.001769749
Treatment & Medium: Day 3	ENSG00000132481	TRIM47	91107	0.04	8.44E-05	0.001777475
Treatment & Medium: Day 3	ENSG00000124145	SDC4	6385	-0.33	8.45E-05	0.001777475
Treatment & Medium: Day 3	ENSG00000116774	OLFML3	56944	-0.93	8.51E-05	0.001786915
Treatment & Medium: Day 3	ENSG00000144810	COL8A1	1295	0.61	8.54E-05	0.001786915
Treatment & Medium: Day 3	ENSG00000169271	HSPB3	8988	0.64	8.55E-05	0.001786915
Treatment & Medium: Day 3	ENSG00000073910	FRY	10129	-0.54	8.55E-05	0.001786915
Medium: Day 1	ENSG00000105825	TFPI2	7980	1.98	5.85E-14	9.06E-10
Medium: Day 1	ENSG00000163735	CXCL5	6374	3.86	5.63E-12	4.36E-08
Medium: Day 1	ENSG00000163131	CTSS	1520	2.28	8.82E-12	4.56E-08
Medium: Day 1	ENSG00000198074	AKR1B10	57016	-3.02	1.24E-11	4.80E-08
Medium: Day 1	ENSG00000186417	GLDN	342035	-1.59	2.27E-11	7.03E-08
Medium: Day 1	ENSG00000006118	TMEM132A	54972	1.75	3.56E-11	9.19E-08
Medium: Day 1	ENSG00000134363	FST	10468	1.09	1.83E-10	3.74E-07
Medium: Day 1	ENSG00000117594	HSD11B1	3290	2.39	1.93E-10	3.74E-07
Medium: Day 1	ENSG00000163734	CXCL3	2921	1.84	3.82E-10	6.52E-07
Medium: Day 1	ENSG00000143819	EPHX1	2052	-0.94	4.21E-10	6.52E-07
Medium: Day 1	ENSG00000041982	TNC	3371	1.51	5.47E-10	7.71E-07
Medium: Day 1	ENSG00000085117	CD82	3732	1.38	6.36E-10	8.21E-07
Medium: Day 1	ENSG00000108602	ALDH3A1	218	-1.69	1.02E-09	1.17E-06
Medium: Day 1	ENSG00000091490	SEL1L3	23231	-1.39	1.11E-09	1.17E-06

Medium: Day 1	ENSG00000185565	LSAMP	4045	-1.06	1.14E-09	1.17E-06
Medium: Day 1	ENSG00000120498	TEX11	56159	2.94	1.39E-09	1.35E-06
Medium: Day 1	ENSG00000161570	CCL5	6352	4.01	1.72E-09	1.52E-06
Medium: Day 1	ENSG00000196139	AKR1C3	8644	-1.11	1.77E-09	1.52E-06
Medium: Day 1	ENSG00000010932	FMO1	2326	-3.94	2.05E-09	1.67E-06
Medium: Day 1	ENSG00000104321	TRPA1	8989	1.73	2.17E-09	1.68E-06
Medium: Day 1	ENSG00000110031	LPXN	9404	0.99	2.91E-09	2.03E-06
Medium: Day 1	ENSG00000087842	PIR	8544	-0.84	3.11E-09	2.03E-06
Medium: Day 1	ENSG00000140961	OSGIN1	29948	-0.90	3.12E-09	2.03E-06
Medium: Day 1	ENSG00000172197	MBOAT1	154141	-0.94	3.14E-09	2.03E-06
Medium: Day 1	ENSG00000187957	DNER	92737	2.43	3.81E-09	2.36E-06
Medium: Day 1	ENSG00000169239	CA5B	11238	-0.86	4.11E-09	2.45E-06
Medium: Day 1	ENSG00000081041	CXCL2	2920	1.83	4.36E-09	2.47E-06
Medium: Day 1	ENSG00000118113	MMP8	4317	3.35	4.46E-09	2.47E-06
Medium: Day 1	ENSG00000164309	CMYA5	202333	-1.11	5.19E-09	2.77E-06
Medium: Day 1	ENSG00000163739	CXCL1	2919	1.44	5.84E-09	3.01E-06
Medium: Day 1	ENSG00000141052	MYOCD	93649	2.25	6.32E-09	3.16E-06
Medium: Day 1	ENSG00000108688	CCL7	6354	1.98	7.80E-09	3.74E-06
Medium: Day 1	ENSG00000174804	FZD4	8322	-0.83	8.01E-09	3.74E-06
Medium: Day 1	ENSG00000166920	C15orf48	84419	2.28	8.20E-09	3.74E-06
Medium: Day 1	ENSG00000087086	FTL	2512	-0.98	8.54E-09	3.77E-06
Medium: Day 1	ENSG00000120899	PTK2B	2185	-1.82	8.76E-09	3.77E-06
Medium: Day 1	ENSG00000197632	SERPINB2	5055	2.90	9.45E-09	3.92E-06
Medium: Day 1	ENSG00000168079	SCARA5	286133	1.53	9.82E-09	3.92E-06
Medium: Day 1	ENSG00000124875	CXCL6	6372	2.18	9.86E-09	3.92E-06
Medium: Day 1	ENSG00000197208	SLC22A4	6583	0.83	1.03E-08	3.97E-06
Medium: Day 1	ENSG00000135373	EHF	26298	3.74	1.05E-08	3.97E-06
Medium: Day 1	ENSG00000145819	ARHGAP26	23092	-1.47	1.10E-08	4.00E-06
Medium: Day 1	ENSG00000173114	LRRN3	54674	1.67	1.13E-08	4.00E-06
Medium: Day 1	ENSG00000138449	SLC40A1	30061	-1.39	1.14E-08	4.00E-06
Medium: Day 1	ENSG00000139209	SLC38A4	55089	-1.04	1.17E-08	4.03E-06
Medium: Day 1	ENSG00000029153	ARNTL2	56938	0.78	1.21E-08	4.08E-06
Medium: Day 1	ENSG00000006062	MAP3K14	9020	-0.85	1.32E-08	4.30E-06
Medium: Day 1	ENSG00000168955	TM4SF20	79853	-1.46	1.35E-08	4.30E-06
Medium: Day 1	ENSG00000008517	IL32	9235	3.06	1.36E-08	4.30E-06
Medium: Day 1	ENSG00000113645	WWC1	23286	1.43	1.47E-08	4.56E-06
Medium: Day 1	ENSG00000165092	ALDH1A1	216	-2.67	1.70E-08	5.17E-06
Medium: Day 1	ENSG00000196878	LAMB3	3914	0.98	1.78E-08	5.29E-06
Medium: Day 1	ENSG00000150630	VEGFC	7424	0.81	1.93E-08	5.57E-06
Medium: Day 1	ENSG00000197646	PDCD1LG2	80380	0.83	1.94E-08	5.57E-06
Medium: Day 1	ENSG00000170891	CYTL1	54360	-0.98	2.12E-08	5.98E-06
Medium: Day 1	ENSG00000019582	CD74	972	1.79	2.24E-08	6.19E-06

Medium: Day 1	ENSG00000108797	CNTNAP1	8506	0.91	2.49E-08	6.78E-06
Medium: Day 1	ENSG00000159674	SPON2	10417	-1.18	2.58E-08	6.79E-06
Medium: Day 1	ENSG00000100739	BDKRB1	623	1.27	2.58E-08	6.79E-06
Medium: Day 1	ENSG00000173918	C1QTNF1	114897	1.10	2.79E-08	7.22E-06
Medium: Day 1	ENSG00000134321	RSAD2	91543	2.94	3.03E-08	7.68E-06
Medium: Day 1	ENSG00000050344	NFE2L3	9603	1.55	3.12E-08	7.79E-06
Medium: Day 1	ENSG00000085662	AKR1B1	231	1.27	3.68E-08	9.04E-06
Medium: Day 1	ENSG00000184374	COLEC10	10584	-1.23	3.89E-08	9.41E-06
Medium: Day 1	ENSG00000146411	SLC2A12	154091	-1.60	4.02E-08	9.59E-06
Medium: Day 1	ENSG00000142657	PGD	5226	-0.58	4.48E-08	1.05E-05
Medium: Day 1	ENSG00000072210	ALDH3A2	224	-0.86	4.85E-08	1.12E-05
Medium: Day 1	ENSG00000164171	ITGA2	3673	0.66	4.91E-08	1.12E-05
Medium: Day 1	ENSG00000183762	KREMEN1	83999	-0.63	5.21E-08	1.17E-05
Medium: Day 1	ENSG00000100504	PYGL	5836	-0.62	5.63E-08	1.22E-05
Medium: Day 1	ENSG00000136244	IL6	3569	2.01	5.66E-08	1.22E-05
Medium: Day 1	ENSG00000108448	TRIM16L	147166	-0.73	5.69E-08	1.22E-05
Medium: Day 1	ENSG00000234745	HLA-B	3106	1.00	6.66E-08	1.41E-05
Medium: Day 1	ENSG00000135114	OASL	8638	2.12	6.87E-08	1.44E-05
Medium: Day 1	ENSG00000181019	NQO1	1728	-0.69	7.43E-08	1.51E-05
Medium: Day 1	ENSG00000144959	NCEH1	57552	0.84	7.53E-08	1.51E-05
Medium: Day 1	ENSG00000163347	CLDN1	9076	3.06	7.56E-08	1.51E-05
Medium: Day 1	ENSG00000118503	TNFAIP3	7128	2.09	7.67E-08	1.51E-05
Medium: Day 1	ENSG00000105894	PTN	5764	-0.59	7.70E-08	1.51E-05
Medium: Day 1	ENSG00000149968	MMP3	4314	2.26	8.20E-08	1.59E-05
Medium: Day 1	ENSG00000156427	FGF18	8817	-1.92	8.41E-08	1.59E-05
Medium: Day 1	ENSG00000131389	SLC6A6	6533	-0.57	8.43E-08	1.59E-05
Medium: Day 1	ENSG00000146233	CYP39A1	51302	-1.29	8.75E-08	1.63E-05
Medium: Day 1	ENSG00000124749	COL21A1	81578	-2.15	9.45E-08	1.74E-05
Medium: Day 1	ENSG00000164776	PHKG1	5260	-2.05	9.67E-08	1.76E-05
Medium: Day 1	ENSG00000117643	MAN1C1	57134	-0.92	9.84E-08	1.77E-05
Medium: Day 1	ENSG00000004399	PLXND1	23129	-0.64	1.02E-07	1.82E-05
Medium: Day 1	ENSG00000149571	KIRREL3	84623	1.04	1.10E-07	1.93E-05
Medium: Day 1	ENSG00000154654	NCAM2	4685	-0.80	1.12E-07	1.94E-05
Medium: Day 1	ENSG00000241749	RPSAP52	204010	0.95	1.13E-07	1.94E-05
Medium: Day 1	ENSG00000122035	RASL11A	387496	-0.93	1.22E-07	2.07E-05
Medium: Day 1	ENSG00000179104	TMTC2	160335	-1.01	1.39E-07	2.34E-05
Medium: Day 1	ENSG00000133216	EPHB2	2048	-0.87	1.43E-07	2.37E-05
Medium: Day 1	ENSG00000108691	CCL2	6347	1.36	1.44E-07	2.37E-05
Medium: Day 1	ENSG00000146232	NFKBIE	4794	1.13	1.46E-07	2.38E-05
Medium: Day 1	ENSG00000152217	SETBP1	26040	-1.08	1.53E-07	2.44E-05
Medium: Day 1	ENSG00000132000	PODNL1	79883	-0.95	1.53E-07	2.44E-05
Medium: Day 1	ENSG00000107984	DKK1	22943	0.77	1.58E-07	2.49E-05

Medium: Day 1	ENSG00000127951	FGL2	10875	-1.51	1.67E-07	2.58E-05
Medium: Day 1	ENSG00000175538	KCNE3	10008	-1.64	1.67E-07	2.58E-05
Medium: Day 1	ENSG00000241644	INMT	11185	-1.07	1.68E-07	2.58E-05
Medium: Day 1	ENSG00000165272	AQP3	360	-1.16	1.73E-07	2.63E-05
Medium: Day 1	ENSG00000141404	GNAL	2774	-1.81	1.80E-07	2.71E-05
Medium: Day 1	ENSG00000106366	SERPINE1	5054	0.78	1.91E-07	2.85E-05
Medium: Day 1	ENSG00000185920	PTCH1	5727	-0.92	2.14E-07	3.14E-05
Medium: Day 1	ENSG00000120708	TGFBI	7045	-0.68	2.15E-07	3.14E-05
Medium: Day 1	ENSG00000103710	RASL12	51285	-1.39	2.21E-07	3.20E-05
Medium: Day 1	ENSG00000100911	PSME2	5721	0.70	2.24E-07	3.21E-05
Medium: Day 1	ENSG00000122547	EEPD1	80820	-1.00	2.31E-07	3.29E-05
Medium: Day 1	ENSG00000148175	STOM	2040	-0.91	2.37E-07	3.32E-05
Medium: Day 1	ENSG00000188641	DPYD	1806	-0.57	2.38E-07	3.32E-05
Medium: Day 1	ENSG00000120693	SMAD9	4093	-1.23	2.41E-07	3.33E-05
Medium: Day 1	ENSG00000169245	CXCL10	3627	6.17	2.47E-07	3.35E-05
Medium: Day 1	ENSG00000160712	IL6R	3570	-0.69	2.47E-07	3.35E-05
Medium: Day 1	ENSG00000152953	STK32B	55351	-0.60	2.48E-07	3.35E-05
Medium: Day 1	ENSG00000233452	STXBP5-AS1	729178	1.08	2.53E-07	3.38E-05
Medium: Day 1	ENSG00000166448	TMEM130	222865	-0.99	2.60E-07	3.43E-05
Medium: Day 1	ENSG00000197506	SLC28A3	64078	1.95	2.61E-07	3.43E-05
Medium: Day 1	ENSG00000197496	SLC2A10	81031	-0.69	2.79E-07	3.63E-05
Medium: Day 1	ENSG00000164112	TMEM155	132332	-1.01	2.81E-07	3.63E-05
Medium: Day 1	ENSG00000198542	ITGBL1	9358	-0.48	2.98E-07	3.81E-05
Medium: Day 1	ENSG00000171872	KLF17	128209	1.80	3.15E-07	4.00E-05
Medium: Day 1	ENSG00000167202	TBC1D2B	23102	-0.56	3.26E-07	4.08E-05
Medium: Day 1	ENSG00000023445	BIRC3	330	2.27	3.26E-07	4.08E-05
Medium: Day 1	ENSG00000135218	CD36	948	-1.78	3.38E-07	4.19E-05
Medium: Day 1	ENSG00000134201	GSTM5	2949	-0.93	3.46E-07	4.25E-05
Medium: Day 1	ENSG00000231925	TAPBP	6892	0.76	3.55E-07	4.33E-05
Medium: Day 1	ENSG00000174600	CMKLR1	1240	-1.01	3.76E-07	4.55E-05
Medium: Day 1	ENSG00000107562	CXCL12	6387	-1.30	3.97E-07	4.77E-05
Medium: Day 1	ENSG00000055163	CYFIP2	26999	0.72	4.42E-07	5.14E-05
Medium: Day 1	ENSG00000104081	BMF	90427	-1.69	4.42E-07	5.14E-05
Medium: Day 1	ENSG00000105246	EBI3	10148	3.22	4.43E-07	5.14E-05
Medium: Day 1	ENSG00000106772	PRUNE2	158471	-0.83	4.45E-07	5.14E-05
Medium: Day 1	ENSG00000137869	CYP19A1	1588	-1.40	4.48E-07	5.14E-05
Medium: Day 1	ENSG00000175899	A2M	2	-1.56	4.51E-07	5.14E-05
Medium: Day 1	ENSG00000225783	MIAT	440823	-1.64	4.51E-07	5.14E-05
Medium: Day 1	ENSG00000105245	NUMBL	9253	-0.49	4.55E-07	5.15E-05
Medium: Day 1	ENSG00000134070	IRAK2	3656	0.97	4.61E-07	5.16E-05
Medium: Day 1	ENSG00000108639	SYNGR2	9144	0.70	4.66E-07	5.16E-05
Medium: Day 1	ENSG00000187800	PEAR1	375033	-0.70	4.69E-07	5.16E-05

Medium: Day 1	ENSG00000128342	LIF	3976	1.61	4.70E-07	5.16E-05
Medium: Day 1	ENSG00000071575	TRIB2	28951	-0.74	4.78E-07	5.21E-05
Medium: Day 1	ENSG00000129667	RHBDF2	79651	1.00	5.03E-07	5.45E-05
Medium: Day 1	ENSG00000213366	GSTM2	2946	-0.83	5.15E-07	5.54E-05
Medium: Day 1	ENSG00000173559	NABP1	64859	-0.58	5.20E-07	5.56E-05
Medium: Day 1	ENSG00000147251	DOCK11	139818	-0.72	5.44E-07	5.78E-05
Medium: Day 1	ENSG00000165633	VSTM4	196740	-0.91	5.74E-07	6.05E-05
Medium: Day 1	ENSG00000077150	NFKB2	4791	0.91	6.01E-07	6.29E-05
Medium: Day 1	ENSG00000092010	PSME1	5720	0.55	6.26E-07	6.51E-05
Medium: Day 1	ENSG00000133026	MYH10	4628	-0.51	6.60E-07	6.82E-05
Medium: Day 1	ENSG00000048540	LMO3	55885	-1.85	6.74E-07	6.89E-05
Medium: Day 1	ENSG00000158023	WDR66	144406	0.89	6.76E-07	6.89E-05
Medium: Day 1	ENSG00000139211	AMIGO2	347902	-1.36	6.87E-07	6.89E-05
Medium: Day 1	ENSG00000188157	AGRN	375790	0.69	6.88E-07	6.89E-05
Medium: Day 1	ENSG00000176658	MYO1D	4642	-0.71	6.89E-07	6.89E-05
Medium: Day 1	ENSG00000116774	OLFML3	56944	-0.70	6.94E-07	6.90E-05
Medium: Day 1	ENSG00000158714	SLAMF8	56833	3.84	7.29E-07	7.20E-05
Medium: Day 1	ENSG00000099998	GGT5	2687	1.25	7.52E-07	7.38E-05
Medium: Day 1	ENSG00000105518	TMEM205	374882	0.69	7.75E-07	7.55E-05
Medium: Day 1	ENSG00000197415	VEPH1	79674	0.57	8.11E-07	7.81E-05
Medium: Day 1	ENSG00000147041	SYTL5	94122	-2.07	8.13E-07	7.81E-05
Medium: Day 1	ENSG00000243244	STON1	11037	-0.81	8.17E-07	7.81E-05
Medium: Day 1	ENSG00000196611	MMP1	4312	0.95	8.60E-07	8.09E-05
Medium: Day 1	ENSG00000166592	RRAD	6236	1.43	8.64E-07	8.09E-05
Medium: Day 1	ENSG00000028277	POU2F2	5452	0.88	8.65E-07	8.09E-05
Medium: Day 1	ENSG00000104856	RELB	5971	0.91	8.67E-07	8.09E-05
Medium: Day 1	ENSG00000072422	RHOBTB1	9886	-0.72	8.87E-07	8.19E-05
Medium: Day 1	ENSG00000105185	PDCD5	9141	0.49	8.88E-07	8.19E-05
Medium: Day 1	ENSG00000154640	BTG3	10950	0.47	9.02E-07	8.27E-05
Medium: Day 1	ENSG00000164237	CMBL	134147	-0.63	9.13E-07	8.32E-05
Medium: Day 1	ENSG00000076685	NT5C2	22978	-0.47	9.67E-07	8.76E-05
Medium: Day 1	ENSG00000133110	POSTN	10631	-1.13	9.76E-07	8.79E-05
Medium: Day 1	ENSG00000138395	CDK15	65061	0.84	1.05E-06	9.44E-05
Medium: Day 1	ENSG00000164484	TMEM200A	114801	-0.64	1.08E-06	9.60E-05
Medium: Day 1	ENSG00000140092	FBLN5	10516	-0.66	1.11E-06	9.82E-05
Medium: Day 1	ENSG00000130303	BST2	684	2.16	1.12E-06	9.82E-05
Medium: Day 1	ENSG00000116285	ERRFI1	54206	-0.77	1.14E-06	9.94E-05
Medium: Day 1	ENSG00000119714	GPR68	8111	1.55	1.16E-06	0.000100588
Medium: Day 1	ENSG00000166562	SEC11C	90701	0.52	1.18E-06	0.000101411
Medium: Day 1	ENSG00000128965	CHAC1	79094	1.04	1.18E-06	0.000101411
Medium: Day 1	ENSG00000170271	FAXDC2	10826	-0.71	1.21E-06	0.000103625
Medium: Day 1	ENSG00000060718	COL11A1	1301	-1.14	1.23E-06	0.000104893

Medium: Day 1	ENSG00000164220	F2RL2	2151	-0.57	1.24E-06	0.000104985
Medium: Day 1	ENSG00000182240	BACE2	25825	0.62	1.25E-06	0.000104985
Medium: Day 1	ENSG00000154175	ABI3BP	25890	1.34	1.26E-06	0.000104985
Medium: Day 1	ENSG00000102287	GABRE	2564	-0.67	1.26E-06	0.000104985
Medium: Day 1	ENSG00000175274	TP53I11	9537	-0.55	1.32E-06	0.000108857
Medium: Day 1	ENSG00000182667	NTM	50863	0.69	1.32E-06	0.000108857
Medium: Day 1	ENSG00000170989	S1PR1	1901	-1.70	1.33E-06	0.000108857
Medium: Day 1	ENSG00000170381	SEMA3E	9723	-0.80	1.42E-06	0.000115728
Medium: Day 1	ENSG00000196730	DAPK1	1612	-0.71	1.45E-06	0.00011779
Medium: Day 1	ENSG00000119138	KLF9	687	-0.65	1.46E-06	0.00011779
Medium: Day 1	ENSG00000112699	GMDS	2762	-0.61	1.48E-06	0.000118551
Medium: Day 1	ENSG00000154645	CHODL	140578	2.68	1.50E-06	0.000119738
Medium: Day 1	ENSG00000148180	GSN	2934	-0.66	1.51E-06	0.00012011
Medium: Day 1	ENSG00000164307	ERAP1	51752	0.72	1.54E-06	0.000121829
Medium: Day 1	ENSG00000136861	CDK5RAP2	55755	-0.40	1.56E-06	0.000122307
Medium: Day 1	ENSG00000134326	CMPK2	129607	2.51	1.62E-06	0.000126351
Medium: Day 1	ENSG00000079931	MOXD1	26002	0.48	1.62E-06	0.000126351
Medium: Day 1	ENSG00000183454	GRIN2A	2903	2.29	1.66E-06	0.000128901
Medium: Day 1	ENSG00000125538	IL1B	3553	4.53	1.74E-06	0.000132585
Medium: Day 1	ENSG00000113361	CDH6	1004	-0.60	1.74E-06	0.000132585
Medium: Day 1	ENSG00000105499	PLA2G4C	8605	1.16	1.74E-06	0.000132585
Medium: Day 1	ENSG00000204642	HLA-F	3134	1.42	1.75E-06	0.000132585
Medium: Day 1	ENSG00000141150	RASL10B	91608	-0.95	1.77E-06	0.000133464
Medium: Day 1	ENSG00000074410	CA12	771	0.65	1.79E-06	0.000134774
Medium: Day 1	ENSG00000091136	LAMB1	3912	-0.55	1.82E-06	0.00013603
Medium: Day 1	ENSG00000135074	ADAM19	8728	-0.54	1.84E-06	0.000136191
Medium: Day 1	ENSG00000164292	RHOBTB3	22836	-0.65	1.84E-06	0.000136191
Medium: Day 1	ENSG00000106780	MEGF9	1955	-0.73	1.85E-06	0.000136352
Medium: Day 1	ENSG00000149212	SESN3	143686	-1.27	1.90E-06	0.000139375
Medium: Day 1	ENSG00000142046	TMEM91	641649	-1.08	1.94E-06	0.000141457
Medium: Day 1	ENSG00000104687	GSR	2936	-0.47	1.95E-06	0.000141643
Medium: Day 1	ENSG00000106123	EPHB6	2051	-0.92	1.96E-06	0.000141643
Medium: Day 1	ENSG00000214688	C10orf105	414152	-1.30	1.99E-06	0.000143587
Medium: Day 1	ENSG00000053254	FOXN3	1112	-0.61	2.03E-06	0.000145408
Medium: Day 1	ENSG00000139974	SLC38A6	145389	-0.63	2.05E-06	0.000146365
Medium: Day 1	ENSG00000204941	PSG5	5673	-0.44	2.07E-06	0.000147193
Medium: Day 1	ENSG00000134202	GSTM3	2947	-0.43	2.28E-06	0.000160993
Medium: Day 1	ENSG00000162804	SNED1	25992	-0.89	2.33E-06	0.000164017
Medium: Day 1	ENSG00000100364	KIAA0930	23313	-0.51	2.35E-06	0.000164828
Medium: Day 1	ENSG00000011105	TSPAN9	10867	-0.56	2.43E-06	0.000169794
Medium: Day 1	ENSG00000072952	MRVI1	10335	-1.06	2.54E-06	0.00017647
Medium: Day 1	ENSG00000152952	PLOD2	5352	0.69	2.66E-06	0.000183719

Medium: Day 1	ENSG00000115828	QPCT	25797	0.59	2.69E-06	0.000185484
Medium: Day 1	ENSG00000147872	PLIN2	123	0.44	2.76E-06	0.000188908
Medium: Day 1	ENSG00000136960	ENPP2	5168	-0.44	2.77E-06	0.000188908
Medium: Day 1	ENSG00000160326	SLC2A6	11182	1.15	2.89E-06	0.000196107
Medium: Day 1	ENSG00000113657	DPYSL3	1809	0.46	2.92E-06	0.000197362
Medium: Day 1	ENSG00000235162	C12orf75	387882	0.43	2.93E-06	0.000197362
Medium: Day 1	ENSG00000104332	SFRP1	6422	-0.81	2.96E-06	0.000198321
Medium: Day 1	ENSG00000169908	TM4SF1	4071	0.97	2.97E-06	0.000198321
Medium: Day 1	ENSG00000241399	CD302	9936	-0.82	3.01E-06	0.00020017
Medium: Day 1	ENSG00000120949	TNFRSF8	943	2.21	3.05E-06	0.000202151
Medium: Day 1	ENSG00000120915	EPHX2	2053	-0.90	3.09E-06	0.000203148
Medium: Day 1	ENSG00000149380	P4HA3	283208	0.78	3.09E-06	0.000203148
Medium: Day 1	ENSG00000145604	SKP2	6502	-0.49	3.14E-06	0.000204731
Medium: Day 1	ENSG00000015475	BID	637	0.51	3.15E-06	0.000204731
Medium: Day 1	ENSG00000062282	DGAT2	84649	1.24	3.16E-06	0.000204731
Medium: Day 1	ENSG00000100906	NFKBIA	4792	0.86	3.20E-06	0.000205473
Medium: Day 1	ENSG00000136717	BIN1	274	-0.51	3.20E-06	0.000205473
Medium: Day 1	ENSG00000137033	IL33	90865	1.84	3.32E-06	0.000212333
Medium: Day 1	ENSG00000118515	SGK1	6446	-0.49	3.39E-06	0.000216029
Medium: Day 1	ENSG00000125257	ABCC4	10257	-0.38	3.40E-06	0.000216029
Medium: Day 1	ENSG00000125378	BMP4	652	-0.96	3.43E-06	0.000216652
Medium: Day 1	ENSG00000197381	ADARB1	104	-0.88	3.48E-06	0.000219336
Medium: Day 1	ENSG00000038427	VCAN	1462	-0.61	3.50E-06	0.000219617
Medium: Day 1	ENSG00000169508	GPR183	1880	3.07	3.65E-06	0.000226509
Medium: Day 1	ENSG00000187134	AKR1C1	1645	-0.79	3.66E-06	0.000226509
Medium: Day 1	ENSG00000074416	MGLL	11343	0.53	3.66E-06	0.000226509
Medium: Day 1	ENSG00000169184	MN1	4330	-0.59	3.68E-06	0.000226728
Medium: Day 1	ENSG00000175745	NR2F1	7025	-0.62	3.69E-06	0.000226728
Medium: Day 1	ENSG00000158292	GPR153	387509	-0.59	3.75E-06	0.000229509
Medium: Day 1	ENSG00000132840	BHMT2	23743	-0.45	3.85E-06	0.000234596
Medium: Day 1	ENSG00000145147	SLIT2	9353	0.47	3.88E-06	0.000235503
Medium: Day 1	ENSG00000165655	ZNF503	84858	-0.74	3.91E-06	0.000236437
Medium: Day 1	ENSG00000177156	TALDO1	6888	-0.41	3.98E-06	0.000240127
Medium: Day 1	ENSG00000197971	MBP	4155	-0.60	4.12E-06	0.000247459
Medium: Day 1	ENSG00000128016	ZFP36	7538	-0.57	4.17E-06	0.000249657
Medium: Day 1	ENSG00000167549	CORO6	84940	-0.96	4.24E-06	0.000252568
Medium: Day 1	ENSG00000013583	HEBP1	50865	-0.35	4.47E-06	0.000265205
Medium: Day 1	ENSG00000143387	CTSK	1513	0.43	4.60E-06	0.000271739
Medium: Day 1	ENSG00000152580	IGSF10	285313	-1.36	4.64E-06	0.000273458
Medium: Day 1	ENSG00000101236	RNF24	11237	-0.39	4.68E-06	0.000274833
Medium: Day 1	ENSG00000168405	CMAHP	8418	0.58	4.77E-06	0.000279131
Medium: Day 1	ENSG00000166863	TAC3	6866	2.71	4.81E-06	0.000279337

Medium: Day 1	ENSG00000115252	PDE1A	5136	-1.82	4.85E-06	0.000279337
Medium: Day 1	ENSG00000138185	ENTPD1	953	-0.94	4.85E-06	0.000279337
Medium: Day 1	ENSG00000155511	GRIA1	2890	-0.84	4.85E-06	0.000279337
Medium: Day 1	ENSG00000137210	TMEM14B	81853	0.46	4.90E-06	0.000281195
Medium: Day 1	ENSG00000239474	KLHL41	10324	-1.57	4.99E-06	0.000285493
Medium: Day 1	ENSG00000185432	METTL7A	25840	-0.64	5.01E-06	0.000285545
Medium: Day 1	ENSG00000182568	SATB1	6304	-0.76	5.09E-06	0.000289084
Medium: Day 1	ENSG00000138646	HERC5	51191	1.88	5.16E-06	0.000291389
Medium: Day 1	ENSG00000147408	CSGALNACT1	55790	0.44	5.17E-06	0.000291389
Medium: Day 1	ENSG00000166710	B2M	567	0.55	5.24E-06	0.000293607
Medium: Day 1	ENSG00000211445	GPX3	2878	-1.52	5.25E-06	0.000293607
Medium: Day 1	ENSG00000115380	EFEMP1	2202	-0.78	5.40E-06	0.000300427
Medium: Day 1	ENSG00000162595	DIRAS3	9077	-1.22	5.41E-06	0.000300427
Medium: Day 1	ENSG00000046653	GPM6B	2824	0.58	5.50E-06	0.000304561
Medium: Day 1	ENSG00000171155	C1GALT1C1	29071	0.40	5.54E-06	0.000305345
Medium: Day 1	ENSG00000115963	RND3	390	0.42	5.62E-06	0.000307835
Medium: Day 1	ENSG00000067182	TNFRSF1A	7132	-0.40	5.63E-06	0.000307835
Medium: Day 1	ENSG00000069424	KCNAB2	8514	-0.65	5.65E-06	0.000307835
Medium: Day 1	ENSG00000134138	MEIS2	4212	-0.53	5.66E-06	0.000307835
Medium: Day 1	ENSG00000149948	HMGA2	8091	0.54	5.72E-06	0.000309953
Medium: Day 1	ENSG00000137193	PIM1	5292	-0.71	5.89E-06	0.000317805
Medium: Day 1	ENSG00000123405	NFE2	4778	-1.06	5.91E-06	0.000318019
Medium: Day 1	ENSG00000161682	FAM171A2	284069	-1.18	5.95E-06	0.000318476
Medium: Day 1	ENSG00000160211	G6PD	2539	-0.49	5.97E-06	0.000318476
Medium: Day 1	ENSG00000113739	STC2	8614	1.07	5.98E-06	0.000318476
Medium: Day 1	ENSG00000170485	NPAS2	4862	0.49	6.10E-06	0.000323702
Medium: Day 1	ENSG00000119698	PPP4R4	57718	0.67	6.16E-06	0.000325527
Medium: Day 1	ENSG00000126803	HSPA2	3306	-0.62	6.23E-06	0.000328313
Medium: Day 1	ENSG00000105664	COMP	1311	-1.25	6.42E-06	0.000337057
Medium: Day 1	ENSG00000196569	LAMA2	3908	-0.77	6.46E-06	0.000337995
Medium: Day 1	ENSG00000111907	TPD52L1	7164	-0.83	6.48E-06	0.000337995
Medium: Day 1	ENSG00000140526	ABHD2	11057	0.40	6.59E-06	0.000342522
Medium: Day 1	ENSG00000196549	MME	4311	0.79	6.68E-06	0.000346217
Medium: Day 1	ENSG00000115009	CCL20	6364	2.85	6.72E-06	0.00034654
Medium: Day 1	ENSG00000026297	RNASET2	8635	-0.53	6.73E-06	0.00034654
Medium: Day 1	ENSG00000137834	SMAD6	4091	-0.82	6.91E-06	0.000354564
Medium: Day 1	ENSG00000082497	SERTAD4	56256	-0.63	6.99E-06	0.000357596
Medium: Day 1	ENSG00000154319	FAM167A	83648	0.72	7.04E-06	0.00035865
Medium: Day 1	ENSG00000160408	ST6GALNAC6	30815	-0.52	7.06E-06	0.000358655
Medium: Day 1	ENSG00000167244	IGF2	3481	-0.85	7.08E-06	0.000358655
Medium: Day 1	ENSG00000122862	SRGN	5552	0.45	7.12E-06	0.000359306
Medium: Day 1	ENSG00000038295	TLL1	7092	-1.41	7.16E-06	0.000360232

Medium: Day 1	ENSG00000137070	IL11RA	3590	-0.51	7.37E-06	0.00036886
Medium: Day 1	ENSG00000126778	SIX1	6495	-0.77	7.38E-06	0.00036886
Medium: Day 1	ENSG00000127241	MASP1	5648	0.71	7.48E-06	0.000371741
Medium: Day 1	ENSG00000164619	BMPER	168667	1.03	7.49E-06	0.000371741
Medium: Day 1	ENSG00000135821	GLUL	2752	-0.50	7.51E-06	0.000371741
Medium: Day 1	ENSG00000180660	MAB21L1	4081	-0.42	7.53E-06	0.000371741
Medium: Day 1	ENSG00000005471	ABCB4	5244	-1.47	7.67E-06	0.000377099
Medium: Day 1	ENSG00000170962	PDGFD	80310	-1.13	7.71E-06	0.000377757
Medium: Day 1	ENSG00000100439	ABHD4	63874	-0.53	7.82E-06	0.000381193
Medium: Day 1	ENSG00000152127	MGAT5	4249	-0.36	7.84E-06	0.000381193
Medium: Day 1	ENSG00000187244	BCAM	4059	-0.53	7.85E-06	0.000381193
Medium: Day 1	ENSG00000109099	PMP22	5376	-0.38	7.89E-06	0.000382158
Medium: Day 1	ENSG00000164308	ERAP2	64167	0.38	8.13E-06	0.000389798
Medium: Day 1	ENSG00000151632	AKR1C2	1646	-0.97	8.14E-06	0.000389798
Medium: Day 1	ENSG00000164694	FNDC1	84624	1.25	8.15E-06	0.000389798
Medium: Day 1	ENSG00000136859	ANGPTL2	23452	-0.68	8.15E-06	0.000389798
Medium: Day 1	ENSG00000143341	HMCN1	83872	-1.07	8.29E-06	0.000395202
Medium: Day 1	ENSG00000028137	TNFRSF1B	7133	0.60	8.39E-06	0.000398145
Medium: Day 1	ENSG00000213626	LBH	81606	-1.24	8.40E-06	0.000398145
Medium: Day 1	ENSG00000145246	ATP10D	57205	-0.37	8.46E-06	0.000399436
Medium: Day 1	ENSG00000221869	CEBPD	1052	-0.62	8.56E-06	0.000402843
Medium: Day 1	ENSG00000115594	IL1R1	3554	-0.68	8.59E-06	0.000403078
Medium: Day 1	ENSG00000121281	ADCY7	113	-0.54	8.72E-06	0.000407478
Medium: Day 1	ENSG00000151090	THRB	7068	-0.95	8.73E-06	0.000407478
Medium: Day 1	ENSG00000106853	PTGR1	22949	-0.41	8.76E-06	0.000407696
Medium: Day 1	ENSG00000174939	ASPHD1	253982	0.60	8.79E-06	0.000407763
Medium: Day 1	ENSG00000166670	MMP10	4319	1.37	8.84E-06	0.000408608
Medium: Day 1	ENSG00000126458	RRAS	6237	0.56	8.94E-06	0.000412404
Medium: Day 1	ENSG00000184985	SORCS2	57537	1.03	9.05E-06	0.000415984
Medium: Day 1	ENSG00000150636	CCDC102B	79839	-0.88	9.13E-06	0.000418542
Medium: Day 1	ENSG00000122507	BBS9	27241	-0.54	9.23E-06	0.000421341
Medium: Day 1	ENSG00000008311	AASS	10157	-0.54	9.25E-06	0.000421341
Medium: Day 1	ENSG00000083290	ULK2	9706	-0.42	9.27E-06	0.000421341
Medium: Day 1	ENSG00000059915	PSD	5662	-0.67	9.53E-06	0.000431882
Medium: Day 1	ENSG00000143416	SELENBP1	8991	-0.48	9.63E-06	0.000435043
Medium: Day 3	ENSG00000019582	CD74	972	1.89	3.20E-12	4.88E-08
Medium: Day 3	ENSG00000163131	CTSS	1520	1.53	9.24E-11	7.05E-07
Medium: Day 3	ENSG00000221852	KRTAP1-5	83895	1.12	4.27E-09	1.83E-05
Medium: Day 3	ENSG00000234745	HLA-B	3106	1.03	4.81E-09	1.83E-05
Medium: Day 3	ENSG00000196878	LAMB3	3914	0.84	1.38E-08	4.17E-05
Medium: Day 3	ENSG00000085117	CD82	3732	0.99	1.64E-08	4.17E-05

Medium: Day 3	ENSG00000117594	HSD11B1	3290	2.15	2.07E-08	4.25E-05
Medium: Day 3	ENSG00000108797	CNTNAP1	8506	0.92	2.23E-08	4.25E-05
Medium: Day 3	ENSG00000105825	TFPI2	7980	1.80	2.91E-08	4.94E-05
Medium: Day 3	ENSG00000006118	TMEM132A	54972	1.45	4.00E-08	6.10E-05
Medium: Day 3	ENSG00000118503	TNFAIP3	7128	1.49	6.01E-08	8.01E-05
Medium: Day 3	ENSG00000050344	NFE2L3	9603	1.51	6.45E-08	8.01E-05
Medium: Day 3	ENSG00000134363	FST	10468	0.94	6.83E-08	8.01E-05
Medium: Day 3	ENSG00000105664	COMP	1311	-2.05	8.54E-08	8.87E-05
Medium: Day 3	ENSG00000167244	IGF2	3481	-1.50	8.91E-08	8.87E-05
Medium: Day 3	ENSG00000149571	KIRREL3	84623	1.10	9.30E-08	8.87E-05
Medium: Day 3	ENSG00000041982	TNC	3371	1.41	1.00E-07	8.97E-05
Medium: Day 3	ENSG00000187957	DNER	92737	1.88	1.13E-07	9.56E-05
Medium: Day 3	ENSG00000204642	HLA-F	3134	1.17	1.48E-07	0.000118894
Medium: Day 3	ENSG00000104321	TRPA1	8989	1.06	1.69E-07	0.000127609
Medium: Day 3	ENSG00000206503	HLA-A	3105	0.72	1.80E-07	0.000127609
Medium: Day 3	ENSG00000211448	DIO2	1734	-1.81	1.84E-07	0.000127609
Medium: Day 3	ENSG00000184985	SORCS2	57537	1.03	2.42E-07	0.000160213
Medium: Day 3	ENSG00000113645	WWC1	23286	1.30	2.66E-07	0.000169131
Medium: Day 3	ENSG00000149131	SERPING1	710	-0.65	2.95E-07	0.000180128
Medium: Day 3	ENSG00000140961	OSGIN1	29948	-0.66	3.21E-07	0.000188262
Medium: Day 3	ENSG00000135373	EHF	26298	4.09	3.69E-07	0.000201756
Medium: Day 3	ENSG00000100739	BDKRB1	623	1.26	3.78E-07	0.000201756
Medium: Day 3	ENSG00000008517	IL32	9235	2.50	3.84E-07	0.000201756
Medium: Day 3	ENSG00000163735	CXCL5	6374	2.52	4.16E-07	0.000211339
Medium: Day 3	ENSG00000139211	AMIGO2	347902	-1.26	4.63E-07	0.000213313
Medium: Day 3	ENSG00000150630	VEGFC	7424	0.69	4.65E-07	0.000213313
Medium: Day 3	ENSG00000171345	KRT19	3880	0.73	4.66E-07	0.000213313
Medium: Day 3	ENSG00000115828	QPCT	25797	0.72	4.76E-07	0.000213313
Medium: Day 3	ENSG00000189058	APOD	347	-1.47	5.01E-07	0.000218062
Medium: Day 3	ENSG00000174938	SEZ6L2	26470	0.69	5.61E-07	0.000237773
Medium: Day 3	ENSG00000006016	CRLF1	9244	-1.12	6.84E-07	0.000280064
Medium: Day 3	ENSG00000188157	AGRN	375790	0.66	7.10E-07	0.000280064
Medium: Day 3	ENSG00000146233	CYP39A1	51302	-1.00	7.16E-07	0.000280064
Medium: Day 3	ENSG00000204525	HLA-C	3107	0.56	7.36E-07	0.000280414
Medium: Day 3	ENSG00000182809	CRIP2	1397	0.46	7.65E-07	0.000284555
Medium: Day 3	ENSG00000145777	TSLP	85480	1.33	8.27E-07	0.000299277
Medium: Day 3	ENSG00000125730	C3	718	1.03	8.44E-07	0.000299277
Medium: Day 3	ENSG00000143819	EPHX1	2052	-0.66	9.18E-07	0.000317612
Medium: Day 3	ENSG00000211445	GPX3	2878	-1.84	9.39E-07	0.000317612
Medium: Day 3	ENSG00000029153	ARNTL2	56938	0.68	9.71E-07	0.000317612
Medium: Day 3	ENSG00000023445	BIRC3	330	1.90	9.79E-07	0.000317612
Medium: Day 3	ENSG00000118515	SGK1	6446	-0.63	1.08E-06	0.000338314

Medium: Day 3	ENSG00000138031	ADCY3	109	-0.58	1.09E-06	0.000338314
Medium: Day 3	ENSG00000175538	KCNE3	10008	-1.16	1.20E-06	0.000365853
Medium: Day 3	ENSG00000138449	SLC40A1	30061	-0.99	1.24E-06	0.000371213
Medium: Day 3	ENSG00000175899	A2M	2	-1.59	1.31E-06	0.000385121
Medium: Day 3	ENSG00000136048	DRAM1	55332	0.80	1.40E-06	0.000396597
Medium: Day 3	ENSG00000006062	MAP3K14	9020	-0.73	1.40E-06	0.000396597
Medium: Day 3	ENSG00000198759	EGFL6	25975	-1.49	1.45E-06	0.000403152
Medium: Day 3	ENSG00000115380	EFEMP1	2202	-0.85	1.51E-06	0.000411747
Medium: Day 3	ENSG00000169239	CA5B	11238	-0.65	1.60E-06	0.000427758
Medium: Day 3	ENSG00000136244	IL6	3569	1.35	1.87E-06	0.000492339
Medium: Day 3	ENSG00000157368	IL34	146433	1.58	1.97E-06	0.000499263
Medium: Day 3	ENSG00000197381	ADARB1	104	-0.81	2.04E-06	0.000499263
Medium: Day 3	ENSG00000144476	ACKR3	57007	-1.12	2.06E-06	0.000499263
Medium: Day 3	ENSG00000126368	NR1D1	9572	0.82	2.08E-06	0.000499263
Medium: Day 3	ENSG00000126803	HSPA2	3306	-0.70	2.08E-06	0.000499263
Medium: Day 3	ENSG00000197632	SERPINB2	5055	2.40	2.11E-06	0.000499263
Medium: Day 3	ENSG00000126458	RRAS	6237	0.56	2.13E-06	0.000499263
Medium: Day 3	ENSG00000186417	GLDN	342035	-0.87	2.19E-06	0.00050224
Medium: Day 3	ENSG00000060656	PTPRU	10076	-0.62	2.21E-06	0.00050224
Medium: Day 3	ENSG00000166592	RRAD	6236	1.01	2.27E-06	0.000509342
Medium: Day 3	ENSG00000173918	C1QTNF1	114897	0.83	2.69E-06	0.000592519
Medium: Day 3	ENSG00000170891	CYTL1	54360	-1.15	2.72E-06	0.000592519
Medium: Day 3	ENSG00000134070	IRAK2	3656	0.75	2.86E-06	0.000613521
Medium: Day 3	ENSG00000119714	GPR68	8111	1.07	2.90E-06	0.000613851
Medium: Day 3	ENSG00000120899	PTK2B	2185	-1.28	3.08E-06	0.000635886
Medium: Day 3	ENSG00000158714	SLAMF8	56833	2.92	3.09E-06	0.000635886
Medium: Day 3	ENSG00000116285	ERRFI1	54206	-0.50	3.18E-06	0.000646965
Medium: Day 3	ENSG00000104856	RELB	5971	0.86	3.71E-06	0.000744042
Medium: Day 3	ENSG00000062282	DGAT2	84649	1.14	3.82E-06	0.000755976
Medium: Day 3	ENSG00000164307	ERAP1	51752	0.52	3.92E-06	0.00076598
Medium: Day 3	ENSG00000197208	SLC22A4	6583	0.58	4.17E-06	0.00080094
Medium: Day 3	ENSG00000165804	ZNF219	51222	0.54	4.22E-06	0.00080094
Medium: Day 3	ENSG00000101938	CHRDL1	91851	-1.21	4.28E-06	0.00080094
Medium: Day 3	ENSG00000127951	FGL2	10875	-1.61	4.33E-06	0.00080094
Medium: Day 3	ENSG00000162804	SNED1	25992	-1.01	4.36E-06	0.00080094
Medium: Day 3	ENSG00000163739	CXCL1	2919	1.18	4.61E-06	0.000835074
Medium: Day 3	ENSG00000154122	ANKH	56172	-0.65	4.66E-06	0.000835074
Medium: Day 3	ENSG00000124875	CXCL6	6372	2.03	4.85E-06	0.000843375
Medium: Day 3	ENSG00000137193	PIM1	5292	-0.65	4.91E-06	0.000843375
Medium: Day 3	ENSG00000198074	AKR1B10	57016	-1.72	4.94E-06	0.000843375
Medium: Day 3	ENSG00000159674	SPON2	10417	-0.80	4.98E-06	0.000843375
Medium: Day 3	ENSG00000166710	B2M	567	0.59	5.02E-06	0.000843375

Medium: Day 3	ENSG00000198848	CES1	1066	-2.18	5.03E-06	0.000843375
Medium: Day 3	ENSG00000116690	PRG4	10216	-1.53	5.43E-06	0.000895868
Medium: Day 3	ENSG00000171509	RXFP1	59350	0.75	5.49E-06	0.000895868
Medium: Day 3	ENSG00000072210	ALDH3A2	224	-0.63	5.52E-06	0.000895868
Medium: Day 3	ENSG00000156265	MAP3K7CL	56911	0.91	5.59E-06	0.000896542
Medium: Day 3	ENSG00000185565	LSAMP	4045	-0.85	5.66E-06	0.000896542
Medium: Day 3	ENSG00000129988	LBP	3929	-1.71	5.70E-06	0.000896542
Medium: Day 3	ENSG00000065534	MYLK	4638	0.59	5.87E-06	0.000912558
Medium: Day 3	ENSG00000120708	TGFBI	7045	-0.61	5.99E-06	0.000923027
Medium: Day 3	ENSG00000105518	TMEM205	374882	0.54	6.15E-06	0.000937219
Medium: Day 3	ENSG00000121281	ADCY7	113	-0.62	6.23E-06	0.000940447
Medium: Day 3	ENSG00000161570	CCL5	6352	2.81	6.49E-06	0.000967528
Medium: Day 3	ENSG00000096060	FKBP5	2289	-0.52	6.54E-06	0.000967528
Medium: Day 3	ENSG00000165092	ALDH1A1	216	-2.30	6.80E-06	0.000996432
Medium: Day 3	ENSG00000243753	HLA-L	3139	0.70	7.28E-06	0.001057272
Medium: Day 3	ENSG00000146232	NFKBIE	4794	0.88	7.65E-06	0.001100623
Medium: Day 3	ENSG00000143382	ADAMTSL4	54507	-0.53	7.92E-06	0.001128019
Medium: Day 3	ENSG00000146122	DAAM2	23500	-0.82	8.44E-06	0.001191758
Medium: Day 3	ENSG00000104332	SFRP1	6422	-0.76	8.57E-06	0.001198563
Medium: Day 3	ENSG00000115963	RND3	390	0.43	8.93E-06	0.001235054
Medium: Day 3	ENSG00000090339	ICAM1	3383	1.31	8.99E-06	0.001235054
Medium: Day 3	ENSG00000104081	BMF	90427	-1.17	9.11E-06	0.001239313
Medium: Day 3	ENSG00000047457	CP	1356	-1.23	9.23E-06	0.001239313
Medium: Day 3	ENSG00000151632	AKR1C2	1646	-1.05	9.27E-06	0.001239313
Medium: Day 3	ENSG00000174080	CTSF	8722	-0.52	9.53E-06	0.001262969
Medium: Day 3	ENSG00000183454	GRIN2A	2903	2.27	9.79E-06	0.001278633
Medium: Day 3	ENSG00000152953	STK32B	55351	-0.49	9.81E-06	0.001278633
Medium: Day 3	ENSG00000170962	PDGFD	80310	-1.03	1.08E-05	0.001384004
Medium: Day 3	ENSG00000160326	SLC2A6	11182	0.82	1.08E-05	0.001384004
Medium: Day 3	ENSG00000074410	CA12	771	0.69	1.13E-05	0.001432053
Medium: Day 3	ENSG00000087116	ADAMTS2	9509	-0.52	1.14E-05	0.001436416
Medium: Day 3	ENSG00000112096	SOD2	6648	0.75	1.15E-05	0.001440314
Medium: Day 3	ENSG00000128342	LIF	3976	1.24	1.26E-05	0.001565754
Medium: Day 3	ENSG00000141150	RASL10B	91608	-0.74	1.28E-05	0.001571423
Medium: Day 3	ENSG00000120498	TEX11	56159	1.78	1.32E-05	0.00160474
Medium: Day 3	ENSG00000167191	GPRC5B	51704	-0.56	1.35E-05	0.001631627
Medium: Day 3	ENSG00000146411	SLC2A12	154091	-0.97	1.36E-05	0.001631627
Medium: Day 3	ENSG00000111670	GNPTAB	79158	0.53	1.42E-05	0.001675903
Medium: Day 3	ENSG00000135744	AGT	183	-1.75	1.42E-05	0.001675903
Medium: Day 3	ENSG00000092010	PSME1	5720	0.54	1.43E-05	0.001675903
Medium: Day 3	ENSG00000241399	CD302	9936	-0.91	1.46E-05	0.001701574
Medium: Day 3	ENSG00000159399	HK2	3099	-0.43	1.54E-05	0.001776465

Medium: Day 3	ENSG00000243649	CFB	629	1.21	1.58E-05	0.001808028
Medium: Day 3	ENSG00000129667	RHBDF2	79651	0.72	1.59E-05	0.001808028
Medium: Day 3	ENSG00000157766	ACAN	176	-3.16	1.63E-05	0.001836233
Medium: Day 3	ENSG00000163347	CLDN1	9076	2.95	1.65E-05	0.001853153
Medium: Day 3	ENSG00000129009	ISLR	3671	-0.86	1.69E-05	0.001878242
Medium: Day 3	ENSG00000122729	ACO1	48	0.47	1.71E-05	0.001892228
Medium: Day 3	ENSG00000185585	OLFML2A	169611	-0.60	1.76E-05	0.001927693
Medium: Day 3	ENSG00000110031	LPXN	9404	0.71	1.79E-05	0.001933736
Medium: Day 3	ENSG00000172197	MBOAT1	154141	-0.97	1.80E-05	0.001933736
Medium: Day 3	ENSG00000082497	SERTAD4	56256	-0.91	1.80E-05	0.001933736
Medium: Day 3	ENSG00000164619	BMPER	168667	0.81	1.82E-05	0.001942635
Medium: Day 3	ENSG00000110900	TSPAN11	441631	-1.08	1.84E-05	0.001946071
Medium: Day 3	ENSG00000144810	COL8A1	1295	0.66	1.88E-05	0.001973591
Medium: Day 3	ENSG00000081923	ATP8B1	5205	0.56	1.95E-05	0.002035813
Medium: Day 3	ENSG00000146457	WTAP	9589	0.51	1.97E-05	0.002046627
Medium: Day 3	ENSG00000010932	FMO1	2326	-1.77	1.99E-05	0.002050633
Medium: Day 3	ENSG00000174804	FZD4	8322	-1.01	2.06E-05	0.002100452
Medium: Day 3	ENSG00000105499	PLA2G4C	8605	0.79	2.07E-05	0.002100452
Medium: Day 3	ENSG00000197496	SLC2A10	81031	-0.58	2.12E-05	0.002144586
Medium: Day 3	ENSG00000206341	HLA-H	3136	0.63	2.14E-05	0.002144586
Medium: Day 3	ENSG00000145147	SLIT2	9353	0.52	2.20E-05	0.00218696
Medium: Day 3	ENSG00000172403	SYNPO2	171024	0.87	2.21E-05	0.00218696
Medium: Day 3	ENSG00000131323	TRAF3	7187	0.39	2.29E-05	0.002251913
Medium: Day 3	ENSG00000174348	PODN	127435	-0.81	2.35E-05	0.002300584
Medium: Day 3	ENSG00000145901	TNIP1	10318	0.35	2.42E-05	0.002321173
Medium: Day 3	ENSG00000231925	TAPBP	6892	0.51	2.43E-05	0.002321173
Medium: Day 3	ENSG00000105246	EBI3	10148	2.79	2.43E-05	0.002321173
Medium: Day 3	ENSG00000078725	BRINP1	1620	-1.07	2.44E-05	0.002321173
Medium: Day 3	ENSG00000103742	IGDCC4	57722	0.66	2.47E-05	0.002337901
Medium: Day 3	ENSG00000087086	FTL	2512	-0.53	2.49E-05	0.002337901
Medium: Day 3	ENSG00000184304	PRKD1	5587	0.58	2.51E-05	0.002337901
Medium: Day 3	ENSG00000168685	IL7R	3575	0.95	2.51E-05	0.002337901
Medium: Day 3	ENSG00000100364	KIAA0930	23313	-0.48	2.53E-05	0.002340913
Medium: Day 3	ENSG00000163520	FBLN2	2199	-0.67	2.65E-05	0.002426942
Medium: Day 3	ENSG00000148175	STOM	2040	-0.64	2.66E-05	0.002426942
Medium: Day 3	ENSG00000183287	CCBE1	147372	0.37	2.72E-05	0.00247041
Medium: Day 3	ENSG00000196917	HCAR1	27198	-0.93	2.80E-05	0.002521463
Medium: Day 3	ENSG00000240583	AQP1	358	-1.14	2.81E-05	0.002521463
Medium: Day 3	ENSG00000113739	STC2	8614	0.91	2.85E-05	0.002530446
Medium: Day 3	ENSG00000116260	QSOX1	5768	0.39	2.85E-05	0.002530446
Medium: Day 3	ENSG00000100911	PSME2	5721	0.67	2.90E-05	0.002555046
Medium: Day 3	ENSG00000163734	CXCL3	2921	1.14	2.95E-05	0.002580852

Medium: Day 3	ENSG00000136717	BIN1	274	-0.45	3.03E-05	0.002636262
Medium: Day 3	ENSG00000142657	PGD	5226	-0.36	3.07E-05	0.002655871
Medium: Day 3	ENSG00000178573	MAF	4094	-0.96	3.09E-05	0.002659272
Medium: Day 3	ENSG00000132000	PODNL1	79883	-0.74	3.10E-05	0.002659272
Medium: Day 3	ENSG00000128641	MYO1B	4430	0.45	3.20E-05	0.002727812
Medium: Day 3	ENSG00000164938	TP53INP1	94241	-0.51	3.27E-05	0.002755709
Medium: Day 3	ENSG00000174939	ASPHD1	253982	0.77	3.28E-05	0.002755709
Medium: Day 3	ENSG00000197971	MBP	4155	-0.64	3.29E-05	0.002755709
Medium: Day 3	ENSG00000135218	CD36	948	-1.04	3.40E-05	0.002831792
Medium: Day 3	ENSG00000048540	LMO3	55885	-1.48	3.42E-05	0.002831792
Medium: Day 3	ENSG00000154175	ABI3BP	25890	1.56	3.51E-05	0.002882821
Medium: Day 3	ENSG00000141404	GNAL	2774	-1.39	3.52E-05	0.002882821
Medium: Day 3	ENSG00000145390	USP53	54532	0.64	3.54E-05	0.002884133
Medium: Day 3	ENSG00000184374	COLEC10	10584	-0.75	3.63E-05	0.002947418
Medium: Day 3	ENSG00000204622	HLA-J	3137	1.08	3.67E-05	0.002958344
Treatment: Day 1	ENSG00000096060	FKBP5	2289	2.00	1.29E-13	2.00E-09
Treatment: Day 1	ENSG00000134363	FST	10468	-1.52	1.74E-12	1.25E-08
Treatment: Day 1	ENSG00000169715	MT1E	4493	1.33	2.42E-12	1.25E-08
Treatment: Day 1	ENSG00000179094	PER1	5187	1.27	6.60E-12	2.56E-08
Treatment: Day 1	ENSG00000046653	GPM6B	2824	1.44	3.63E-11	1.12E-07
Treatment: Day 1	ENSG00000204941	PSG5	5673	-0.88	2.12E-10	5.48E-07
Treatment: Day 1	ENSG00000168398	BDKRB2	624	-1.14	2.94E-10	6.50E-07
Treatment: Day 1	ENSG00000120129	DUSP1	1843	0.96	5.31E-10	1.03E-06
Treatment: Day 1	ENSG00000173432	SAA1	6288	2.14	1.11E-09	1.91E-06
Treatment: Day 1	ENSG00000143127	ITGA10	8515	2.53	1.46E-09	2.08E-06
Treatment: Day 1	ENSG00000125148	MT2A	4502	1.34	1.47E-09	2.08E-06
Treatment: Day 1	ENSG00000188487	INSC	387755	2.40	1.79E-09	2.10E-06
Treatment: Day 1	ENSG00000136997	MYC	4609	0.99	1.90E-09	2.10E-06
Treatment: Day 1	ENSG00000205364	MT1M	4499	1.48	2.17E-09	2.10E-06
Treatment: Day 1	ENSG00000101265	RASSF2	9770	-1.42	2.18E-09	2.10E-06
Treatment: Day 1	ENSG00000185432	METTL7A	25840	1.18	2.29E-09	2.10E-06
Treatment: Day 1	ENSG00000145777	TSLP	85480	-1.25	2.30E-09	2.10E-06
Treatment: Day 1	ENSG00000189221	MAOA	4128	1.40	3.20E-09	2.63E-06
Treatment: Day 1	ENSG00000164379	FOXQ1	94234	-1.46	3.23E-09	2.63E-06
Treatment: Day 1	ENSG00000135821	GLUL	2752	0.91	3.73E-09	2.89E-06
Treatment: Day 1	ENSG00000173376	NDNF	79625	-1.87	4.99E-09	3.68E-06
Treatment: Day 1	ENSG00000138385	SSB	6741	0.73	5.48E-09	3.71E-06
Treatment: Day 1	ENSG00000176692	FOXC2	2303	1.05	5.52E-09	3.71E-06
Treatment: Day 1	ENSG00000130513	GDF15	9518	-1.11	5.75E-09	3.71E-06
Treatment: Day 1	ENSG00000253368	TRNP1	388610	0.93	6.22E-09	3.86E-06
Treatment: Day 1	ENSG00000120708	TGFBI	7045	-0.88	7.07E-09	4.21E-06

Treatment: Day 1	ENSG00000260549	MT1L	4500	1.61	7.80E-09	4.47E-06
Treatment: Day 1	ENSG00000013293	SLC7A14	57709	-2.03	8.17E-09	4.52E-06
Treatment: Day 1	ENSG00000124766	SOX4	6659	-0.98	8.93E-09	4.77E-06
Treatment: Day 1	ENSG00000176597	B3GNT5	84002	1.34	9.57E-09	4.94E-06
Treatment: Day 1	ENSG00000114098	ARMC8	25852	0.59	1.03E-08	5.14E-06
Treatment: Day 1	ENSG00000171509	RXFP1	59350	-1.41	1.14E-08	5.50E-06
Treatment: Day 1	ENSG00000154864	PIEZO2	63895	-1.56	1.17E-08	5.50E-06
Treatment: Day 1	ENSG00000187193	MT1X	4501	1.86	1.29E-08	5.72E-06
Treatment: Day 1	ENSG00000123610	TNFAIP6	7130	-0.92	1.29E-08	5.72E-06
Treatment: Day 1	ENSG00000095303	PTGS1	5742	0.78	1.35E-08	5.74E-06
Treatment: Day 1	ENSG00000133110	POSTN	10631	-1.58	1.37E-08	5.74E-06
Treatment: Day 1	ENSG00000100739	BDKRB1	623	-1.31	1.70E-08	6.83E-06
Treatment: Day 1	ENSG00000136859	ANGPTL2	23452	-1.12	1.73E-08	6.83E-06
Treatment: Day 1	ENSG00000152463	OLAH	55301	2.41	1.76E-08	6.83E-06
Treatment: Day 1	ENSG00000145244	CORIN	10699	3.07	2.44E-08	9.24E-06
Treatment: Day 1	ENSG00000232956	SNHG15	285958	0.80	3.47E-08	1.26E-05
Treatment: Day 1	ENSG00000163820	FYCO1	79443	-0.54	3.51E-08	1.26E-05
Treatment: Day 1	ENSG00000131370	SH3BP5	9467	-0.62	3.79E-08	1.33E-05
Treatment: Day 1	ENSG00000155760	FZD7	8324	-0.80	4.00E-08	1.35E-05
Treatment: Day 1	ENSG00000180875	GREM2	64388	-0.84	4.02E-08	1.35E-05
Treatment: Day 1	ENSG00000231924	PSG1	5669	-1.11	4.15E-08	1.36E-05
Treatment: Day 1	ENSG00000164929	BAALC	79870	-1.60	4.22E-08	1.36E-05
Treatment: Day 1	ENSG00000115461	IGFBP5	3488	-1.40	4.94E-08	1.56E-05
Treatment: Day 1	ENSG00000109906	ZBTB16	7704	4.83	5.38E-08	1.67E-05
Treatment: Day 1	ENSG00000138166	DUSP5	1847	0.65	5.60E-08	1.67E-05
Treatment: Day 1	ENSG00000135424	ITGA7	3679	-0.79	5.60E-08	1.67E-05
Treatment: Day 1	ENSG00000087494	PTHLH	5744	-2.07	5.94E-08	1.74E-05
Treatment: Day 1	ENSG00000065328	MCM10	55388	0.91	6.48E-08	1.86E-05
Treatment: Day 1	ENSG00000108960	MMD	23531	0.70	6.66E-08	1.88E-05
Treatment: Day 1	ENSG00000099860	GADD45B	4616	0.86	6.96E-08	1.93E-05
Treatment: Day 1	ENSG00000143320	CRABP2	1382	-1.01	7.50E-08	2.04E-05
Treatment: Day 1	ENSG00000135362	PRR5L	79899	1.38	7.86E-08	2.10E-05
Treatment: Day 1	ENSG00000168679	SLC16A4	9122	-0.97	8.04E-08	2.11E-05
Treatment: Day 1	ENSG00000171867	PRNP	5621	-0.65	8.63E-08	2.23E-05
Treatment: Day 1	ENSG00000171451	DSEL	92126	-0.57	9.00E-08	2.28E-05
Treatment: Day 1	ENSG00000116991	SIPA1L2	57568	-0.96	9.20E-08	2.30E-05
Treatment: Day 1	ENSG00000158258	CLSTN2	64084	-1.30	9.79E-08	2.41E-05
Treatment: Day 1	ENSG00000157150	TIMP4	7079	0.99	1.12E-07	2.72E-05
Treatment: Day 1	ENSG00000166963	MAP1A	4130	-0.83	1.17E-07	2.78E-05
Treatment: Day 1	ENSG00000079257	LXN	56925	-0.80	1.19E-07	2.80E-05
Treatment: Day 1	ENSG00000172986	GXYLT2	727936	-1.03	1.21E-07	2.80E-05
Treatment: Day 1	ENSG00000204634	TBC1D8	11138	0.84	1.49E-07	3.37E-05

Treatment: Day 1	ENSG00000147852	VLDLR	7436	-1.60	1.50E-07	3.37E-05
Treatment: Day 1	ENSG00000132603	NIP7	51388	0.54	1.54E-07	3.41E-05
Treatment: Day 1	ENSG00000182197	EXT1	2131	-0.54	1.63E-07	3.53E-05
Treatment: Day 1	ENSG00000187955	COL14A1	7373	-1.10	1.64E-07	3.53E-05
Treatment: Day 1	ENSG00000171621	SPSB1	80176	-0.54	1.70E-07	3.60E-05
Treatment: Day 1	ENSG00000041982	TNC	3371	-0.96	1.91E-07	4.00E-05
Treatment: Day 1	ENSG00000162616	DNAJB4	11080	0.66	2.02E-07	4.18E-05
Treatment: Day 1	ENSG00000146250	PRSS35	167681	-1.67	2.16E-07	4.38E-05
Treatment: Day 1	ENSG00000120658	ENOX1	55068	-0.75	2.20E-07	4.38E-05
Treatment: Day 1	ENSG00000172061	LRRC15	131578	-1.39	2.20E-07	4.38E-05
Treatment: Day 1	ENSG00000187210	GCNT1	2650	0.60	2.37E-07	4.65E-05
Treatment: Day 1	ENSG00000113361	CDH6	1004	-0.70	2.41E-07	4.67E-05
Treatment: Day 1	ENSG00000226950	DANCR	57291	0.84	2.70E-07	5.17E-05
Treatment: Day 1	ENSG00000103381	CPPED1	55313	0.52	2.87E-07	5.41E-05
Treatment: Day 1	ENSG00000141150	RASL10B	91608	1.11	2.92E-07	5.45E-05
Treatment: Day 1	ENSG00000148848	ADAM12	8038	-0.69	3.01E-07	5.56E-05
Treatment: Day 1	ENSG00000255717	SNHG1	23642	0.63	3.06E-07	5.57E-05
Treatment: Day 1	ENSG00000140285	FGF7	2252	-0.72	3.15E-07	5.67E-05
Treatment: Day 1	ENSG00000178921	PFAS	5198	0.65	3.32E-07	5.84E-05
Treatment: Day 1	ENSG00000166197	NOLC1	9221	0.57	3.32E-07	5.84E-05
Treatment: Day 1	ENSG00000155511	GRIA1	2890	1.06	3.39E-07	5.87E-05
Treatment: Day 1	ENSG00000125845	BMP2	650	-1.05	3.45E-07	5.87E-05
Treatment: Day 1	ENSG00000144381	HSPD1	3329	0.63	3.47E-07	5.87E-05
Treatment: Day 1	ENSG00000073756	PTGS2	5743	-1.99	3.52E-07	5.87E-05
Treatment: Day 1	ENSG00000227051	C14orf132	56967	-0.66	3.52E-07	5.87E-05
Treatment: Day 1	ENSG00000185565	LSAMP	4045	-0.69	3.65E-07	6.02E-05
Treatment: Day 1	ENSG00000154654	NCAM2	4685	-0.73	3.74E-07	6.10E-05
Treatment: Day 1	ENSG00000168079	SCARA5	286133	-1.15	3.87E-07	6.25E-05
Treatment: Day 1	ENSG00000132561	MATN2	4147	-0.82	4.06E-07	6.48E-05
Treatment: Day 1	ENSG00000139329	LUM	4060	-0.85	4.11E-07	6.49E-05
Treatment: Day 1	ENSG00000109072	VTN	7448	-0.83	4.33E-07	6.77E-05
Treatment: Day 1	ENSG00000013297	CLDN11	5010	-0.65	4.46E-07	6.91E-05
Treatment: Day 1	ENSG00000198910	L1CAM	3897	-1.24	4.79E-07	7.34E-05
Treatment: Day 1	ENSG00000168140	VASN	114990	-0.66	4.88E-07	7.41E-05
Treatment: Day 1	ENSG00000198612	COPS8	10920	0.49	5.12E-07	7.70E-05
Treatment: Day 1	ENSG00000145911	N4BP3	23138	-1.55	5.26E-07	7.83E-05
Treatment: Day 1	ENSG00000114251	WNT5A	7474	-0.74	5.57E-07	8.22E-05
Treatment: Day 1	ENSG00000134321	RSAD2	91543	-2.30	5.71E-07	8.35E-05
Treatment: Day 1	ENSG00000109084	TMEM97	27346	0.68	6.25E-07	9.04E-05
Treatment: Day 1	ENSG00000137809	ITGA11	22801	-1.28	6.55E-07	9.39E-05
Treatment: Day 1	ENSG00000198805	PNP	4860	0.57	6.63E-07	9.40E-05
Treatment: Day 1	ENSG00000170214	ADRA1B	147	1.34	6.71E-07	9.40E-05

Treatment: Day 1	ENSG00000143816	WNT9A	7483	-0.86	6.76E-07	9.40E-05
Treatment: Day 1	ENSG00000171345	KRT19	3880	-1.22	6.84E-07	9.40E-05
Treatment: Day 1	ENSG00000158716	DUSP23	54935	0.74	6.86E-07	9.40E-05
Treatment: Day 1	ENSG00000169744	LDB2	9079	-0.85	6.96E-07	9.45E-05
Treatment: Day 1	ENSG00000163735	CXCL5	6374	1.54	7.42E-07	1.00E-04
Treatment: Day 1	ENSG00000143476	DTL	51514	0.76	7.48E-07	1.00E-04
Treatment: Day 1	ENSG00000048162	NOP16	51491	0.68	7.74E-07	0.000102434
Treatment: Day 1	ENSG00000117877	CD3EAP	10849	0.76	7.86E-07	0.000103164
Treatment: Day 1	ENSG00000115523	GNLY	10578	1.54	8.00E-07	0.000104175
Treatment: Day 1	ENSG00000102057	KCND1	3750	-0.75	8.33E-07	0.000107525
Treatment: Day 1	ENSG00000164045	CDC25A	993	0.77	8.59E-07	0.000109984
Treatment: Day 1	ENSG00000179314	WSCD1	23302	0.96	8.66E-07	0.000109984
Treatment: Day 1	ENSG00000003989	SLC7A2	6542	1.69	8.77E-07	0.000110462
Treatment: Day 1	ENSG00000164920	OSR2	116039	-0.84	9.06E-07	0.000112511
Treatment: Day 1	ENSG00000145220	LYAR	55646	0.73	9.11E-07	0.000112511
Treatment: Day 1	ENSG00000129467	ADCY4	196883	-1.00	9.22E-07	0.000112511
Treatment: Day 1	ENSG00000092969	TGFB2	7042	-1.25	9.22E-07	0.000112511
Treatment: Day 1	ENSG00000100626	GALNT16	57452	-0.87	9.64E-07	0.000116666
Treatment: Day 1	ENSG00000157445	CACNA2D3	55799	-0.88	1.00E-06	0.000120287
Treatment: Day 1	ENSG00000067177	PHKA1	5255	0.84	1.02E-06	0.000121591
Treatment: Day 1	ENSG00000152402	GUCY1A2	2977	-1.93	1.03E-06	0.000121591
Treatment: Day 1	ENSG00000165272	AQP3	360	-1.00	1.04E-06	0.000121591
Treatment: Day 1	ENSG00000157514	TSC22D3	1831	1.11	1.12E-06	0.000128866
Treatment: Day 1	ENSG00000159167	STC1	6781	0.94	1.12E-06	0.000128866
Treatment: Day 1	ENSG00000133059	DSTYK	25778	-0.41	1.12E-06	0.000128866
Treatment: Day 1	ENSG00000152377	SPOCK1	6695	-0.74	1.14E-06	0.000129666
Treatment: Day 1	ENSG00000105855	ITGB8	3696	-1.43	1.16E-06	0.000130732
Treatment: Day 1	ENSG00000174938	SEZ6L2	26470	-0.71	1.18E-06	0.000131555
Treatment: Day 1	ENSG00000093009	CDC45	8318	0.82	1.18E-06	0.000131555
Treatment: Day 1	ENSG00000106976	DNM1	1759	-0.78	1.20E-06	0.000132526
Treatment: Day 1	ENSG00000065534	MYLK	4638	-0.56	1.23E-06	0.000134943
Treatment: Day 1	ENSG00000178031	ADAMTSL1	92949	-0.65	1.25E-06	0.000135085
Treatment: Day 1	ENSG00000177192	PUS1	80324	0.61	1.26E-06	0.000135085
Treatment: Day 1	ENSG00000159674	SPON2	10417	-0.87	1.26E-06	0.000135085
Treatment: Day 1	ENSG00000184922	FMNL1	752	-0.57	1.28E-06	0.000136572
Treatment: Day 1	ENSG00000137266	SLC22A23	63027	-0.91	1.29E-06	0.000136572
Treatment: Day 1	ENSG00000049246	PER3	8863	-0.81	1.30E-06	0.000136572
Treatment: Day 1	ENSG00000165732	DDX21	9188	0.57	1.30E-06	0.000136572
Treatment: Day 1	ENSG00000165244	ZNF367	195828	0.59	1.33E-06	0.000137361
Treatment: Day 1	ENSG00000101003	GINS1	9837	0.60	1.34E-06	0.000137361
Treatment: Day 1	ENSG00000156011	PSD3	23362	-0.72	1.34E-06	0.000137361
Treatment: Day 1	ENSG00000196611	MMP1	4312	-0.92	1.35E-06	0.000137457

Treatment: Day 1	ENSG00000197989	SNHG12	85028	0.55	1.36E-06	0.000137457
Treatment: Day 1	ENSG00000139132	FGD4	121512	0.65	1.38E-06	0.000138457
Treatment: Day 1	ENSG00000185567	AHNAK2	113146	-0.62	1.44E-06	0.000143875
Treatment: Day 1	ENSG00000106617	PRKAG2	51422	0.49	1.47E-06	0.000146147
Treatment: Day 1	ENSG00000101361	NOP56	10528	0.58	1.51E-06	0.000148808
Treatment: Day 1	ENSG00000164053	ATRIP	84126	0.59	1.53E-06	0.000150288
Treatment: Day 1	ENSG00000184985	SORCS2	57537	-1.20	1.55E-06	0.000151392
Treatment: Day 1	ENSG00000196932	TMEM26	219623	-1.14	1.58E-06	0.000152478
Treatment: Day 1	ENSG00000171793	CTPS1	1503	0.59	1.58E-06	0.000152478
Treatment: Day 1	ENSG00000137501	SYTL2	54843	-0.81	1.60E-06	0.000153356
Treatment: Day 1	ENSG00000128815	WDFY4	57705	2.02	1.63E-06	0.00015514
Treatment: Day 1	ENSG00000054598	FOXC1	2296	-0.63	1.64E-06	0.000155339
Treatment: Day 1	ENSG00000128342	LIF	3976	-1.43	1.73E-06	0.000161793
Treatment: Day 1	ENSG00000162745	OLFML2B	25903	-1.15	1.74E-06	0.000161793
Treatment: Day 1	ENSG00000169764	UGP2	7360	0.44	1.74E-06	0.000161793
Treatment: Day 1	ENSG00000112715	VEGFA	7422	-0.70	1.76E-06	0.000162062
Treatment: Day 1	ENSG00000164099	PRSS12	8492	-0.50	1.79E-06	0.000163844
Treatment: Day 1	ENSG00000137094	DNAJB5	25822	-0.49	1.82E-06	0.000165671
Treatment: Day 1	ENSG00000134198	TSPAN2	10100	-1.30	1.83E-06	0.000165961
Treatment: Day 1	ENSG00000138131	LOXL4	84171	-0.61	1.86E-06	0.000166121
Treatment: Day 1	ENSG00000166923	GREM1	26585	-0.63	1.86E-06	0.000166121
Treatment: Day 1	ENSG00000145632	PLK2	10769	-0.63	1.87E-06	0.000166121
Treatment: Day 1	ENSG00000181938	GINS3	64785	0.63	1.97E-06	0.000173838
Treatment: Day 1	ENSG00000123374	CDK2	1017	0.51	2.00E-06	0.000173838
Treatment: Day 1	ENSG00000092853	CLSPN	63967	0.72	2.00E-06	0.000173838
Treatment: Day 1	ENSG00000171552	BCL2L1	598	0.50	2.01E-06	0.000173838
Treatment: Day 1	ENSG00000167088	SNRPD1	6632	0.48	2.02E-06	0.000173838
Treatment: Day 1	ENSG00000051180	RAD51	5888	0.57	2.03E-06	0.000173838
Treatment: Day 1	ENSG00000115844	DLX2	1746	-0.71	2.03E-06	0.000173838
Treatment: Day 1	ENSG00000172915	NBEA	26960	-0.60	2.06E-06	0.000174886
Treatment: Day 1	ENSG00000151503	NCAPD3	23310	0.49	2.10E-06	0.000174886
Treatment: Day 1	ENSG00000005238	FAM214B	80256	-0.41	2.10E-06	0.000174886
Treatment: Day 1	ENSG00000171848	RRM2	6241	0.55	2.10E-06	0.000174886
Treatment: Day 1	ENSG00000134013	LOXL2	4017	-0.49	2.11E-06	0.000174886
Treatment: Day 1	ENSG00000183160	TMEM119	338773	-0.77	2.13E-06	0.000174886
Treatment: Day 1	ENSG00000188157	AGRN	375790	-0.62	2.13E-06	0.000174886
Treatment: Day 1	ENSG00000189057	FAM111B	374393	0.72	2.13E-06	0.000174886
Treatment: Day 1	ENSG00000134830	C5AR2	27202	3.36	2.16E-06	0.000176007
Treatment: Day 1	ENSG00000116260	QSOX1	5768	-0.60	2.18E-06	0.000177167
Treatment: Day 1	ENSG00000115828	QPCT	25797	-0.60	2.24E-06	0.000180594
Treatment: Day 1	ENSG00000170515	PA2G4	5036	0.54	2.26E-06	0.0001811
Treatment: Day 1	ENSG00000134259	NGF	4803	-0.85	2.29E-06	0.000182435

Treatment: Day 1	ENSG00000150281	CTF1	1489	-0.59	2.31E-06	0.000182435
Treatment: Day 1	ENSG00000166592	RRAD	6236	-1.31	2.31E-06	0.000182435
Treatment: Day 1	ENSG00000178773	CPNE7	27132	0.44	2.33E-06	0.000182435
Treatment: Day 1	ENSG00000166448	TMEM130	222865	-0.82	2.33E-06	0.000182435
Treatment: Day 1	ENSG00000170791	CHCHD7	79145	0.47	2.35E-06	0.000182761
Treatment: Day 1	ENSG00000175305	CCNE2	9134	0.72	2.41E-06	0.000183842
Treatment: Day 1	ENSG00000104611	SH2D4A	63898	-0.64	2.41E-06	0.000183842
Treatment: Day 1	ENSG00000144749	LRIG1	26018	-0.60	2.42E-06	0.000183842
Treatment: Day 1	ENSG00000170891	CYTL1	54360	-0.68	2.43E-06	0.000183842
Treatment: Day 1	ENSG00000119630	PGF	5228	-0.93	2.43E-06	0.000183842
Treatment: Day 1	ENSG00000138669	PRKG2	5593	-1.03	2.43E-06	0.000183842
Treatment: Day 1	ENSG00000111641	NOP2	4839	0.53	2.48E-06	0.000186494
Treatment: Day 1	ENSG00000174371	EXO1	9156	0.78	2.52E-06	0.000187848
Treatment: Day 1	ENSG00000094804	CDC6	990	0.71	2.54E-06	0.000187848
Treatment: Day 1	ENSG00000158467	AHCYL2	23382	-0.55	2.54E-06	0.000187848
Treatment: Day 1	ENSG00000110104	CCDC86	79080	0.58	2.55E-06	0.000187848
Treatment: Day 1	ENSG00000155189	AGPAT5	55326	0.57	2.56E-06	0.000188229
Treatment: Day 1	ENSG00000144218	AFF3	3899	-0.99	2.60E-06	0.000190139
Treatment: Day 1	ENSG00000242125	SNHG3	8420	0.67	2.67E-06	0.000193987
Treatment: Day 1	ENSG00000070081	NUCB2	4925	-0.45	2.70E-06	0.000195366
Treatment: Day 1	ENSG00000171604	CXXC5	51523	-0.52	2.75E-06	0.00019797
Treatment: Day 1	ENSG00000161921	CXCL16	58191	-0.71	2.76E-06	0.00019797
Treatment: Day 1	ENSG00000204131	NHSL2	340527	-0.92	2.84E-06	0.000201256
Treatment: Day 1	ENSG00000116711	PLA2G4A	5321	-0.83	2.84E-06	0.000201256
Treatment: Day 1	ENSG00000197635	DPP4	1803	-0.58	2.85E-06	0.000201256
Treatment: Day 1	ENSG00000092470	WDR76	79968	0.68	2.91E-06	0.000204512
Treatment: Day 1	ENSG00000241878	PISD	23761	0.45	2.92E-06	0.000204512
Treatment: Day 1	ENSG00000160818	GPATCH4	54865	0.53	2.97E-06	0.000207368
Treatment: Day 1	ENSG00000178764	ZHX2	22882	-0.64	3.07E-06	0.000211637
Treatment: Day 1	ENSG00000135919	SERPINE2	5270	-0.82	3.08E-06	0.000211637
Treatment: Day 1	ENSG00000138363	ATIC	471	0.39	3.09E-06	0.000211637
Treatment: Day 1	ENSG00000134245	WNT2B	7482	-0.68	3.10E-06	0.000211637
Treatment: Day 1	ENSG00000112118	MCM3	4172	0.68	3.10E-06	0.000211637
Treatment: Day 1	ENSG00000144369	FAM171B	165215	-0.75	3.14E-06	0.000212921
Treatment: Day 1	ENSG00000115946	PNO1	56902	0.50	3.15E-06	0.000212921
Treatment: Day 1	ENSG00000171703	TCEA2	6919	-0.47	3.20E-06	0.000214898
Treatment: Day 1	ENSG00000090376	IRAK3	11213	0.54	3.20E-06	0.000214898
Treatment: Day 1	ENSG00000236104	ZBTB22	9278	-0.50	3.24E-06	0.000216572
Treatment: Day 1	ENSG00000124104	SNX21	90203	-0.49	3.30E-06	0.000219307
Treatment: Day 1	ENSG00000168496	FEN1	2237	0.65	3.34E-06	0.000221252
Treatment: Day 1	ENSG00000225968	ELFN1	392617	-0.86	3.45E-06	0.000227103
Treatment: Day 1	ENSG00000118655	DCLRE1B	64858	0.50	3.46E-06	0.000227103

Treatment: Day 1	ENSG00000100714	MTHFD1	4522	0.49	3.63E-06	0.000237237
Treatment: Day 1	ENSG00000176974	SHMT1	6470	0.63	3.64E-06	0.000237237
Treatment: Day 1	ENSG00000110436	SLC1A2	6506	-0.97	3.68E-06	0.000238808
Treatment: Day 1	ENSG00000174697	LEP	3952	1.78	3.72E-06	0.000239708
Treatment: Day 1	ENSG00000163737	PF4	5196	2.15	3.73E-06	0.000239708
Treatment: Day 1	ENSG00000221826	PSG3	5671	-2.02	3.82E-06	0.000244439
Treatment: Day 1	ENSG00000166341	DCHS1	8642	-0.72	3.85E-06	0.000245453
Treatment: Day 1	ENSG00000138074	SLC5A6	8884	0.66	3.91E-06	0.00024853
Treatment: Day 1	ENSG00000085840	ORC1	4998	0.87	3.96E-06	0.000248822
Treatment: Day 1	ENSG00000132436	FIGNL1	63979	0.52	4.00E-06	0.000248822
Treatment: Day 1	ENSG00000184220	CMSS1	84319	0.61	4.00E-06	0.000248822
Treatment: Day 1	ENSG00000129493	HEATR5A	25938	-0.54	4.01E-06	0.000248822
Treatment: Day 1	ENSG00000164619	BMPER	168667	-1.08	4.03E-06	0.000248822
Treatment: Day 1	ENSG00000123405	NFE2	4778	-1.10	4.03E-06	0.000248822
Treatment: Day 1	ENSG00000105825	TFPI2	7980	-0.51	4.05E-06	0.000248822
Treatment: Day 1	ENSG00000142178	SIK1	150094	1.11	4.06E-06	0.000248822
Treatment: Day 1	ENSG00000087245	MMP2	4313	-0.62	4.06E-06	0.000248822
Treatment: Day 1	ENSG00000163703	CRELD1	78987	-0.62	4.08E-06	0.000249061
Treatment: Day 1	ENSG00000070404	FSTL3	10272	0.48	4.16E-06	0.000252001
Treatment: Day 1	ENSG00000240184	PCDHGC3	5098	-0.51	4.18E-06	0.000252001
Treatment: Day 1	ENSG00000165480	SKA3	221150	0.60	4.18E-06	0.000252001
Treatment: Day 1	ENSG00000113368	LMNB1	4001	0.52	4.22E-06	0.000253285
Treatment: Day 1	ENSG00000173638	SLC19A1	6573	0.85	4.27E-06	0.000255583
Treatment: Day 1	ENSG00000167100	SAMD14	201191	-0.64	4.30E-06	0.000255637
Treatment: Day 1	ENSG00000160325	CACFD1	11094	-0.85	4.31E-06	0.000255637
Treatment: Day 1	ENSG00000141469	SLC14A1	6563	-2.12	4.36E-06	0.000257644
Treatment: Day 1	ENSG00000121057	AKAP1	8165	0.42	4.40E-06	0.000257644
Treatment: Day 1	ENSG00000104738	MCM4	4173	0.65	4.41E-06	0.000257644
Treatment: Day 1	ENSG00000196517	SLC6A9	6536	-1.51	4.44E-06	0.000257644
Treatment: Day 1	ENSG00000136160	EDNRB	1910	-0.81	4.45E-06	0.000257644
Treatment: Day 1	ENSG00000233452	STXBP5-AS1	729178	-0.85	4.46E-06	0.000257644
Treatment: Day 1	ENSG00000178573	MAF	4094	-1.28	4.46E-06	0.000257644
Treatment: Day 1	ENSG00000082516	GEMIN5	25929	0.47	4.48E-06	0.000257644
Treatment: Day 1	ENSG00000157613	CREB3L1	90993	-0.45	4.49E-06	0.000257644
Treatment: Day 1	ENSG00000166173	LARP6	55323	-0.40	4.52E-06	0.000258525
Treatment: Day 1	ENSG00000101057	MYBL2	4605	0.79	4.57E-06	0.000260271
Treatment: Day 1	ENSG00000105894	PTN	5764	-0.42	4.63E-06	0.000262515
Treatment: Day 3	ENSG00000096060	FKBP5	2289	2.52	3.32E-14	5.05E-10
Treatment: Day 3	ENSG00000134363	FST	10468	-1.82	2.04E-11	1.55E-07
Treatment: Day 3	ENSG00000116991	SIPA1L2	57568	-1.39	4.48E-11	2.28E-07
Treatment: Day 3	ENSG00000171345	KRT19	3880	-1.49	7.74E-11	2.95E-07

Treatment: Day 3	ENSG00000176597	B3GNT5	84002	1.38	1.21E-10	3.38E-07
Treatment: Day 3	ENSG00000204941	PSG5	5673	-1.26	1.33E-10	3.38E-07
Treatment: Day 3	ENSG00000221852	KRTAP1-5	83895	-1.46	1.67E-10	3.64E-07
Treatment: Day 3	ENSG00000113361	CDH6	1004	-1.40	2.43E-10	4.63E-07
Treatment: Day 3	ENSG00000173432	SAA1	6288	3.17	3.22E-10	5.05E-07
Treatment: Day 3	ENSG00000143127	ITGA10	8515	2.59	3.31E-10	5.05E-07
Treatment: Day 3	ENSG00000157150	TIMP4	7079	1.65	3.79E-10	5.26E-07
Treatment: Day 3	ENSG00000171509	RXFP1	59350	-1.66	4.59E-10	5.83E-07
Treatment: Day 3	ENSG00000188487	INSC	387755	2.42	9.34E-10	1.10E-06
Treatment: Day 3	ENSG00000185432	METTL7A	25840	1.50	1.36E-09	1.48E-06
Treatment: Day 3	ENSG00000101265	RASSF2	9770	-2.44	1.72E-09	1.74E-06
Treatment: Day 3	ENSG00000169715	MT1E	4493	1.42	2.75E-09	2.62E-06
Treatment: Day 3	ENSG00000253368	TRNP1	388610	1.05	3.29E-09	2.95E-06
Treatment: Day 3	ENSG00000189221	MAOA	4128	2.15	4.51E-09	3.67E-06
Treatment: Day 3	ENSG00000179094	PER1	5187	1.02	4.91E-09	3.67E-06
Treatment: Day 3	ENSG00000162745	OLFML2B	25903	-1.45	5.02E-09	3.67E-06
Treatment: Day 3	ENSG00000162591	MEGF6	1953	-0.72	5.05E-09	3.67E-06
Treatment: Day 3	ENSG00000109906	ZBTB16	7704	3.99	5.48E-09	3.80E-06
Treatment: Day 3	ENSG00000172061	LRRC15	131578	-2.16	6.18E-09	4.08E-06
Treatment: Day 3	ENSG00000124766	SOX4	6659	-1.06	6.42E-09	4.08E-06
Treatment: Day 3	ENSG00000174938	SEZ6L2	26470	-0.99	7.96E-09	4.86E-06
Treatment: Day 3	ENSG00000046653	GPM6B	2824	2.00	1.25E-08	7.31E-06
Treatment: Day 3	ENSG00000135362	PRR5L	79899	1.49	1.43E-08	7.48E-06
Treatment: Day 3	ENSG00000135821	GLUL	2752	1.06	1.50E-08	7.48E-06
Treatment: Day 3	ENSG00000124191	TOX2	84969	0.83	1.50E-08	7.48E-06
Treatment: Day 3	ENSG00000049246	PER3	8863	-1.76	1.52E-08	7.48E-06
Treatment: Day 3	ENSG00000104611	SH2D4A	63898	-0.76	1.52E-08	7.48E-06
Treatment: Day 3	ENSG00000133110	POSTN	10631	-2.47	1.85E-08	8.79E-06
Treatment: Day 3	ENSG00000134259	NGF	4803	-1.25	1.96E-08	9.07E-06
Treatment: Day 3	ENSG00000187955	COL14A1	7373	-1.49	2.72E-08	1.22E-05
Treatment: Day 3	ENSG00000175040	CHST2	9435	0.86	2.98E-08	1.26E-05
Treatment: Day 3	ENSG00000184985	SORCS2	57537	-1.23	2.99E-08	1.26E-05
Treatment: Day 3	ENSG00000152463	OLAH	55301	2.71	3.08E-08	1.26E-05
Treatment: Day 3	ENSG00000174348	PODN	127435	-1.47	3.13E-08	1.26E-05
Treatment: Day 3	ENSG00000141469	SLC14A1	6563	-2.30	3.98E-08	1.56E-05
Treatment: Day 3	ENSG00000166311	SMPD1	6609	-0.60	4.27E-08	1.61E-05
Treatment: Day 3	ENSG00000174136	RGMB	285704	-0.80	4.33E-08	1.61E-05
Treatment: Day 3	ENSG00000101825	MXRA5	25878	-1.25	5.30E-08	1.92E-05
Treatment: Day 3	ENSG00000174697	LEP	3952	3.32	5.61E-08	1.99E-05
Treatment: Day 3	ENSG00000087245	MMP2	4313	-0.71	7.03E-08	2.44E-05
Treatment: Day 3	ENSG00000179388	EGR3	1960	-2.05	7.40E-08	2.46E-05
Treatment: Day 3	ENSG00000108960	MMD	23531	0.98	7.42E-08	2.46E-05

Treatment: Day 3	ENSG00000137809	ITGA11	22801	-2.55	7.97E-08	2.54E-05
Treatment: Day 3	ENSG00000169122	FAM110B	90362	-0.73	8.08E-08	2.54E-05
Treatment: Day 3	ENSG00000182197	EXT1	2131	-0.57	8.15E-08	2.54E-05
Treatment: Day 3	ENSG00000120708	TGFBI	7045	-0.89	8.41E-08	2.56E-05
Treatment: Day 3	ENSG00000139329	LUM	4060	-1.15	8.58E-08	2.56E-05
Treatment: Day 3	ENSG00000164929	BAALC	79870	-1.87	8.73E-08	2.56E-05
Treatment: Day 3	ENSG00000125148	MT2A	4502	1.45	8.94E-08	2.56E-05
Treatment: Day 3	ENSG00000183287	CCBE1	147372	0.61	9.08E-08	2.56E-05
Treatment: Day 3	ENSG00000141150	RASL10B	91608	1.15	9.85E-08	2.73E-05
Treatment: Day 3	ENSG00000146555	SDK1	221935	-1.21	1.08E-07	2.95E-05
Treatment: Day 3	ENSG00000168398	BDKRB2	624	-1.00	1.21E-07	3.23E-05
Treatment: Day 3	ENSG00000132170	PPARG	5468	0.66	1.34E-07	3.51E-05
Treatment: Day 3	ENSG00000119714	GPR68	8111	-1.40	1.36E-07	3.51E-05
Treatment: Day 3	ENSG00000146250	PRSS35	167681	-1.63	1.42E-07	3.53E-05
Treatment: Day 3	ENSG00000125675	GRIA3	2892	-0.74	1.42E-07	3.53E-05
Treatment: Day 3	ENSG00000157368	IL34	146433	-1.98	1.44E-07	3.53E-05
Treatment: Day 3	ENSG00000166741	NNMT	4837	0.89	1.64E-07	3.96E-05
Treatment: Day 3	ENSG00000164104	HMGB2	3148	0.78	1.71E-07	4.08E-05
Treatment: Day 3	ENSG00000163491	NEK10	152110	-1.36	1.85E-07	4.24E-05
Treatment: Day 3	ENSG00000103381	CPPED1	55313	0.60	1.86E-07	4.24E-05
Treatment: Day 3	ENSG00000121039	RDH10	157506	-1.03	1.86E-07	4.24E-05
Treatment: Day 3	ENSG00000180875	GREM2	64388	-1.07	2.00E-07	4.48E-05
Treatment: Day 3	ENSG00000150630	VEGFC	7424	-0.74	2.03E-07	4.48E-05
Treatment: Day 3	ENSG00000187210	GCNT1	2650	0.81	2.06E-07	4.48E-05
Treatment: Day 3	ENSG00000136859	ANGPTL2	23452	-0.91	2.11E-07	4.52E-05
Treatment: Day 3	ENSG00000138385	SSB	6741	0.64	2.20E-07	4.67E-05
Treatment: Day 3	ENSG00000092969	TGFB2	7042	-1.02	2.32E-07	4.82E-05
Treatment: Day 3	ENSG00000168140	VASN	114990	-0.62	2.35E-07	4.82E-05
Treatment: Day 3	ENSG00000205364	MT1M	4499	1.46	2.38E-07	4.82E-05
Treatment: Day 3	ENSG00000108797	CNTNAP1	8506	-0.75	2.40E-07	4.82E-05
Treatment: Day 3	ENSG00000162692	VCAM1	7412	-1.70	2.44E-07	4.83E-05
Treatment: Day 3	ENSG00000115461	IGFBP5	3488	-1.45	2.53E-07	4.94E-05
Treatment: Day 3	ENSG00000142731	PLK4	10733	0.73	2.74E-07	5.24E-05
Treatment: Day 3	ENSG00000071539	TRIP13	9319	0.86	2.75E-07	5.24E-05
Treatment: Day 3	ENSG00000095303	PTGS1	5742	1.11	2.80E-07	5.28E-05
Treatment: Day 3	ENSG00000113368	LMNB1	4001	0.85	2.93E-07	5.44E-05
Treatment: Day 3	ENSG00000158716	DUSP23	54935	0.85	3.14E-07	5.77E-05
Treatment: Day 3	ENSG00000041982	TNC	3371	-1.27	3.18E-07	5.77E-05
Treatment: Day 3	ENSG00000145777	TSLP	85480	-1.45	3.22E-07	5.77E-05
Treatment: Day 3	ENSG00000128342	LIF	3976	-1.74	3.26E-07	5.78E-05
Treatment: Day 3	ENSG00000100739	BDKRB1	623	-1.27	3.32E-07	5.79E-05
Treatment: Day 3	ENSG00000087494	PTHLH	5744	-1.53	3.34E-07	5.79E-05

Treatment: Day 3	ENSG00000128045	RASL11B	65997	1.57	3.44E-07	5.90E-05
Treatment: Day 3	ENSG00000164109	MAD2L1	4085	0.73	3.77E-07	6.35E-05
Treatment: Day 3	ENSG00000188042	ARL4C	10123	-1.06	3.79E-07	6.35E-05
Treatment: Day 3	ENSG00000049759	NEDD4L	23327	-0.67	3.88E-07	6.43E-05
Treatment: Day 3	ENSG00000187193	MT1X	4501	1.82	3.93E-07	6.45E-05
Treatment: Day 3	ENSG00000109472	CPE	1363	-0.81	3.98E-07	6.46E-05
Treatment: Day 3	ENSG00000188157	AGRN	375790	-0.69	4.27E-07	6.85E-05
Treatment: Day 3	ENSG00000113369	ARRDC3	57561	-0.57	4.37E-07	6.94E-05
Treatment: Day 3	ENSG00000171246	NPTX1	4884	-2.48	4.47E-07	6.98E-05
Treatment: Day 3	ENSG00000162104	ADCY9	115	-0.67	4.48E-07	6.98E-05
Treatment: Day 3	ENSG00000123243	ITIH5	80760	-1.96	4.57E-07	7.03E-05
Treatment: Day 3	ENSG00000170390	DCLK2	166614	-0.73	4.75E-07	7.25E-05
Treatment: Day 3	ENSG00000134057	CCNB1	891	0.72	4.83E-07	7.27E-05
Treatment: Day 3	ENSG00000180155	LYNX1	66004	-0.67	4.86E-07	7.27E-05
Treatment: Day 3	ENSG00000104738	MCM4	4173	0.71	5.02E-07	7.35E-05
Treatment: Day 3	ENSG00000147852	VLDLR	7436	-1.61	5.04E-07	7.35E-05
Treatment: Day 3	ENSG00000123179	EBPL	84650	0.65	5.06E-07	7.35E-05
Treatment: Day 3	ENSG00000162616	DNAJB4	11080	0.66	5.31E-07	7.64E-05
Treatment: Day 3	ENSG00000260549	MT1L	4500	1.61	5.39E-07	7.68E-05
Treatment: Day 3	ENSG00000064601	CTSA	5476	-0.53	5.46E-07	7.71E-05
Treatment: Day 3	ENSG00000138669	PRKG2	5593	-1.40	5.55E-07	7.76E-05
Treatment: Day 3	ENSG00000067177	PHKA1	5255	0.74	5.78E-07	8.02E-05
Treatment: Day 3	ENSG00000196878	LAMB3	3914	-0.61	5.99E-07	8.22E-05
Treatment: Day 3	ENSG00000119969	HELLS	3070	0.75	6.10E-07	8.31E-05
Treatment: Day 3	ENSG00000155760	FZD7	8324	-0.76	6.35E-07	8.57E-05
Treatment: Day 3	ENSG00000163584	RPL22L1	200916	0.74	6.64E-07	8.86E-05
Treatment: Day 3	ENSG00000179314	WSCD1	23302	1.13	6.72E-07	8.86E-05
Treatment: Day 3	ENSG00000113657	DPYSL3	1809	-0.52	6.74E-07	8.86E-05
Treatment: Day 3	ENSG00000163431	LMOD1	25802	-0.64	7.09E-07	9.19E-05
Treatment: Day 3	ENSG00000144381	HSPD1	3329	0.59	7.12E-07	9.19E-05
Treatment: Day 3	ENSG00000166448	TMEM130	222865	-1.14	7.37E-07	9.44E-05
Treatment: Day 3	ENSG00000134339	SAA2	6289	2.41	7.63E-07	9.70E-05
Treatment: Day 3	ENSG00000114098	ARMC8	25852	0.48	8.36E-07	0.000105366
Treatment: Day 3	ENSG00000166341	DCHS1	8642	-0.88	8.46E-07	0.000105783
Treatment: Day 3	ENSG00000152953	STK32B	55351	-0.61	8.92E-07	0.000110607
Treatment: Day 3	ENSG00000117399	CDC20	991	0.81	9.07E-07	0.000111149
Treatment: Day 3	ENSG00000103742	IGDCC4	57722	-0.90	9.19E-07	0.000111149
Treatment: Day 3	ENSG00000171867	PRNP	5621	-0.79	9.23E-07	0.000111149
Treatment: Day 3	ENSG00000189058	APOD	347	1.39	9.26E-07	0.000111149
Treatment: Day 3	ENSG00000171522	PTGER4	5734	1.05	9.41E-07	0.000112031
Treatment: Day 3	ENSG00000228716	DHFR	1719	0.71	9.48E-07	0.000112031
Treatment: Day 3	ENSG00000164379	FOXQ1	94234	-1.28	9.73E-07	0.000112901

Treatment: Day 3	ENSG00000080573	COL5A3	50509	-0.69	9.74E-07	0.000112901
Treatment: Day 3	ENSG00000154309	DISP1	84976	-0.85	9.77E-07	0.000112901
Treatment: Day 3	ENSG00000198932	GPRASP1	9737	-0.89	1.04E-06	0.000118767
Treatment: Day 3	ENSG00000131153	GINS2	51659	0.89	1.09E-06	0.000123747
Treatment: Day 3	ENSG00000253910	PCDHGB2	56103	-0.78	1.10E-06	0.0001245
Treatment: Day 3	ENSG00000261373	VPS9D1-AS1	100128881	1.17	1.11E-06	0.0001245
Treatment: Day 3	ENSG00000035499	DEPDC1B	55789	0.84	1.13E-06	0.000125341
Treatment: Day 3	ENSG00000140285	FGF7	2252	-0.56	1.17E-06	0.000128196
Treatment: Day 3	ENSG00000105499	PLA2G4C	8605	-1.03	1.18E-06	0.000128196
Treatment: Day 3	ENSG00000158258	CLSTN2	64084	-1.65	1.18E-06	0.000128196
Treatment: Day 3	ENSG00000135919	SERPINE2	5270	-1.00	1.19E-06	0.000128196
Treatment: Day 3	ENSG00000204525	HLA-C	3107	-0.54	1.19E-06	0.000128196
Treatment: Day 3	ENSG00000123213	NLN	57486	0.42	1.22E-06	0.000130489
Treatment: Day 3	ENSG00000116260	QSOX1	5768	-0.52	1.26E-06	0.000132877
Treatment: Day 3	ENSG00000178031	ADAMTSL1	92949	-0.91	1.26E-06	0.000132877
Treatment: Day 3	ENSG00000137501	SYTL2	54843	-1.13	1.28E-06	0.000133968
Treatment: Day 3	ENSG00000163131	CTSS	1520	-0.69	1.31E-06	0.000135992
Treatment: Day 3	ENSG00000059377	TBXAS1	6916	0.91	1.33E-06	0.000137192
Treatment: Day 3	ENSG00000141448	GATA6	2627	-1.37	1.39E-06	0.000141807
Treatment: Day 3	ENSG00000110492	MDK	4192	-0.75	1.41E-06	0.000141991
Treatment: Day 3	ENSG00000267365	KCNJ2-AS1	400617	-1.04	1.41E-06	0.000141991
Treatment: Day 3	ENSG00000197635	DPP4	1803	-0.69	1.42E-06	0.000141991
Treatment: Day 3	ENSG00000172403	SYNPO2	171024	-1.13	1.43E-06	0.000142609
Treatment: Day 3	ENSG00000164649	CDCA7L	55536	0.66	1.44E-06	0.00014274
Treatment: Day 3	ENSG00000123737	EXOSC9	5393	0.60	1.45E-06	0.00014274
Treatment: Day 3	ENSG00000127954	STEAP4	79689	2.53	1.47E-06	0.000143967
Treatment: Day 3	ENSG00000006747	SCIN	85477	-1.51	1.48E-06	0.000144083
Treatment: Day 3	ENSG00000115844	DLX2	1746	-0.92	1.52E-06	0.000146366
Treatment: Day 3	ENSG00000179954	SSC5D	284297	-0.68	1.53E-06	0.000147025
Treatment: Day 3	ENSG00000168309	FAM107A	11170	1.99	1.56E-06	0.000147861
Treatment: Day 3	ENSG00000253731	PCDHGA6	56109	-0.79	1.56E-06	0.000147861
Treatment: Day 3	ENSG00000145386	CCNA2	890	0.77	1.64E-06	0.000152592
Treatment: Day 3	ENSG00000106617	PRKAG2	51422	0.63	1.65E-06	0.000152592
Treatment: Day 3	ENSG00000185565	LSAMP	4045	-0.95	1.65E-06	0.000152592
Treatment: Day 3	ENSG00000170214	ADRA1B	147	1.16	1.65E-06	0.000152592
Treatment: Day 3	ENSG00000113580	NR3C1	2908	-0.66	1.66E-06	0.000152649
Treatment: Day 3	ENSG00000109610	SOD3	6649	-1.02	1.67E-06	0.000152649
Treatment: Day 3	ENSG00000196584	XRCC2	7516	0.90	1.68E-06	0.000152782
Treatment: Day 3	ENSG00000176697	BDNF	627	-0.68	1.70E-06	0.000153701
Treatment: Day 3	ENSG00000184220	CMSS1	84319	0.58	1.71E-06	0.000153701
Treatment: Day 3	ENSG00000177169	ULK1	8408	-0.45	1.75E-06	0.000155937
Treatment: Day 3	ENSG00000140937	CDH11	1009	-0.57	1.76E-06	0.000155937

Treatment: Day 3	ENSG00000169744	LDB2	9079	-0.92	1.77E-06	0.000156375
Treatment: Day 3	ENSG00000198612	COPS8	10920	0.44	1.80E-06	0.000157709
Treatment: Day 3	ENSG00000165617	DACT1	51339	-1.06	1.82E-06	0.00015871
Treatment: Day 3	ENSG00000145244	CORIN	10699	2.99	1.88E-06	0.000162722
Treatment: Day 3	ENSG00000137266	SLC22A23	63027	-0.89	1.91E-06	0.00016382
Treatment: Day 3	ENSG00000172348	RCAN2	10231	-0.95	1.91E-06	0.00016382
Treatment: Day 3	ENSG00000164611	PTTG1	9232	0.78	1.96E-06	0.000164402
Treatment: Day 3	ENSG00000137869	CYP19A1	1588	1.42	1.97E-06	0.000164402
Treatment: Day 3	ENSG00000255717	SNHG1	23642	0.54	1.98E-06	0.000164402
Treatment: Day 3	ENSG00000197405	C5AR1	728	1.82	1.98E-06	0.000164402
Treatment: Day 3	ENSG00000184254	ALDH1A3	220	-0.78	1.99E-06	0.000164402
Treatment: Day 3	ENSG00000157445	CACNA2D3	55799	-1.01	2.00E-06	0.000164402
Treatment: Day 3	ENSG00000076382	SPAG5	10615	0.73	2.01E-06	0.000164402
Treatment: Day 3	ENSG00000121898	CPXM2	119587	-0.57	2.01E-06	0.000164402
Treatment: Day 3	ENSG00000154654	NCAM2	4685	-1.04	2.02E-06	0.000164402
Treatment: Day 3	ENSG00000147100	SLC16A2	6567	-0.50	2.08E-06	0.000168549
Treatment: Day 3	ENSG00000123700	KCNJ2	3759	-0.91	2.10E-06	0.000169618
Treatment: Day 3	ENSG00000204634	TBC1D8	11138	0.72	2.15E-06	0.000170991
Treatment: Day 3	ENSG00000168078	PBK	55872	0.77	2.15E-06	0.000170991
Treatment: Day 3	ENSG00000172889	EGFL7	51162	-0.93	2.15E-06	0.000170991
Treatment: Day 3	ENSG00000130513	GDF15	9518	-1.16	2.25E-06	0.000177793
Treatment: Day 3	ENSG00000234745	HLA-B	3106	-0.61	2.27E-06	0.000178479
Treatment: Day 3	ENSG00000144354	CDCA7	83879	0.61	2.29E-06	0.000179267
Treatment: Day 3	ENSG00000112984	KIF20A	10112	0.69	2.41E-06	0.000187501
Treatment: Day 3	ENSG00000040275	SPDL1	54908	0.55	2.42E-06	0.000187501
Treatment: Day 3	ENSG00000077943	ITGA8	8516	-1.23	2.44E-06	0.00018778
Treatment: Day 3	ENSG00000143815	LBR	3930	0.48	2.45E-06	0.00018778
Treatment: Day 3	ENSG00000087586	AURKA	6790	0.59	2.47E-06	0.00018778
Treatment: Day 3	ENSG00000111424	VDR	7421	-0.51	2.48E-06	0.00018778
Treatment: Day 3	ENSG00000148848	ADAM12	8038	-0.81	2.52E-06	0.000189499
Treatment: Day 3	ENSG00000144802	NFKBIZ	64332	-0.62	2.54E-06	0.000189499
Treatment: Day 3	ENSG00000226950	DANCR	57291	0.81	2.54E-06	0.000189499
Treatment: Day 3	ENSG00000109790	KLHL5	51088	-0.70	2.55E-06	0.000189499
Treatment: Day 3	ENSG00000108561	C1QBP	708	0.52	2.62E-06	0.000193678
Treatment: Day 3	ENSG00000013293	SLC7A14	57709	-2.24	2.63E-06	0.000193842
Treatment: Day 3	ENSG00000106976	DNM1	1759	-0.77	2.68E-06	0.000196281
Treatment: Day 3	ENSG00000185633	NDUFA4L2	56901	-2.48	2.72E-06	0.000198336
Treatment: Day 3	ENSG00000169855	ROBO1	6091	-0.74	2.81E-06	0.000202968
Treatment: Day 3	ENSG00000073756	PTGS2	5743	-1.43	2.81E-06	0.000202968
Treatment: Day 3	ENSG00000134198	TSPAN2	10100	-1.33	2.82E-06	0.000202968
Treatment: Day 3	ENSG00000166482	MFAP4	4239	-1.10	2.88E-06	0.000205848
Treatment: Day 3	ENSG00000151725	CENPU	79682	0.62	2.90E-06	0.000205848

Treatment: Day 3	ENSG00000176890	TYMS	7298	0.78	2.91E-06	0.000205848
Treatment: Day 3	ENSG00000180198	RCC1	1104	0.68	2.93E-06	0.000205848
Treatment: Day 3	ENSG00000134531	EMP1	2012	0.47	2.93E-06	0.000205848
Treatment: Day 3	ENSG00000054598	FOXC1	2296	-0.84	2.97E-06	0.000207133
Treatment: Day 3	ENSG00000189056	RELN	5649	-1.65	2.98E-06	0.000207133
Treatment: Day 3	ENSG00000161921	CXCL16	58191	-0.93	3.01E-06	0.000208278
Treatment: Day 3	ENSG00000162852	CNST	163882	-0.39	3.03E-06	0.000209082
Treatment: Day 3	ENSG00000139364	TMEM132B	114795	-1.38	3.19E-06	0.000218152
Treatment: Day 3	ENSG00000176974	SHMT1	6470	0.91	3.19E-06	0.000218152
Treatment: Day 3	ENSG00000107949	BCCIP	56647	0.44	3.23E-06	0.000219968
Treatment: Day 3	ENSG00000183160	TMEM119	338773	-0.63	3.32E-06	0.000223973
Treatment: Day 3	ENSG00000182389	CACNB4	785	-1.54	3.32E-06	0.000223973
Treatment: Day 3	ENSG00000108604	SMARCD2	6603	0.46	3.33E-06	0.000223973
Treatment: Day 3	ENSG00000099860	GADD45B	4616	0.68	3.37E-06	0.000224513
Treatment: Day 3	ENSG00000183578	TNFAIP8L3	388121	1.04	3.38E-06	0.000224513
Treatment: Day 3	ENSG00000006016	CRLF1	9244	-0.97	3.39E-06	0.000224513
Treatment: Day 3	ENSG00000171552	BCL2L1	598	0.45	3.40E-06	0.000224649
Treatment: Day 3	ENSG00000131370	SH3BP5	9467	-0.46	3.50E-06	0.000229741
Treatment: Day 3	ENSG00000181754	AMIGO1	57463	-0.77	3.57E-06	0.000233335
Treatment: Day 3	ENSG00000145147	SLIT2	9353	-0.61	3.59E-06	0.000234124
Treatment: Day 3	ENSG00000182492	BGN	633	-0.45	3.65E-06	0.000237104
Treatment: Day 3	ENSG00000073111	MCM2	4171	0.81	3.78E-06	0.00024424
Treatment: Day 3	ENSG00000154864	PIEZO2	63895	-1.82	3.93E-06	0.000252856
Treatment: Day 3	ENSG00000088325	TPX2	22974	0.66	4.05E-06	0.000259284
Treatment: Day 3	ENSG00000157456	CCNB2	9133	0.73	4.10E-06	0.000261819
Treatment: Day 3	ENSG00000101003	GINS1	9837	0.71	4.21E-06	0.000267442
Treatment: Day 3	ENSG00000089685	BIRC5	332	0.85	4.24E-06	0.000268237
Treatment: Day 3	ENSG00000121671	CRY2	1408	-0.54	4.37E-06	0.000274875
Treatment: Day 3	ENSG00000105696	TMEM59L	25789	-0.92	4.38E-06	0.000274875
Treatment: Day 3	ENSG00000143228	NUF2	83540	0.78	4.42E-06	0.000275848
Treatment: Day 3	ENSG00000164099	PRSS12	8492	-0.66	4.43E-06	0.000275848
Treatment: Day 3	ENSG00000143816	WNT9A	7483	-0.78	4.46E-06	0.000276356
Treatment: Day 3	ENSG00000166923	GREM1	26585	-0.58	4.53E-06	0.000279715
Treatment: Day 3	ENSG00000196963	PCDHB16	57717	-1.08	4.57E-06	0.000280922
Treatment: Day 3	ENSG00000132423	COQ3	51805	0.78	4.65E-06	0.000284392
Treatment: Day 3	ENSG00000124145	SDC4	6385	-0.42	4.66E-06	0.000284392
Treatment: Day 3	ENSG00000112902	SEMA5A	9037	-0.86	4.68E-06	0.000284392
Treatment: Day 3	ENSG00000065534	MYLK	4638	-0.60	4.71E-06	0.000284749
Treatment: Day 3	ENSG00000164619	BMPER	168667	-0.92	4.80E-06	0.000289425
Treatment: Day 3	ENSG00000106123	EPHB6	2051	0.85	4.86E-06	0.000291785
Treatment: Day 3	ENSG00000198056	PRIM1	5557	0.67	4.97E-06	0.000297131
Treatment: Day 3	ENSG00000174371	EXO1	9156	0.79	5.13E-06	0.000304961

Treatment: Day 3	ENSG00000100342	APOL1	8542	-0.68	5.17E-06	0.000304961
Treatment: Day 3	ENSG00000126787	DLGAP5	9787	0.77	5.18E-06	0.000304961
Treatment: Day 3	ENSG00000140416	TPM1	7168	-0.55	5.21E-06	0.000304961
Treatment: Day 3	ENSG00000132603	NIP7	51388	0.43	5.21E-06	0.000304961
Treatment: Day 3	ENSG00000253276	CCDC71L	168455	-0.56	5.22E-06	0.000304961
Treatment: Day 3	ENSG00000143369	ECM1	1893	-0.58	5.43E-06	0.000316064
Treatment: Day 3	ENSG00000100526	CDKN3	1033	0.69	5.71E-06	0.000331196
Treatment: Day 3	ENSG00000105855	ITGB8	3696	-1.23	5.78E-06	0.000333753
Treatment: Day 3	ENSG00000125885	MCM8	84515	0.52	5.86E-06	0.000336046
Treatment: Day 3	ENSG00000115041	KCNIP3	30818	0.72	5.88E-06	0.000336046
Treatment: Day 3	ENSG00000111911	HINT3	135114	-0.48	5.88E-06	0.000336046
Treatment: Day 3	ENSG00000117984	CTSD	1509	-0.46	5.99E-06	0.0003409
Treatment: Day 3	ENSG00000181163	NPM1	4869	0.42	6.08E-06	0.000344338
Treatment: Day 3	ENSG00000101188	NTSR1	4923	-3.32	6.14E-06	0.000346783
Treatment: Day 3	ENSG00000101665	SMAD7	4092	-0.77	6.22E-06	0.000350029
Treatment: Day 3	ENSG00000176845	METRNL	284207	-0.44	6.28E-06	0.000352197
Treatment: Day 3	ENSG00000170373	CST1	1469	-1.33	6.36E-06	0.000354212
Treatment: Day 3	ENSG00000107796	ACTA2	59	-0.64	6.37E-06	0.000354212
Treatment: Day 3	ENSG00000111206	FOXM1	2305	0.63	6.42E-06	0.000356112
Treatment: Day 3	ENSG00000143799	PARP1	142	0.43	6.46E-06	0.000356799
Treatment: Day 3	ENSG00000144369	FAM171B	165215	-0.75	6.54E-06	0.000360057
Treatment: Day 3	ENSG00000131238	PPT1	5538	0.45	6.65E-06	0.000363388
Treatment: Day 3	ENSG00000135424	ITGA7	3679	-0.83	6.67E-06	0.000363388
Treatment: Day 3	ENSG00000073792	IGF2BP2	10644	0.37	6.73E-06	0.000363388
Treatment: Day 3	ENSG00000019582	CD74	972	-0.57	6.74E-06	0.000363388
Treatment: Day 3	ENSG00000198885	ITPRIPL1	150771	1.16	6.75E-06	0.000363388
Treatment: Day 3	ENSG00000168679	SLC16A4	9122	-0.61	6.77E-06	0.000363388
Treatment: Day 3	ENSG00000109072	VTN	7448	-1.54	6.78E-06	0.000363388
Treatment: Day 3	ENSG00000128050	PAICS	10606	0.47	6.81E-06	0.000363388
Treatment: Day 3	ENSG00000185480	PARPBP	55010	0.67	6.82E-06	0.000363388
Treatment: Day 3	ENSG00000198624	CCDC69	26112	0.85	6.84E-06	0.000363388
Treatment: Day 3	ENSG00000171848	RRM2	6241	0.74	6.87E-06	0.000363612
Treatment: Day 3	ENSG00000164694	FNDC1	84624	-1.76	6.89E-06	0.000363612
Treatment: Day 3	ENSG00000214814	FER1L6	654463	-2.04	6.94E-06	0.000364808
Treatment: Day 3	ENSG00000250510	GPR162	27239	-0.84	6.98E-06	0.000365464
Treatment: Day 3	ENSG00000173085	COQ2	27235	0.61	7.02E-06	0.000366485
Treatment: Day 3	ENSG00000024526	DEPDC1	55635	0.73	7.11E-06	0.000370121
Treatment: Day 3	ENSG00000122042	UBL3	5412	-0.56	7.15E-06	0.000370905
Treatment: Day 3	ENSG00000198901	PRC1	9055	0.69	7.24E-06	0.000374101
Treatment: Day 3	ENSG00000124249	KCNK15	60598	-1.80	7.28E-06	0.000374927
Treatment: Day 3	ENSG00000227051	C14orf132	56967	-0.59	7.34E-06	0.000376573
Treatment: Day 3	ENSG00000117593	DARS2	55157	0.40	7.37E-06	0.000377237

Treatment: Day 3	ENSG00000135472	FAIM2	23017	-1.26	7.59E-06	0.000386958
Treatment: Day 3	ENSG00000146918	NCAPG2	54892	0.60	7.62E-06	0.000387393
Treatment: Day 3	ENSG00000148229	POLE3	54107	0.48	7.65E-06	0.000387406
Treatment: Day 3	ENSG00000135451	TROAP	10024	0.84	7.89E-06	0.000398488
Treatment: Day 3	ENSG00000242125	SNHG3	8420	0.69	7.95E-06	0.00040022
Treatment: Day 3	ENSG00000172915	NBEA	26960	-0.79	8.06E-06	0.000404194
Treatment: Day 3	ENSG00000198910	L1CAM	3897	-1.38	8.19E-06	0.000409431
Treatment: Day 3	ENSG00000163918	RFC4	5984	0.52	8.22E-06	0.000409557
Treatment: Day 3	ENSG00000124134	KCNS1	3787	-0.97	8.39E-06	0.000416713
Treatment: Day 3	ENSG00000196932	TMEM26	219623	-1.21	8.47E-06	0.000419167
Treatment: Day 3	ENSG00000149554	CHEK1	1111	0.40	8.53E-06	0.000419167
Treatment: Day 3	ENSG00000159763	PIP	5304	3.01	8.57E-06	0.000419167
Treatment: Day 3	ENSG00000156011	PSD3	23362	-0.97	8.59E-06	0.000419167
Treatment: Day 3	ENSG00000128815	WDFY4	57705	1.87	8.60E-06	0.000419167
Treatment: Day 3	ENSG00000197565	COL4A6	1288	1.12	8.63E-06	0.000419167
Treatment: Day 3	ENSG00000116120	FARSB	10056	0.39	8.63E-06	0.000419167
Treatment: Day 3	ENSG00000089006	SNX5	27131	0.37	8.69E-06	0.000420757
Treatment: Day 3	ENSG00000138411	HECW2	57520	-0.65	8.74E-06	0.000421349
Treatment: Day 3	ENSG00000171241	SHCBP1	79801	0.63	8.77E-06	0.000421349
Treatment: Day 3	ENSG00000151892	GFRA1	2674	-1.13	8.79E-06	0.000421349
Treatment: Day 3	ENSG00000135709	KIAA0513	9764	-0.56	8.90E-06	0.000425182
Treatment: Day 3	ENSG00000068489	PRR11	55771	0.65	9.23E-06	0.000439576
Treatment: Day 3	ENSG00000166197	NOLC1	9221	0.47	9.33E-06	0.000441971
Treatment: Day 3	ENSG00000176490	DIRAS1	148252	-0.75	9.34E-06	0.000441971
Treatment: Day 3	ENSG00000123989	CHPF	79586	-0.53	9.41E-06	0.000441971
Treatment: Day 3	ENSG00000164307	ERAP1	51752	-0.48	9.41E-06	0.000441971
Treatment: Day 3	ENSG00000054277	OPN3	23596	0.60	9.44E-06	0.000441971
Treatment: Day 3	ENSG00000112312	GMNN	51053	0.61	9.45E-06	0.000441971
Treatment: Day 3	ENSG00000136040	PLXNC1	10154	-1.32	9.53E-06	0.000444405
Treatment: Day 3	ENSG00000172346	CSDC2	27254	-0.77	9.68E-06	0.000447776
Treatment: Day 3	ENSG00000072041	SLC6A15	55117	0.76	9.68E-06	0.000447776
Treatment: Day 3	ENSG00000172037	LAMB2	3913	-0.47	9.69E-06	0.000447776
Treatment: Day 3	ENSG00000138735	PDE5A	8654	-0.59	9.79E-06	0.000450749
Treatment: Day 3	ENSG00000136997	MYC	4609	0.79	9.88E-06	0.000453209
Treatment: Day 3	ENSG00000063180	CA11	770	-1.07	9.91E-06	0.000453209
Treatment: Day 3	ENSG00000121957	GPSM2	29899	0.52	9.93E-06	0.000453209
Treatment: Day 3	ENSG00000125637	PSD4	23550	0.66	1.00E-05	0.000453986
Treatment: Day 3	ENSG00000132561	MATN2	4147	-1.25	1.00E-05	0.000453986
Treatment: Day 3	ENSG00000181938	GINS3	64785	0.61	1.01E-05	0.000456832
Treatment: Day 3	ENSG00000221826	PSG3	5671	-2.13	1.01E-05	0.000456832
Treatment: Day 3	ENSG00000166851	PLK1	5347	0.71	1.02E-05	0.000457979
Treatment: Day 3	ENSG00000165304	MELK	9833	0.56	1.02E-05	0.000457979

Treatment: Day 3	ENSG00000166592	RRAD	6236	-0.88	1.03E-05	0.000458477
Treatment: Day 3	ENSG00000134917	ADAMTS8	11095	-1.33	1.04E-05	0.000461496
Treatment: Day 3	ENSG00000113119	TMCO6	55374	0.64	1.04E-05	0.000461843
Treatment: Day 3	ENSG00000105202	FBL	2091	0.64	1.05E-05	0.000463229
Treatment: Day 3	ENSG00000176208	ATAD5	79915	0.68	1.05E-05	0.000463229
Treatment: Day 3	ENSG00000120658	ENOX1	55068	-0.69	1.05E-05	0.000463229
Treatment: Day 3	ENSG00000121152	NCAPH	23397	0.77	1.05E-05	0.000463229
Treatment: Day 3	ENSG00000158467	AHCYL2	23382	-0.78	1.06E-05	0.000463229
Treatment: Day 3	ENSG00000184785	SMIM10	644538	0.61	1.07E-05	0.000465724
