# Supplementary material for: Ascorbic Acid/Retinol and/or Inflammatory Stimuli’s Effect on Proliferation/Differentiation Properties and Transcriptomics of Gingival Stem/Progenitor Cells
Source: Cells. 2021 Nov 25;10(12):3310. doi: 10.3390/cells10123310 (PMC8699152; doi:10.3390/cells10123310)
Supplement: Supplementary file 1 [file cells-10-03310-s001.zip › cells-1436458 supplementary/cells-1436458 Table S2.pdf]

Effect	KEGG_ID	Gene
treatment & medium: Day 1	Cytokine-cytokine receptor interaction	CXCL5
treatment & medium: Day 1	Cytokine-cytokine receptor interaction	CXCL10
treatment & medium: Day 1	Cytokine-cytokine receptor interaction	EBI3
treatment & medium: Day 1	Cytokine-cytokine receptor interaction	PF4
treatment & medium: Day 1	Cytokine-cytokine receptor interaction	CCL5
treatment & medium: Day 1	Cytokine-cytokine receptor interaction	IL32
treatment & medium: Day 1	Cytokine-cytokine receptor interaction	CCL20
treatment & medium: Day 1	Cytokine-cytokine receptor interaction	IL1B
treatment & medium: Day 1	Cytokine-cytokine receptor interaction	CXCL6
treatment & medium: Day 1	Cytokine-cytokine receptor interaction	CXCL2
treatment & medium: Day 1	Cytokine-cytokine receptor interaction	TGFB2
treatment & medium: Day 1	Cytokine-cytokine receptor interaction	CXCL12
treatment & medium: Day 1	Rheumatoid arthritis	CXCL5
treatment & medium: Day 1	Rheumatoid arthritis	CCL5
treatment & medium: Day 1	Rheumatoid arthritis	CCL20
treatment & medium: Day 1	Rheumatoid arthritis	IL1B
treatment & medium: Day 1	Rheumatoid arthritis	CXCL6
treatment & medium: Day 1	Rheumatoid arthritis	CXCL2
treatment & medium: Day 1	Rheumatoid arthritis	TGFB2
treatment & medium: Day 1	Rheumatoid arthritis	CXCL12
treatment & medium: Day 1	Viral protein interaction with cytokine and cytokine receptor	CXCL5
treatment & medium: Day 1	Viral protein interaction with cytokine and cytokine receptor	CXCL10

treatment & medium: Day 1	Viral protein interaction with cytokine and cytokine receptor	PF4
treatment & medium: Day 1	Viral protein interaction with cytokine and cytokine receptor	CCL5
treatment & medium: Day 1	Viral protein interaction with cytokine and cytokine receptor	CCL20
treatment & medium: Day 1	Viral protein interaction with cytokine and cytokine receptor	CXCL6
treatment & medium: Day 1	Viral protein interaction with cytokine and cytokine receptor	CXCL2
treatment & medium: Day 1	Viral protein interaction with cytokine and cytokine receptor	CXCL12
treatment & medium: Day 1	TNF signaling pathway	CXCL5
treatment & medium: Day 1	TNF signaling pathway	CXCL10
treatment & medium: Day 1	TNF signaling pathway	CCL5
treatment & medium: Day 1	TNF signaling pathway	CCL20
treatment & medium: Day 1	TNF signaling pathway	IL1B
treatment & medium: Day 1	TNF signaling pathway	CXCL6
treatment & medium: Day 1	TNF signaling pathway	TNFAIP3
treatment & medium: Day 1	TNF signaling pathway	CXCL2
treatment & medium: Day 1	IL-17 signaling pathway	CXCL5
treatment & medium: Day 1	IL-17 signaling pathway	CXCL10
treatment & medium: Day 1	IL-17 signaling pathway	CCL20
treatment & medium: Day 1	IL-17 signaling pathway	IL1B
treatment & medium: Day 1	IL-17 signaling pathway	CXCL6
treatment & medium: Day 1	IL-17 signaling pathway	TNFAIP3
treatment & medium: Day 1	IL-17 signaling pathway	CXCL2
treatment & medium: Day 1	Chemokine signaling pathway	CXCL5
treatment & medium: Day 1	Chemokine signaling pathway	CXCL10

treatment & medium: Day 1	Chemokine signaling pathway	PF4
treatment & medium: Day 1	Chemokine signaling pathway	CCL5
treatment & medium: Day 1	Chemokine signaling pathway	CCL20
treatment & medium: Day 1	Chemokine signaling pathway	CXCL6
treatment & medium: Day 1	Chemokine signaling pathway	CXCL2
treatment & medium: Day 1	Chemokine signaling pathway	CXCL12
treatment & medium: Day 1	NF-kappa B signaling pathway	IL1B
treatment & medium: Day 1	NF-kappa B signaling pathway	TNFAIP3
treatment & medium: Day 1	NF-kappa B signaling pathway	CXCL2
treatment & medium: Day 1	NF-kappa B signaling pathway	CXCL12
treatment & medium: Day 1	Cytosolic DNA-sensing pathway	CXCL10
treatment & medium: Day 1	Cytosolic DNA-sensing pathway	CCL5
treatment & medium: Day 1	Cytosolic DNA-sensing pathway	IL1B
treatment & medium: Day 1	Inflammatory bowel disease	IL1B
treatment & medium: Day 1	Inflammatory bowel disease	TGFB2
treatment & medium: Day 1	Inflammatory bowel disease	MAF
treatment & medium: Day 1	Drug metabolism - cytochrome P450	ALDH3A1
treatment & medium: Day 1	Drug metabolism - cytochrome P450	FMO2
treatment & medium: Day 1	Drug metabolism - cytochrome P450	FMO1
treatment & medium: Day 1	Pertussis	CXCL5
treatment & medium: Day 1	Pertussis	IL1B
treatment & medium: Day 1	Pertussis	CXCL6

treatment & medium: Day 3	TNF signaling pathway	CXCL5
treatment & medium: Day 3	TNF signaling pathway	CCL20
treatment & medium: Day 3	TNF signaling pathway	CXCL6
treatment & medium: Day 3	TNF signaling pathway	CXCL1
treatment & medium: Day 3	TNF signaling pathway	CCL5
treatment & medium: Day 3	TNF signaling pathway	TNFAIP3
treatment & medium: Day 3	TNF signaling pathway	BIRC3
treatment & medium: Day 3	TNF signaling pathway	VCAM1
treatment & medium: Day 3	TNF signaling pathway	PTGS2
treatment & medium: Day 3	Hypertrophic cardiomyopathy	ITGA10
treatment & medium: Day 3	Hypertrophic cardiomyopathy	CACNB4
treatment & medium: Day 3	Hypertrophic cardiomyopathy	TGFB2
treatment & medium: Day 3	Hypertrophic cardiomyopathy	ITGA8
treatment & medium: Day 3	Hypertrophic cardiomyopathy	ITGA11
treatment & medium: Day 3	Hypertrophic cardiomyopathy	IGF1
treatment & medium: Day 3	Hypertrophic cardiomyopathy	AGT
treatment & medium: Day 3	Rheumatoid arthritis	CXCL5
treatment & medium: Day 3	Rheumatoid arthritis	CCL20
treatment & medium: Day 3	Rheumatoid arthritis	CXCL6
treatment & medium: Day 3	Rheumatoid arthritis	CXCL1
treatment & medium: Day 3	Rheumatoid arthritis	CCL5
treatment & medium: Day 3	Rheumatoid arthritis	TGFB2
treatment & medium: Day 3	Rheumatoid arthritis	CXCL12

treatment & medium: Day 3	Dilated cardiomyopathy	ITGA10
treatment & medium: Day 3	Dilated cardiomyopathy	CACNB4
treatment & medium: Day 3	Dilated cardiomyopathy	TGFB2
treatment & medium: Day 3	Dilated cardiomyopathy	ITGA8
treatment & medium: Day 3	Dilated cardiomyopathy	ITGA11
treatment & medium: Day 3	Dilated cardiomyopathy	IGF1
treatment & medium: Day 3	Dilated cardiomyopathy	AGT
treatment & medium: Day 3	Viral protein interaction with cytokine and cytokine receptor	CXCL5
treatment & medium: Day 3	Viral protein interaction with cytokine and cytokine receptor	CCL20
treatment & medium: Day 3	Viral protein interaction with cytokine and cytokine receptor	CXCL6
treatment & medium: Day 3	Viral protein interaction with cytokine and cytokine receptor	CXCL1
treatment & medium: Day 3	Viral protein interaction with cytokine and cytokine receptor	CCL5
treatment & medium: Day 3	Viral protein interaction with cytokine and cytokine receptor	ACKR3
treatment & medium: Day 3	Viral protein interaction with cytokine and cytokine receptor	CXCL12
treatment & medium: Day 3	Mineral absorption	MT1E
treatment & medium: Day 3	Mineral absorption	MT2A
treatment & medium: Day 3	Mineral absorption	MT1X
treatment & medium: Day 3	Mineral absorption	MT1M
treatment & medium: Day 3	Mineral absorption	SLC40A1
treatment & medium: Day 3	Cytokine-cytokine receptor interaction	CXCL5
treatment & medium: Day 3	Cytokine-cytokine receptor interaction	CCL20
treatment & medium: Day 3	Cytokine-cytokine receptor interaction	CXCL6
treatment & medium: Day 3	Cytokine-cytokine receptor interaction	LEP

treatment & medium: Day 3	Cytokine-cytokine receptor interaction	IL32
treatment & medium: Day 3	Cytokine-cytokine receptor interaction	MSTN
treatment & medium: Day 3	Cytokine-cytokine receptor interaction	CXCL1
treatment & medium: Day 3	Cytokine-cytokine receptor interaction	CCL5
treatment & medium: Day 3	Cytokine-cytokine receptor interaction	ACKR3
treatment & medium: Day 3	Cytokine-cytokine receptor interaction	TGFB2
treatment & medium: Day 3	Cytokine-cytokine receptor interaction	CXCL12
treatment & medium: Day 3	IL-17 signaling pathway	CXCL5
treatment & medium: Day 3	IL-17 signaling pathway	CCL20
treatment & medium: Day 3	IL-17 signaling pathway	CXCL6
treatment & medium: Day 3	IL-17 signaling pathway	CXCL1
treatment & medium: Day 3	IL-17 signaling pathway	TNFAIP3
treatment & medium: Day 3	IL-17 signaling pathway	PTGS2
treatment & medium: Day 3	NF-kappa B signaling pathway	CXCL1
treatment & medium: Day 3	NF-kappa B signaling pathway	TNFAIP3
treatment & medium: Day 3	NF-kappa B signaling pathway	BIRC3
treatment & medium: Day 3	NF-kappa B signaling pathway	VCAM1
treatment & medium: Day 3	NF-kappa B signaling pathway	PTGS2
treatment & medium: Day 3	NF-kappa B signaling pathway	CXCL12
treatment & medium: Day 3	Focal adhesion	ITGA10
treatment & medium: Day 3	Focal adhesion	BIRC3
treatment & medium: Day 3	Focal adhesion	PDGFD
treatment & medium: Day 3	Focal adhesion	VTN

treatment & medium: Day 3	Focal adhesion	ITGA8
treatment & medium: Day 3	Focal adhesion	ITGA11
treatment & medium: Day 3	Focal adhesion	IGF1
treatment & medium: Day 3	Focal adhesion	COMP
medium: Day 1	TNF signaling pathway	CXCL10
medium: Day 1	TNF signaling pathway	IL1B
medium: Day 1	TNF signaling pathway	CCL5
medium: Day 1	TNF signaling pathway	CXCL5
medium: Day 1	TNF signaling pathway	CCL20
medium: Day 1	TNF signaling pathway	BIRC3
medium: Day 1	TNF signaling pathway	MMP3
medium: Day 1	TNF signaling pathway	CXCL6
medium: Day 1	TNF signaling pathway	TNFAIP3
medium: Day 1	TNF signaling pathway	IL6
medium: Day 1	TNF signaling pathway	CXCL3
medium: Day 1	TNF signaling pathway	CXCL2
medium: Day 1	TNF signaling pathway	LIF
medium: Day 1	TNF signaling pathway	CXCL1
medium: Day 1	TNF signaling pathway	CCL2
medium: Day 1	IL-17 signaling pathway	CXCL10
medium: Day 1	IL-17 signaling pathway	IL1B
medium: Day 1	IL-17 signaling pathway	CXCL5
medium: Day 1	IL-17 signaling pathway	CCL20
medium: Day 1	IL-17 signaling pathway	MMP3
medium: Day 1	IL-17 signaling pathway	CXCL6
medium: Day 1	IL-17 signaling pathway	TNFAIP3
medium: Day 1	IL-17 signaling pathway	IL6
medium: Day 1	IL-17 signaling pathway	CCL7
medium: Day 1	IL-17 signaling pathway	CXCL3
medium: Day 1	IL-17 signaling pathway	CXCL2
medium: Day 1	IL-17 signaling pathway	CXCL1
medium: Day 1	IL-17 signaling pathway	CCL2
medium: Day 1	Rheumatoid arthritis	IL1B
medium: Day 1	Rheumatoid arthritis	CCL5
medium: Day 1	Rheumatoid arthritis	CXCL5
medium: Day 1	Rheumatoid arthritis	CCL20
medium: Day 1	Rheumatoid arthritis	MMP3
medium: Day 1	Rheumatoid arthritis	CXCL6

medium: Day 1	Rheumatoid arthritis	IL6
medium: Day 1	Rheumatoid arthritis	CXCL3
medium: Day 1	Rheumatoid arthritis	CXCL2
medium: Day 1	Rheumatoid arthritis	CXCL1
medium: Day 1	Rheumatoid arthritis	CCL2
medium: Day 1	Rheumatoid arthritis	CXCL12
medium: Day 1	Viral protein interaction with cytokine and cytokine receptor	CXCL10
medium: Day 1	Viral protein interaction with cytokine and cytokine receptor	CCL5
medium: Day 1	Viral protein interaction with cytokine and cytokine receptor	CXCL5
medium: Day 1	Viral protein interaction with cytokine and cytokine receptor	CCL20
medium: Day 1	Viral protein interaction with cytokine and cytokine receptor	CXCL6
medium: Day 1	Viral protein interaction with cytokine and cytokine receptor	IL6
medium: Day 1	Viral protein interaction with cytokine and cytokine receptor	CCL7
medium: Day 1	Viral protein interaction with cytokine and cytokine receptor	CXCL3
medium: Day 1	Viral protein interaction with cytokine and cytokine receptor	CXCL2
medium: Day 1	Viral protein interaction with cytokine and cytokine receptor	CXCL1
medium: Day 1	Viral protein interaction with cytokine and cytokine receptor	CCL2
medium: Day 1	Viral protein interaction with cytokine and cytokine receptor	CXCL12
medium: Day 1	Cytokine-cytokine receptor interaction	CXCL10
medium: Day 1	Cytokine-cytokine receptor interaction	IL1B
medium: Day 1	Cytokine-cytokine receptor interaction	CCL5
medium: Day 1	Cytokine-cytokine receptor interaction	CXCL5
medium: Day 1	Cytokine-cytokine receptor interaction	EBI3
medium: Day 1	Cytokine-cytokine receptor interaction	IL32
medium: Day 1	Cytokine-cytokine receptor interaction	CCL20
medium: Day 1	Cytokine-cytokine receptor interaction	TNFRSF8
medium: Day 1	Cytokine-cytokine receptor interaction	CXCL6
medium: Day 1	Cytokine-cytokine receptor interaction	IL6
medium: Day 1	Cytokine-cytokine receptor interaction	CCL7
medium: Day 1	Cytokine-cytokine receptor interaction	CXCL3
medium: Day 1	Cytokine-cytokine receptor interaction	IL33
medium: Day 1	Cytokine-cytokine receptor interaction	CXCL2

medium: Day 1	Cytokine-cytokine receptor interaction	LIF
medium: Day 1	Cytokine-cytokine receptor interaction	CXCL1
medium: Day 1	Cytokine-cytokine receptor interaction	CCL2
medium: Day 1	Cytokine-cytokine receptor interaction	CXCL12
medium: Day 1	Chemokine signaling pathway	CXCL10
medium: Day 1	Chemokine signaling pathway	CCL5
medium: Day 1	Chemokine signaling pathway	CXCL5
medium: Day 1	Chemokine signaling pathway	CCL20
medium: Day 1	Chemokine signaling pathway	CXCL6
medium: Day 1	Chemokine signaling pathway	CCL7
medium: Day 1	Chemokine signaling pathway	CXCL3
medium: Day 1	Chemokine signaling pathway	CXCL2
medium: Day 1	Chemokine signaling pathway	CXCL1
medium: Day 1	Chemokine signaling pathway	CCL2
medium: Day 1	Chemokine signaling pathway	CXCL12
medium: Day 1	Chemokine signaling pathway	PTK2B
medium: Day 1	NOD-like receptor signaling pathway	IL1B
medium: Day 1	NOD-like receptor signaling pathway	CCL5
medium: Day 1	NOD-like receptor signaling pathway	BIRC3
medium: Day 1	NOD-like receptor signaling pathway	TNFAIP3
medium: Day 1	NOD-like receptor signaling pathway	IL6
medium: Day 1	NOD-like receptor signaling pathway	CXCL3
medium: Day 1	NOD-like receptor signaling pathway	CXCL2
medium: Day 1	NOD-like receptor signaling pathway	CXCL1
medium: Day 1	NOD-like receptor signaling pathway	CCL2
medium: Day 1	NF-kappa B signaling pathway	IL1B
medium: Day 1	NF-kappa B signaling pathway	BIRC3
medium: Day 1	NF-kappa B signaling pathway	TNFAIP3
medium: Day 1	NF-kappa B signaling pathway	CXCL3
medium: Day 1	NF-kappa B signaling pathway	CXCL2
medium: Day 1	NF-kappa B signaling pathway	CXCL1
medium: Day 1	NF-kappa B signaling pathway	CXCL12
medium: Day 1	Malaria	IL1B
medium: Day 1	Malaria	IL6
medium: Day 1	Malaria	CCL2
medium: Day 1	Malaria	COMP
medium: Day 1	Malaria	CD36
medium: Day 1	Lipid and atherosclerosis	IL1B
medium: Day 1	Lipid and atherosclerosis	CCL5
medium: Day 1	Lipid and atherosclerosis	MMP3
medium: Day 1	Lipid and atherosclerosis	IL6
medium: Day 1	Lipid and atherosclerosis	CXCL3

medium: Day 1	Lipid and atherosclerosis	CXCL2
medium: Day 1	Lipid and atherosclerosis	CXCL1
medium: Day 1	Lipid and atherosclerosis	CCL2
medium: Day 1	Lipid and atherosclerosis	CD36
medium: Day 1	Legionellosis	IL1B
medium: Day 1	Legionellosis	IL6
medium: Day 1	Legionellosis	CXCL3
medium: Day 1	Legionellosis	CXCL2
medium: Day 1	Legionellosis	CXCL1
medium: Day 1	Cytosolic DNA-sensing pathway	CXCL10
medium: Day 1	Cytosolic DNA-sensing pathway	IL1B
medium: Day 1	Cytosolic DNA-sensing pathway	CCL5
medium: Day 1	Cytosolic DNA-sensing pathway	IL6
medium: Day 1	Cytosolic DNA-sensing pathway	IL33
medium: Day 1	Amoebiasis	IL1B
medium: Day 1	Amoebiasis	IL6
medium: Day 1	Amoebiasis	CXCL3
medium: Day 1	Amoebiasis	CXCL2
medium: Day 1	Amoebiasis	CXCL1
medium: Day 1	Amoebiasis	GNAL
medium: Day 1	Graft-versus-host disease	IL1B
medium: Day 1	Graft-versus-host disease	IL6
medium: Day 1	Graft-versus-host disease	HLA-F
medium: Day 1	Graft-versus-host disease	HLA-B
medium: Day 1	Influenza A	CXCL10
medium: Day 1	Influenza A	IL1B
medium: Day 1	Influenza A	CCL5
medium: Day 1	Influenza A	RSAD2
medium: Day 1	Influenza A	IL6
medium: Day 1	Influenza A	IL33
medium: Day 1	Influenza A	CCL2
medium: Day 1	Human cytomegalovirus infection	IL1B
medium: Day 1	Human cytomegalovirus infection	CCL5
medium: Day 1	Human cytomegalovirus infection	IL6
medium: Day 1	Human cytomegalovirus infection	HLA-F
medium: Day 1	Human cytomegalovirus infection	CCL2
medium: Day 1	Human cytomegalovirus infection	HLA-B
medium: Day 1	Human cytomegalovirus infection	CXCL12
medium: Day 1	Human cytomegalovirus infection	PTK2B
medium: Day 1	Calcium signaling pathway	GRIN2A
medium: Day 1	Calcium signaling pathway	BDKRB1
medium: Day 1	Calcium signaling pathway	PDGFD

medium: Day 1	Calcium signaling pathway	GNAL
medium: Day 1	Calcium signaling pathway	PTK2B
medium: Day 1	Calcium signaling pathway	PDE1A
medium: Day 1	Calcium signaling pathway	FGF18
medium: Day 1	Calcium signaling pathway	PHKG1
medium: Day 1	Folate biosynthesis	AKR1B1
medium: Day 1	Folate biosynthesis	AKR1C3
medium: Day 1	Folate biosynthesis	AKR1B10
medium: Day 1	Arachidonic acid metabolism	GGT5
medium: Day 1	Arachidonic acid metabolism	PLA2G4C
medium: Day 1	Arachidonic acid metabolism	AKR1C3
medium: Day 1	Arachidonic acid metabolism	GPX3
medium: Day 1	Chagas disease	IL1B
medium: Day 1	Chagas disease	CCL5
medium: Day 1	Chagas disease	IL6
medium: Day 1	Chagas disease	CCL2
medium: Day 1	Chagas disease	GNAL
medium: Day 1	Epithelial cell signaling in Helicobacter pylori infection	CCL5
medium: Day 1	Epithelial cell signaling in Helicobacter pylori infection	CXCL3
medium: Day 1	Epithelial cell signaling in Helicobacter pylori infection	CXCL2
medium: Day 1	Epithelial cell signaling in Helicobacter pylori infection	CXCL1
medium: Day 1	Pertussis	IL1B
medium: Day 1	Pertussis	CXCL5
medium: Day 1	Pertussis	CXCL6
medium: Day 1	Pertussis	IL6
medium: Day 1	Antigen processing and presentation	CTSS
medium: Day 1	Antigen processing and presentation	CD74
medium: Day 1	Antigen processing and presentation	HLA-F
medium: Day 1	Antigen processing and presentation	HLA-B
medium: Day 3	TNF signaling pathway	CCL5
medium: Day 3	TNF signaling pathway	CXCL5
medium: Day 3	TNF signaling pathway	CXCL6
medium: Day 3	TNF signaling pathway	BIRC3
medium: Day 3	TNF signaling pathway	TNFAIP3
medium: Day 3	TNF signaling pathway	IL6
medium: Day 3	TNF signaling pathway	ICAM1
medium: Day 3	TNF signaling pathway	LIF
medium: Day 3	TNF signaling pathway	CXCL1
medium: Day 3	TNF signaling pathway	CXCL3
medium: Day 3	Viral protein interaction with cytokine and cytokine receptor	CCL5

medium: Day 3	Viral protein interaction with cytokine and cytokine receptor	CXCL5
medium: Day 3	Viral protein interaction with cytokine and cytokine receptor	CXCL6
medium: Day 3	Viral protein interaction with cytokine and cytokine receptor	IL34
medium: Day 3	Viral protein interaction with cytokine and cytokine receptor	IL6
medium: Day 3	Viral protein interaction with cytokine and cytokine receptor	CXCL1
medium: Day 3	Viral protein interaction with cytokine and cytokine receptor	CXCL3
medium: Day 3	Viral protein interaction with cytokine and cytokine receptor	ACKR3
medium: Day 3	Cytokine-cytokine receptor interaction	CCL5
medium: Day 3	Cytokine-cytokine receptor interaction	EBI3
medium: Day 3	Cytokine-cytokine receptor interaction	CXCL5
medium: Day 3	Cytokine-cytokine receptor interaction	IL32
medium: Day 3	Cytokine-cytokine receptor interaction	CXCL6
medium: Day 3	Cytokine-cytokine receptor interaction	IL34
medium: Day 3	Cytokine-cytokine receptor interaction	IL6
medium: Day 3	Cytokine-cytokine receptor interaction	TSLP
medium: Day 3	Cytokine-cytokine receptor interaction	LIF
medium: Day 3	Cytokine-cytokine receptor interaction	CXCL1
medium: Day 3	Cytokine-cytokine receptor interaction	CXCL3
medium: Day 3	Cytokine-cytokine receptor interaction	ACKR3
medium: Day 3	Rheumatoid arthritis	CCL5
medium: Day 3	Rheumatoid arthritis	CXCL5
medium: Day 3	Rheumatoid arthritis	CXCL6
medium: Day 3	Rheumatoid arthritis	IL6
medium: Day 3	Rheumatoid arthritis	ICAM1
medium: Day 3	Rheumatoid arthritis	CXCL1
medium: Day 3	Rheumatoid arthritis	CXCL3
medium: Day 3	IL-17 signaling pathway	CXCL5
medium: Day 3	IL-17 signaling pathway	CXCL6
medium: Day 3	IL-17 signaling pathway	TNFAIP3
medium: Day 3	IL-17 signaling pathway	IL6
medium: Day 3	IL-17 signaling pathway	CXCL1
medium: Day 3	IL-17 signaling pathway	CXCL3
medium: Day 3	NF-kappa B signaling pathway	BIRC3
medium: Day 3	NF-kappa B signaling pathway	TNFAIP3
medium: Day 3	NF-kappa B signaling pathway	ICAM1
medium: Day 3	NF-kappa B signaling pathway	CXCL1

medium: Day 3	NF-kappa B signaling pathway	CXCL3
medium: Day 3	NF-kappa B signaling pathway	LBP
medium: Day 3	Complement and coagulation cascades	SERPINB2
medium: Day 3	Complement and coagulation cascades	BDKRB1
medium: Day 3	Complement and coagulation cascades	CFB
medium: Day 3	Complement and coagulation cascades	C3
medium: Day 3	Complement and coagulation cascades	A2M
medium: Day 3	Kaposi sarcoma-associated herpesvirus infection	IL6
medium: Day 3	Kaposi sarcoma-associated herpesvirus infection	ICAM1
medium: Day 3	Kaposi sarcoma-associated herpesvirus infection	CXCL1
medium: Day 3	Kaposi sarcoma-associated herpesvirus infection	HLA-F
medium: Day 3	Kaposi sarcoma-associated herpesvirus infection	CXCL3
medium: Day 3	Kaposi sarcoma-associated herpesvirus infection	HLA-B
medium: Day 3	Kaposi sarcoma-associated herpesvirus infection	C3
medium: Day 3	Malaria	IL6
medium: Day 3	Malaria	ICAM1
medium: Day 3	Malaria	CD36
medium: Day 3	Malaria	COMP
medium: Day 3	Phagosome	CTSS
medium: Day 3	Phagosome	HLA-F
medium: Day 3	Phagosome	HLA-B
medium: Day 3	Phagosome	C3
medium: Day 3	Phagosome	CD36
medium: Day 3	Phagosome	COMP
medium: Day 3	Lipid and atherosclerosis	CCL5
medium: Day 3	Lipid and atherosclerosis	IL6
medium: Day 3	Lipid and atherosclerosis	ICAM1
medium: Day 3	Lipid and atherosclerosis	CXCL1
medium: Day 3	Lipid and atherosclerosis	CXCL3
medium: Day 3	Lipid and atherosclerosis	CD36
medium: Day 3	Lipid and atherosclerosis	LBP
medium: Day 3	Legionellosis	IL6
medium: Day 3	Legionellosis	CXCL1
medium: Day 3	Legionellosis	CXCL3
medium: Day 3	Legionellosis	C3
medium: Day 3	NOD-like receptor signaling pathway	CCL5
medium: Day 3	NOD-like receptor signaling pathway	BIRC3
medium: Day 3	NOD-like receptor signaling pathway	TNFAIP3
medium: Day 3	NOD-like receptor signaling pathway	IL6
medium: Day 3	NOD-like receptor signaling pathway	CXCL1
medium: Day 3	NOD-like receptor signaling pathway	CXCL3
medium: Day 3	Chemokine signaling pathway	CCL5

medium: Day 3	Chemokine signaling pathway	CXCL5
medium: Day 3	Chemokine signaling pathway	CXCL6
medium: Day 3	Chemokine signaling pathway	CXCL1
medium: Day 3	Chemokine signaling pathway	CXCL3
medium: Day 3	Chemokine signaling pathway	PTK2B
medium: Day 3	Pertussis	CXCL5
medium: Day 3	Pertussis	CXCL6
medium: Day 3	Pertussis	IL6
medium: Day 3	Pertussis	C3
medium: Day 3	Antigen processing and presentation	CD74
medium: Day 3	Antigen processing and presentation	CTSS
medium: Day 3	Antigen processing and presentation	HLA-F
medium: Day 3	Antigen processing and presentation	HLA-B
medium: Day 3	Graft-versus-host disease	IL6
medium: Day 3	Graft-versus-host disease	HLA-F
medium: Day 3	Graft-versus-host disease	HLA-B
medium: Day 3	Chagas disease	CCL5
medium: Day 3	Chagas disease	IL6
medium: Day 3	Chagas disease	C3
medium: Day 3	Chagas disease	GNAL
medium: Day 3	Amoebiasis	IL6
medium: Day 3	Amoebiasis	CXCL1
medium: Day 3	Amoebiasis	CXCL3
medium: Day 3	Amoebiasis	GNAL
medium: Day 3	Tuberculosis	CD74
medium: Day 3	Tuberculosis	CTSS
medium: Day 3	Tuberculosis	IL6
medium: Day 3	Tuberculosis	C3
medium: Day 3	Tuberculosis	LBP
medium: Day 3	Viral myocarditis	ICAM1
medium: Day 3	Viral myocarditis	HLA-F
medium: Day 3	Viral myocarditis	HLA-B
treatment: Day 1	Cytokine-cytokine receptor interaction	PF4
treatment: Day 1	Cytokine-cytokine receptor interaction	LEP
treatment: Day 1	Cytokine-cytokine receptor interaction	CXCL5
treatment: Day 1	Cytokine-cytokine receptor interaction	BMP2
treatment: Day 1	Cytokine-cytokine receptor interaction	GDF15
treatment: Day 1	Cytokine-cytokine receptor interaction	TGFB2
treatment: Day 1	Cytokine-cytokine receptor interaction	TSLP
treatment: Day 1	Cytokine-cytokine receptor interaction	LIF
treatment: Day 1	Mineral absorption	MT1X

treatment: Day 1	Mineral absorption	MT1M
treatment: Day 1	Mineral absorption	MT2A
treatment: Day 1	Mineral absorption	MT1E
treatment: Day 1	Hypertrophic cardiomyopathy	ITGA10
treatment: Day 1	Hypertrophic cardiomyopathy	TGFB2
treatment: Day 1	Hypertrophic cardiomyopathy	ITGA11
treatment: Day 1	Hypertrophic cardiomyopathy	ITGB8
treatment: Day 1	Dilated cardiomyopathy	ITGA10
treatment: Day 1	Dilated cardiomyopathy	TGFB2
treatment: Day 1	Dilated cardiomyopathy	ITGA11
treatment: Day 1	Dilated cardiomyopathy	ITGB8
treatment: Day 1	Circadian entrainment	PER1
treatment: Day 1	Circadian entrainment	GRIA1
treatment: Day 1	Circadian entrainment	PRKG2
treatment: Day 1	Circadian entrainment	GUCY1A2
treatment: Day 3	ECM-receptor interaction	ITGA10
treatment: Day 3	ECM-receptor interaction	COL4A6
treatment: Day 3	ECM-receptor interaction	ITGB8
treatment: Day 3	ECM-receptor interaction	ITGA8
treatment: Day 3	ECM-receptor interaction	TNC
treatment: Day 3	ECM-receptor interaction	VTN
treatment: Day 3	ECM-receptor interaction	RELN
treatment: Day 3	ECM-receptor interaction	ITGA11
treatment: Day 3	Hypertrophic cardiomyopathy	ITGA10
treatment: Day 3	Hypertrophic cardiomyopathy	CACNA2D3
treatment: Day 3	Hypertrophic cardiomyopathy	TGFB2
treatment: Day 3	Hypertrophic cardiomyopathy	ITGB8
treatment: Day 3	Hypertrophic cardiomyopathy	ITGA8
treatment: Day 3	Hypertrophic cardiomyopathy	CACNB4
treatment: Day 3	Hypertrophic cardiomyopathy	ITGA11
treatment: Day 3	Dilated cardiomyopathy	ITGA10
treatment: Day 3	Dilated cardiomyopathy	CACNA2D3
treatment: Day 3	Dilated cardiomyopathy	TGFB2
treatment: Day 3	Dilated cardiomyopathy	ITGB8
treatment: Day 3	Dilated cardiomyopathy	ITGA8
treatment: Day 3	Dilated cardiomyopathy	CACNB4
treatment: Day 3	Dilated cardiomyopathy	ITGA11
treatment: Day 3	Arrhythmogenic right ventricular cardiomyopathy	ITGA10
treatment: Day 3	Arrhythmogenic right ventricular cardiomyopathy	CACNA2D3
treatment: Day 3	Arrhythmogenic right ventricular cardiomyopathy	ITGB8
treatment: Day 3	Arrhythmogenic right ventricular cardiomyopathy	ITGA8

treatment: Day 3	Arrhythmogenic right ventricular cardiomyopathy	CACNB4
treatment: Day 3	Arrhythmogenic right ventricular cardiomyopathy	ITGA11
treatment: Day 3	Focal adhesion	ITGA10
treatment: Day 3	Focal adhesion	COL4A6
treatment: Day 3	Focal adhesion	ITGB8
treatment: Day 3	Focal adhesion	ITGA8
treatment: Day 3	Focal adhesion	TNC
treatment: Day 3	Focal adhesion	VTN
treatment: Day 3	Focal adhesion	RELN
treatment: Day 3	Focal adhesion	ITGA11
treatment: Day 3	Human papillomavirus infection	ITGA10
treatment: Day 3	Human papillomavirus infection	COL4A6
treatment: Day 3	Human papillomavirus infection	PTGER4
treatment: Day 3	Human papillomavirus infection	ITGB8
treatment: Day 3	Human papillomavirus infection	ITGA8
treatment: Day 3	Human papillomavirus infection	TNC
treatment: Day 3	Human papillomavirus infection	PTGS2
treatment: Day 3	Human papillomavirus infection	VTN
treatment: Day 3	Human papillomavirus infection	RELN
treatment: Day 3	Human papillomavirus infection	ITGA11
treatment: Day 3	Inflammatory mediator regulation of TRP channels	PTGER4
treatment: Day 3	Inflammatory mediator regulation of TRP channels	BDKRB2
treatment: Day 3	Inflammatory mediator regulation of TRP channels	PLA2G4C
treatment: Day 3	Inflammatory mediator regulation of TRP channels	NGF
treatment: Day 3	Inflammatory mediator regulation of TRP channels	BDKRB1
treatment: Day 3	Mineral absorption	MT1X
treatment: Day 3	Mineral absorption	MT1M
treatment: Day 3	Mineral absorption	MT2A
treatment: Day 3	Mineral absorption	MT1E
treatment: Day 3	Regulation of actin cytoskeleton	ITGA10
treatment: Day 3	Regulation of actin cytoskeleton	BDKRB2
treatment: Day 3	Regulation of actin cytoskeleton	ITGB8
treatment: Day 3	Regulation of actin cytoskeleton	ITGA8
treatment: Day 3	Regulation of actin cytoskeleton	BDKRB1
treatment: Day 3	Regulation of actin cytoskeleton	SCIN
treatment: Day 3	Regulation of actin cytoskeleton	ITGA11
treatment: Day 3	PI3K-Akt signaling pathway	ITGA10
treatment: Day 3	PI3K-Akt signaling pathway	COL4A6
treatment: Day 3	PI3K-Akt signaling pathway	ITGB8
treatment: Day 3	PI3K-Akt signaling pathway	ITGA8
treatment: Day 3	PI3K-Akt signaling pathway	NGF
treatment: Day 3	PI3K-Akt signaling pathway	TNC

treatment: Day 3	PI3K-Akt signaling pathway	VTN
treatment: Day 3	PI3K-Akt signaling pathway	RELN
treatment: Day 3	PI3K-Akt signaling pathway	ITGA11
treatment: Day 3	Neuroactive ligand-receptor interaction	LEP
treatment: Day 3	Neuroactive ligand-receptor interaction	C5AR1
treatment: Day 3	Neuroactive ligand-receptor interaction	ADRA1B
treatment: Day 3	Neuroactive ligand-receptor interaction	PTGER4
treatment: Day 3	Neuroactive ligand-receptor interaction	BDKRB2
treatment: Day 3	Neuroactive ligand-receptor interaction	BDKRB1
treatment: Day 3	Neuroactive ligand-receptor interaction	RXFP1
treatment: Day 3	Neuroactive ligand-receptor interaction	NTSR1
treatment: Day 3	Complement and coagulation cascades	C5AR1
treatment: Day 3	Complement and coagulation cascades	BDKRB2
treatment: Day 3	Complement and coagulation cascades	BDKRB1
treatment: Day 3	Complement and coagulation cascades	VTN
