# Supplementary material for: Ascorbic Acid/Retinol and/or Inflammatory Stimuli’s Effect on Proliferation/Differentiation Properties and Transcriptomics of Gingival Stem/Progenitor Cells
Source: Cells. 2021 Nov 25;10(12):3310. doi: 10.3390/cells10123310 (PMC8699152; doi:10.3390/cells10123310)
Supplement: Supplementary file 1 [file cells-10-03310-s001.zip › cells-1436458 supplementary/cells-1436458 Table S3.pdf]

Effect	KEGG	Reactome	Wikipathways
Treatment & medium: Day 1	• Cytokine-cytokine receptor interaction<br>• Rheumatoid arthritis<br>• Viral protein interaction with cytokine and cytokine receptor<br>• TNF signaling pathway<br>• IL-17 signaling pathway<br>• Chemokine signaling pathway<br>• NF-kappa B signaling pathway<br>• Cytosolic DNA-sensing pathway<br>• Inflammatory bowel disease<br>• Drug metabolism - cytochrome P450	• Chemokine receptors bind chemokines<br>• Peptide ligand-binding receptors<br>• Class A/1 (Rhodopsin-like receptors)<br>• Interleukin-10 signaling<br>• G alpha (i) signalling events<br>• Signaling by Interleukins<br>• GPCR ligand binding<br>• Phase I - Functionalization of compounds<br>• Biological oxidations	• SARS-CoV-2 innate immunity evasion and cell-specific immune response<br>• Platelet-mediated interactions with vascular and circulating cells<br>• Nuclear receptors meta-pathway<br>• Chemokine signaling pathway<br>• Interactions between immune cells and microRNAs in tumor microenvironment<br>• Aryl hydrocarbon receptor pathway<br>• IL-18 signaling pathway<br>• Vitamin B12 metabolism<br>• COVID-19 adverse outcome pathway<br>• Lung fibrosis

	KEGG	Reactome	Wikipathways
Treatment & medium: Day 3	• TNF signaling pathway<br>• Hypertrophic cardiomyopathy<br>• Rheumatoid arthritis<br>• Dilated cardiomyopathy<br>• Viral protein interaction with cytokine and cytokine receptor	• Extracellular matrix organization<br>• Molecules associated with elastic fibres<br>• Chemokine receptors bind chemokines<br>• Metallothioneins bind metals<br>• Elastic fibre formation	• Glucocorticoid receptor pathway<br>• Nuclear receptors meta-pathway<br>• Copper homeostasis<br>• Zinc homeostasis<br>• Selenium micronutrient network

- Mineral absorption
- Cytokine-cytokine receptor interaction

- Response to metal ions
- Integrin cell surface interactions
- Peptide ligand-binding receptors
- ECM proteoglycans
- Keratan sulfate degradation
- Platelet degranulation
- GPCR ligand binding
- Response to elevated platelet cytosolic Ca2+
- G alpha (i) signalling events
- Interleukin-10 signaling

- Vitamin B12 metabolism
- Vitamin D receptor pathway
- Oxidative stress response
- Amplification and expansion of oncogenic pathways as metastatic traits
- Platelet-mediated interactions with vascular and circulating cells
- Lung fibrosis
- Endochondral ossification
- Endochondral ossification with skeletal dysplasias
- SARS-CoV-2 innate immunity evasion and cell-specific immune response
- Folate metabolism
- Prostaglandin synthesis and regulation
- Senescence and autophagy in cancer
