# Supplementary figures and images for: Ascorbic Acid/Retinol and/or Inflammatory Stimuli’s Effect on Proliferation/Differentiation Properties and Transcriptomics of Gingival Stem/Progenitor Cells
Source: Cells. 2021 Nov 25;10(12):3310. doi: 10.3390/cells10123310 (PMC8699152; doi:10.3390/cells10123310)

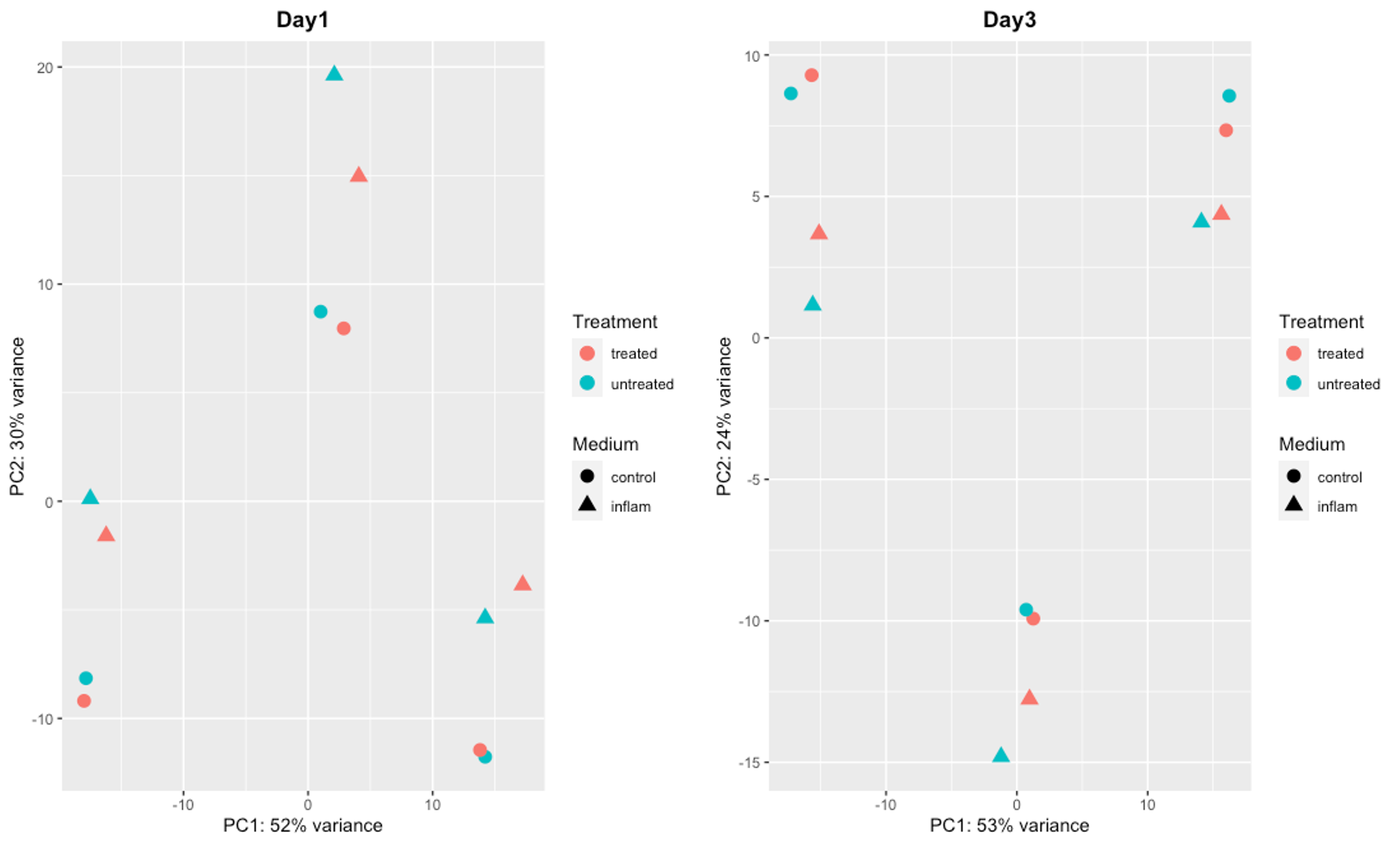

Supplement: Supplementary file 1 [file cells-10-03310-s001.zip › cells-1436458 supplementary/Figure_S1_PCA.png]
